# Multi-level encoding of reward, effort, and choice across the frontal cortex and basal ganglia during cost-benefit decision-making

**DOI:** 10.1016/j.celrep.2024.115209

**Published:** 2025-01-22

**Authors:** Oliver Härmson, Isaac Grennan, Brook Perry, Robert Toth, Colin G. McNamara, Timothy Denison, Hayriye Cagnan, Sanjay G. Manohar, Mark E. Walton, Andrew Sharott

**Affiliations:** 1 Medical Research Council Brain Network Dynamics Unit, Nuffield Department of Clinical Neurosciences, University of Oxford, Oxford, UK; 2 Department of Experimental Psychology, University of Oxford, Oxford, UK; 3 Department of Engineering Science, University of Oxford, Oxford, UK; 4 Nuffield Department of Clinical Neurosciences, University of Oxford, Oxford, UK; 5 Wellcome Centre for Integrative Neuroimaging, University of Oxford, Oxford, UK

**Keywords:** cost-benefit decision-making, cortico-basal ganglia networks, single neurons, ensembles

## Abstract

Adaptive value-guided decision-making requires weighing up the costs and benefits of pursuing an available opportunity. Though neurons across frontal cortical-basal ganglia circuits have been repeatedly shown to represent decision-related parameters, it is unclear whether and how this information is coordinated. To address this question, we performed large-scale single-unit recordings simultaneously across 5 medial/orbital frontal and basal ganglia regions as rats decided whether to pursue varying reward payoffs available at different effort costs. Single neurons encoding combinations of decision variables (reward, effort, and choice) were represented within all recorded regions. Coactive cell assemblies, ensembles of neurons that repeatedly coactivated within short time windows (<25 ms), represented the same decision variables despite the members often having diverse individual coding properties. Together, these findings demonstrate a multi-level encoding structure for cost-benefit computations where individual neurons are coordinated into larger assemblies that can represent task variables independently of their constituent components.

## Highlights

• Neurons were recorded across the frontal-basal ganglia network during cost-benefit decisions
• Firing rate codings of reward, effort, and choice were temporally and spatially heterogeneous
• An emergent temporal code across these neurons is independent of the rate representations
• Rate and temporal codes may support parallel computations within the same circuit

## Graphical abstract

**Figure:**
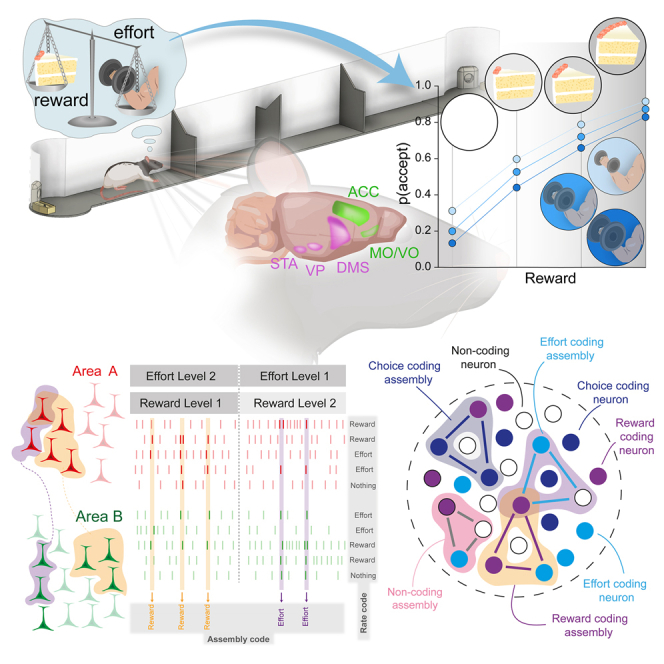

## Teaser

Härmson et al. show that single neurons and coactive assemblies across the medial cortico-basal ganglia circuit encode combinations of reward, effort, and choice during cost-benefit decision-making. Assemblies comprised individual neurons with diverse coding properties, suggesting they can coordinate information into lower-dimensional spaces within and across brain regions.

## Introduction

Deciding whether to invest effort in the pursuit of potential rewards is a challenge that organisms continually face. A wide range of areas across the medial frontal cortical-basal ganglia circuit have been implicated in such cost-benefit decisions.^1^^,^^2^^,^^3^^,^^4^^,^^5^^,^^6^^,^^7^^,^^8^^,^^9^ Single units within individual regions have been shown to code decision-relevant variables such as expected reward value and effort cost.^10^^,^^11^^,^^12^^,^^13^^,^^14^^,^^15^^,^^16^^,^^17^^,^^18^^,^^19^^,^^20^ However, we currently lack a mechanistic understanding of how activity across these frontal and basal ganglia regions accounts for how animals evaluate a beneficial opportunity and regulate whether actions are initiated to pursue that opportunity.

One potential reason is that while work to date has focused on how single neurons in individual regions encode key cost-benefit decision parameters, much less is known about how such coding is *coordinated* across an extended network of brain regions. A common proposition is that single-neuron responses reflect and contribute to modular processing across specialized brain areas that arrive at a behavioral output through serial information transmission.^21^^,^^22^^,^^23^ In sensorimotor tasks, information from the cortex is transferred reliably and topographically to connected areas of the striatum.^24^ However, this need not be the case for cost-benefit calculations, where information presented over different timescales needs to be integrated and then utilized to regulate subsequent choice.

One mechanism to achieve such distributed processing is the formation and expression of co-firing assemblies, whereby groups of neurons repeatedly fire action potentials within a short latency (10–30 ms) of each other.^25^^,^^26^ These assemblies have been demonstrated to play a vital role in population-level representations in areas such as the hippocampus.^27^^,^^28^^,^^29^ Short-timescale coincident spiking can effectively be read out by downstream neural populations on timescales that are optimal for inducing plasticity.^25^^,^^30^ Despite the potential of co-firing assemblies to coordinate neural activity underlying cost-benefit decision-making, it is not clear whether they are formed in cortico-basal ganglia circuits and, if so, whether they can encode relevant variables. Determining this requires simultaneous large-scale recording of neurons across the network during cost-benefit decision-making, which hitherto has rarely been conducted.

To address these questions, we performed simultaneous recordings of single neurons across interconnected frontal and basal ganglia regions—anterior cingulate cortex (ACC), medial/ventral orbitofrontal cortex (MO/VO), dorsal medial striatum (DMS), ventral pallidum (VP), and subthalamic nucleus area (STA)—while rats evaluated whether to accept or reject cost-benefit offers. Individual neurons across the entire network encoded combinations of reward, effort, and choice, with a particularly pronounced phasic and multiplexed signal in DMS and STA. Importantly, co-firing assemblies were also detected within and across all cortico-basal ganglia sites encoding the three main decision parameters independently from the individual firing rates of the member neurons. Remarkably, these often included individual neurons whose firing rates signaled a parameter *different* from the tuning of the overall assembly. Co-firing assemblies thus coordinate individual neurons with wide-ranging coding properties and give rise to emergent representations that can mediate cost-benefit computations at the network level.

## Results

### Choice on a novel rat accept/reject cost-benefit choice paradigm is shaped by anticipated costs and benefits

**Figure 1:**
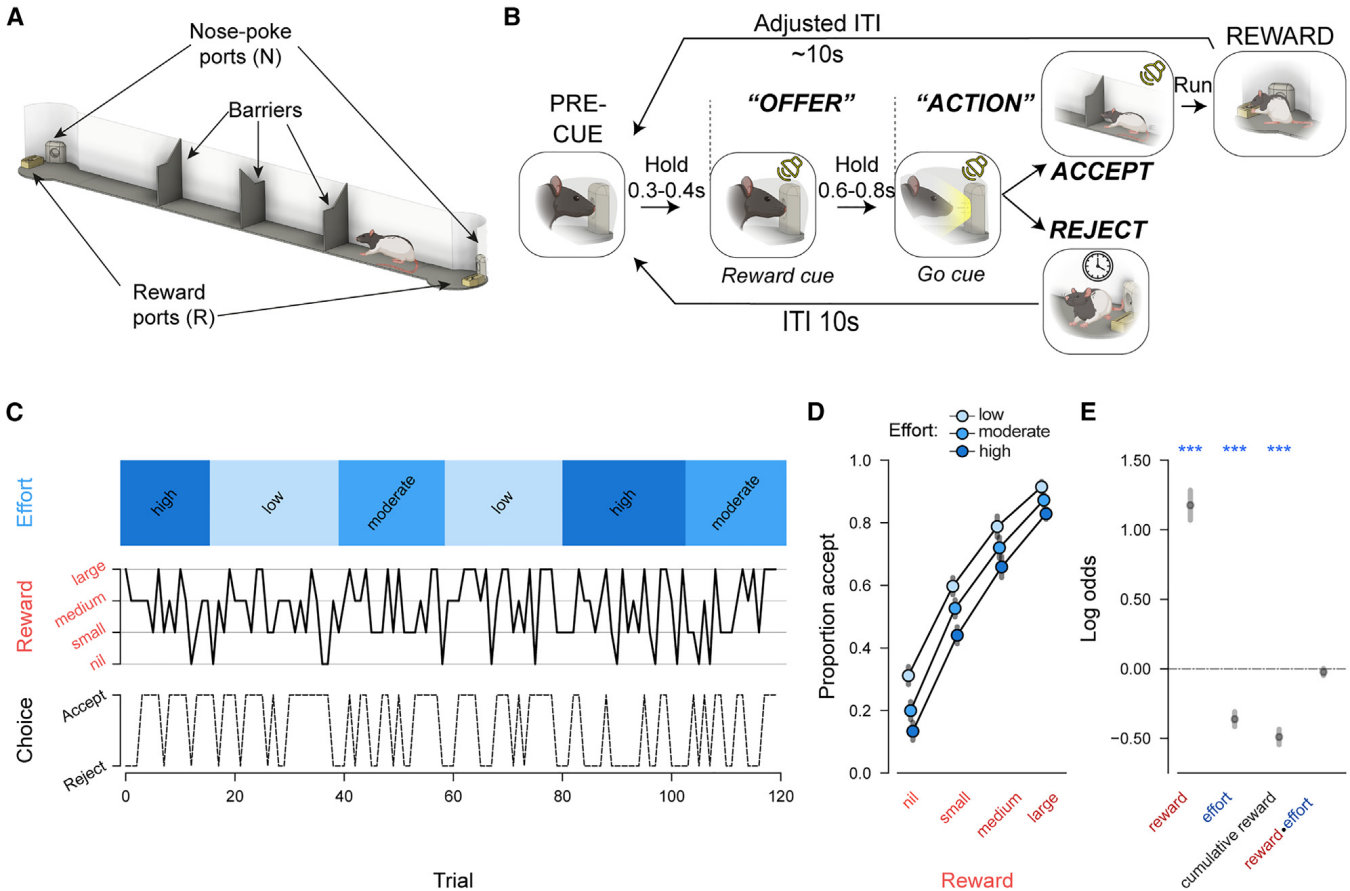
Choice on a novel rat accept/reject choice paradigm is shaped by the costs and benefits (A) Illustration of the experimental apparatus, which consisted of a 130 cm corridor with a variable number of barriers with circular arenas at either end, each of which contained a nose-poke port and a reward port. (B) Rats chose between reward, signaled by an auditory cue during the “offer” window while the rat was in the nose-poke port, available at the far end of a linear corridor (accept) and opting out (reject). To ensure that the average reward rate was minimally affected by rats’ accept/reject choices, ITIs were adjusted on accept trials by subtracting the cohort median run time (STAR Methods). Trials could always be initiated from the same arena where the last trial had finished (same arena on rejected trials; opposite side after an accepted trial). (C) Reward/effort variation and choice in an example session. Reward magnitude (4 levels, middle) is signaled trial by trial by an auditory cue, whereas effort (3 levels, top) is expressed in blocks as the number of barriers in the corridor. Choice is depicted as a dotted line in the bottom image. (D) Rates of accepting offers as a function of reward (x axis) and effort (*n* = 12). Colored dots indicate mean ± SEM, adjusted for the within-subject variance across all levels of effort. (E) Weightings of task variables on behavioral choice from a binomial mixed-effects model, depicted as mean ± SEM. Effect of reward × effort: β = −0.01 ± 0.02, *p* = 0.69, and ^∗∗∗^*p* < 0.001.

To elicit a trade-off between rewards and associated effort costs, we designed a novel cost-benefit task inspired by foraging scenarios. Rats chose whether to pursue or forgo a reward available at the other end of a linear corridor based on its size and the set level of effort (Figure 1A; STAR Methods). Trials were initiated by poking and holding their noses in an initiation port (“pre-cue”) while an auditory cue was presented (“offer”), which signaled the size of the available reward on that trial (4 levels: nil, small, medium, and large; Figure 1B). Once the port light illuminated (“go cue”), rats could then leave the port (“action”) and decide to either (1) pursue the offered reward by running to the opposite end of the corridor (“accept”) or (2) forgo the offered reward by refraining from entering the corridor (“reject”). In both cases, trials were followed by an intertrial interval (ITI), after which a new trial could be initiated. The effort cost of pursuing the reward was manipulated by placing different numbers of barriers in the corridor, fixed across blocks of ∼20 trials (3 levels: low, moderate, and high; Figure 1C) in a pseudo-random order over the recording day. Reward offers were pseudo-randomly varied trial by trial but were repeated after a failed nose-poke attempt.

Rats’ choices were sensitive to both the benefits and costs of acting: animals were more likely to pursue the reward when it was larger and when the effort cost was lower (*n* = 12, non-implanted animals; Figures 1C–1E). This was reflected as a robust positive influence of reward (β = 1.15 ± 0.10, *p* < 0.001, binomial mixed-effects model) and a negative influence of effort (β = −0.33 ± 0.02, *p* < 0.001) over choice (Figure 1E; STAR Methods). Despite the lack of an explicit cue to signal the effort level, it nonetheless had a negative influence on decisions throughout trial blocks (Figure S1A). Rats also progressively accepted fewer offers over the course of a session as they gained more rewards (β = −0.47 ± 0.02, *p* < 0.001), suggesting sensitivity to outcome value (Figure 1E). This was corroborated by a follow-up experiment where rats (*n* = 6) were given free access to lab chow for 1 h prior to testing, which resulted in a significant reduction in their willingness to accept any offer (Figures S1B and S1C), indicating that choices on this task are not reflective of a fixed decision-making policy.

Sensitivity to reward and effort remained significant in a subset (*n* = 4) of rats who were implanted for electrophysiological recordings in an adapted version of this paradigm (Figures S1D and S1E; STAR Methods). Reward offers also consistently influenced action initiation latency on successful trials (Figure S1F) and premature nose-poke exit rates during the offer cue (Figure S1J) in both sets of animals. The rejection of trials once already in the corridor only occurred at a rate of 6.81% ± 3.66% (between-animal median ± SEM) across all trials in implanted rats. Rats' performance on this paradigm was thus robustly sensitive to the offered reward magnitude, effort cost, and outcome value.

### Cost-benefit decision variable representations are asymmetrically distributed across the frontal cortical-basal ganglia network

**Figure 2:**
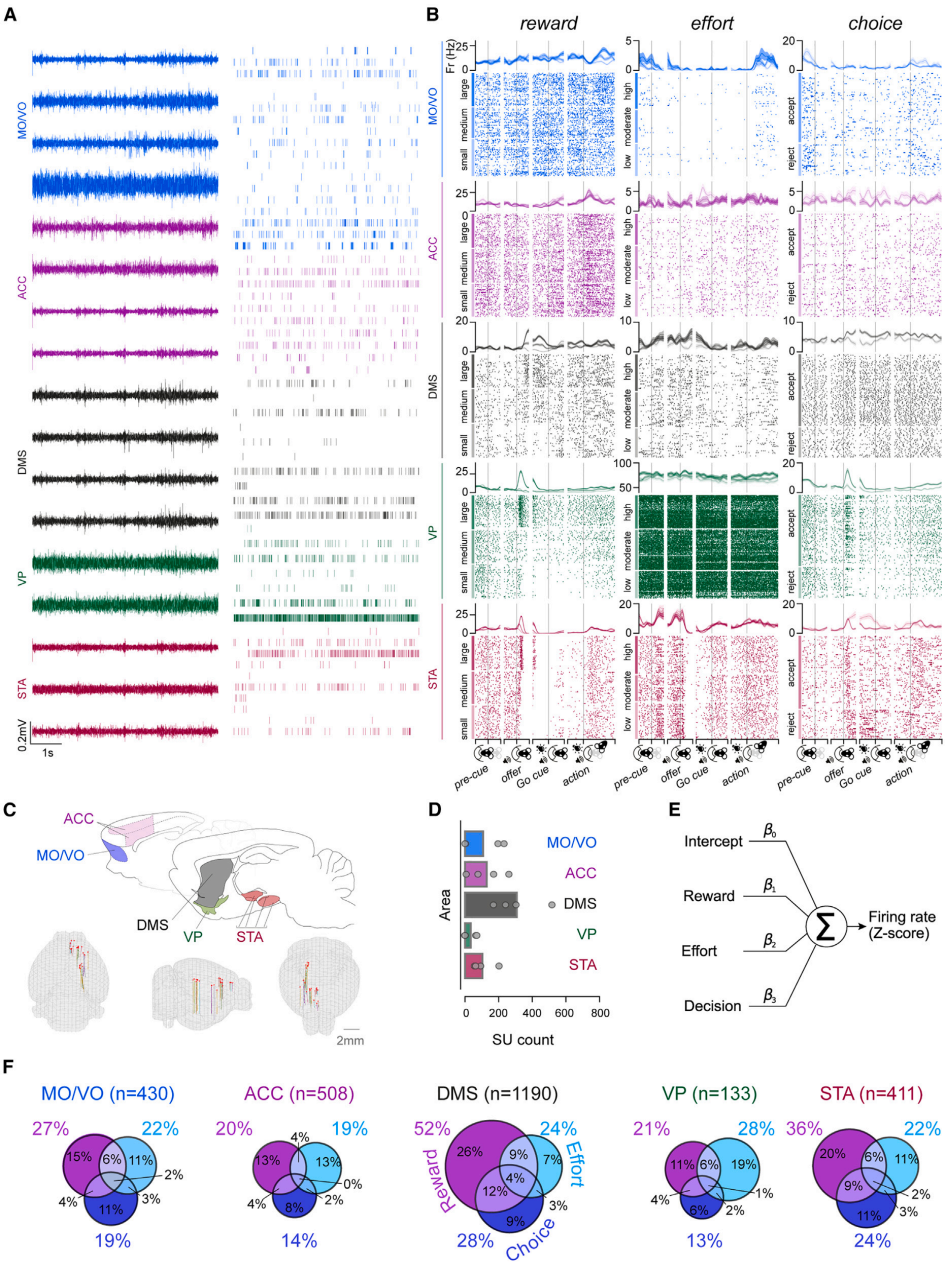
Single neurons encode combinations of reward, effort, and choice across the frontal cortex and basal ganglia (A) High-pass-filtered signal (left) and individual neuron spiking times (right, STAR Methods) displayed from an example five-site simultaneous recording from one example subject. (B) Peri-event time histograms (PSTHs; top) and raster plots (bottom) of 15 individual example neurons (one per image) that monotonically and significantly varied their firing rate as a function of levels of reward, effort, and the rats' eventual choice (STAR Methods). Data are time locked to the beginning of the pre-cue, offer, go cue, and action epochs on the x axes, signified by vertical gray bars. PSTH data are displayed as averages across all other task conditions ± SEM (shaded areas). Data are displayed individually for each trial and grouped from top to bottom by levels of a decision variable, indicated on the left by colored vertical bars. (C) Top: representations of targeted areas on sagittal sections adapted from Paxinos and Watson^52^; bottom: overview of electrode locations on three-dimensional (3D) meshes registered to the Allen common coordinate framework 3D space using SharpTrack,^53^ viewed from the back (left), right side (middle), and front (right). (D) Overview of the average and per-subject single unit (SU) yield in each brain area. Means across subjects are depicted as bars, and subject-by-subject cellular yields are marked as gray dots. We isolated, on average, 108 ± 107 (MO/VO, mean ± standard deviation), 129 ± 96 (ACC), 308 ± 130 (DMS), 34 ± 34 (VP), and 104 ± 58 (STA) SUs per animal. (E) Schematic of the multiple linear regression model used to analyze the contributions of decision variables on individual neuronal firing rates. (F) Proportions of neurons that were tuned to canonical decision variables across a wide time window in each region and had an average firing rate >0.1 Hz (STAR Methods), detected based on significant coefficient of partial determination (CPD) values (*p* < 0.050). Pairwise comparisons for reward-coding neuron proportions: DMS vs. all other structures: all |*Z*| > 5.54, *p* < 0.001, two-sided *Z* test of proportions, adjusted for multiple comparisons using the Benjamini-Hochberg (BH) procedure. STA vs. all structures: all |*Z*| > 2.89, *p* < 0.004. MO/VO vs. ACC: |*Z*| = 2.47, *p* = 0.015. All other |*Z*| < 1.37, *p* > 0.050. Effort-coding neuron proportion comparisons: all |*Z*| < 2.30, *p* > 0.050. Pairwise comparisons for choice-coding proportions: DMS vs. all except STA: |*Z*| > 3.62, *p* < 0.001; STA vs. ACC and VP: both |*Z*| > 2.81, *p* < 0.004.; all other |*Z*| < 2.10, *p* > 0.050. Pairwise comparisons of the summed percentages of all multiplexing neuron types (intersecting Venn diagram areas) between regions: DMS vs. all other regions: all |*Z*| > 3.96, *p* < 0.001; STA vs. MO/VO, ACC, and VP: all |*Z*| > 1.75, *p* < 0.040, one-sided z test of proportions without BH correction. MO/VO, medial/ventral orbitofrontal cortex; ACC, anterior cingulate cortex; DMS, dorsal medial striatum; VP, ventral pallidum; STA, subthalamic nucleus area.

Having established that choices on our task were concurrently sensitive to both reward and effort, we first aimed to identify neural correlates of the underlying computations at the level of single neurons. Given the role of the frontal-basal ganglia network in motivating cost-benefit choice,^4^^,^^9^^,^^17^^,^^31^ we targeted the MO/VO, ACC, DMS, VP, and STA (encompassing the subthalamic nucleus, zona incerta, and substantia nigra pars reticulata and compacta) (Figures 2A–2D, S2, and S3) using a bespoke drivable multielectrode implant to record individual neurons from well-trained rats (*n* = 4) performing the cost-benefit task (STAR Methods). The stereotaxic coordinates in the cortico-basal ganglia network were planned to maximize the possibility of recording from interconnected areas of the MO/VO and ACC and their downstream projections in the DMS^32^ and STA^33^ as well as the VP—a key intermediary nucleus between regions of the striatum and subthalamic nucleus.^34^

We first examined the propensity of cortical and/or basal ganglia neurons to encode single or multiple decision variables. We modeled each identified neuron’s *Z* scored firing rate as the weighted sum of the offered reward, effort, and subsequent accept/reject choice (Figure 2E; STAR Methods) during epochs containing the *entire decision process.* This interval started at offer cue onset and ended 0.9 s before observed corridor entry on accepted trials or 0.9 s before the median of corridor entry latencies on rejected trials (STAR Methods; Figure S4), allowing us to isolate a portion of the trial that was invariant to velocity changes between accepted and rejected trials. Significant proportions of units in each area linearly signaled each canonical decision variable (Figure 2F; all *p* < 0.001, one-sided binomial test against a chance rate of 5% with Benjamini-Hochberg adjustment for multiple tests). Importantly, neurons significantly coding each of the canonical variables could be identified in each animal with similar proportions (Figure S5E). Single units also represented the change in effort at effort block transitions, although this was considerably less frequent than trial-by-trial effort coding (Figure S5F). Units in each area also encoded a wide variety of other task and behavioral parameters with variable degrees of overlap with the units encoding canonical task variables (Tables S1, S2, S3, and S4); however, including these in our regressions did not fundamentally change the proportional makeup of neurons coding for canonical decision variables. Strikingly, reward and choice tuning was more frequent in the DMS and STA compared to other structures (Figure 2F, see legend for statistical comparisons). Moreover, the co-representation of decision parameters within single neurons was also more common in the DMS and STA compared to either of the frontal cortical regions (Figure 2F, see legend for statistical comparisons). Together, these data suggest that single neuronal representations are highly distributed.

### The dynamics of cost-benefit representations in cortex and basal ganglia are underpinned by diversity in temporal and directional properties

**Figure 3:**
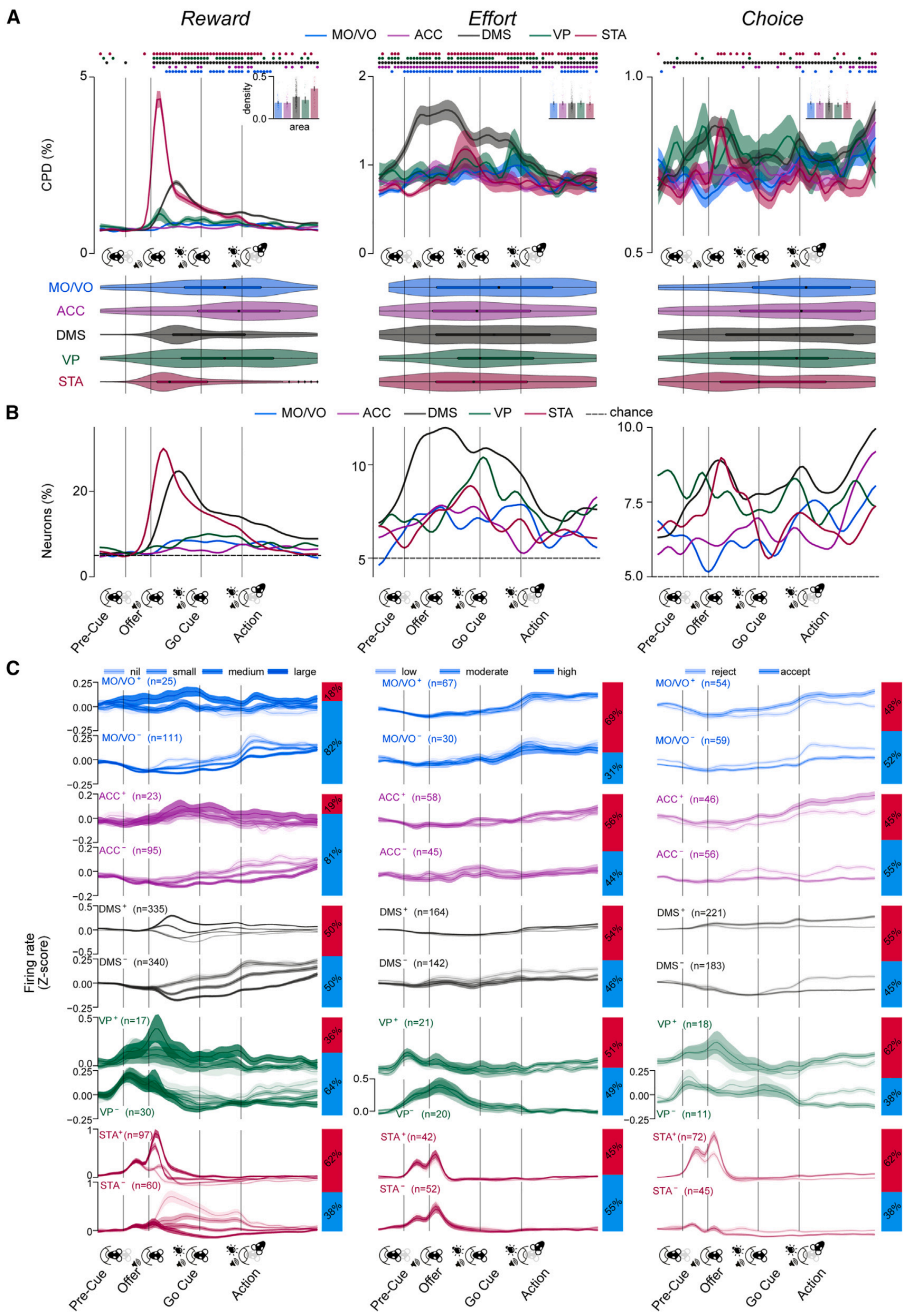
Dynamic and distributed single neuronal representations of reward, effort, and choice across the frontal-basal ganglia network (A) Top: population average CPD time series, depicted as means (solid lines) ± SEM (shaded areas) across all units, for each decision variable (columns). Color-coded segments on the top indicate time points where the population average CPD was significantly higher than expected by chance in each region using a significance threshold of *p* < 0.05 (permutation testing, false discovery rate [FDR] corrected across time points). Insets depict individual unit (colored dots) and population average (bars) ± SEM phasicness scores for each area. Density comparisons for reward (top, insets): STA vs. all other regions: all |*Z*| > 4.34; *p* < 0.001, two-sided Mann-Whitney U test. DMS vs. all except VP: |*Z*| > 6.63 *p* < 0.001. All other |*Z*| < 1.84, *p* > 0.050. Bottom: latencies to peak CPD depicted as kernel density estimates (violins) and superimposed boxplots. Peak CPD latency comparisons for choice: STA vs. MO/VO: z = 2.85, *p* = 0.004; all other: |*Z*| < 2.67, *p* > 0.05. Instances of peak reward CPDs occurring before offer cue onset were detected at a below-chance rate (<3.72%, *p* > 0.05, one-sided binomial test). (B) Instantaneous recruitment of neurons dedicated to signaling any decision variable with a significance cutoff of *p* < 0.05. (C) Population-averaged *Z* scored firing rates (line graphs) of significant positively or negatively coding neurons (STAR Methods) grouped by positive (above, e.g. increasing firing rate with increasing effort) or negative (below, e.g. decreasing firing rate with decreasing effort) tuning valence and plotted on each level of a decision variable (dark lines) ± SEM (shaded areas). Proportions of neurons encoding each decision variable positively vs. negatively in each area are shown (right). Data in each group are averaged across other conditions. Curves in (A)–(C) are depicted over a warped trial timeline beginning 0.4 s before nose-poke entry up 0.9 s before the corridor entry time (STAR Methods) and smoothed with a Gaussian filter with a standard deviation of 1.0 bins.

Our finding that basal ganglia neurons were more specialized than those in the frontal cortex argues against models of cortico-basal ganglia circuits in which abstract decision variables (subjective reward value and effort cost) are computed in cortical regions and used to regulate action initiation and invigoration via basal ganglia structures.^22^^,^^35^ Nonetheless, such organization could be more evident over finer timescales. We thus analyzed the coefficients of partial determination (CPDs) with finer temporal resolution for each canonical decision variable, neuron, and area (Figure 3A; STAR Methods). To characterize the temporal properties of the CPD signal, we further analyzed (1) CPD “phasicness” and density—indices of the dispersion of encoding in each area’s single neuron population over time (STAR Methods)—and (2) latency to maximal CPD.

Prospective reward was significantly encoded from the onset of the reward offer cue until the decision point in all areas (Figure 3A, top left). Nonetheless, the reward signal in the DMS and STA units was phasic, peaking after presentation of the offer cue, while encoding in the other recorded areas was more dispersed (STA and DMS phasicness: all |*Z*| > 2.78, *p* < 0.001, all other |*Z*| < 0.98, *p* > 0.05; two-sided two-sample Kolmogorov-Smirnov test against a bootstrapped null distribution; Figure 3A, bottom). Peak reward CPD and recruitment of neurons was reached earlier in the STA compared to all other areas (Figures 3A and 3B, left; STA maximum CPD latency vs. other areas: |*Z*| > 3.43, *p* < 0.001) and earlier in the DMS than the cortical areas (both |*Z*| > 2.21, *p* < 0.027). Whereas the time course of reward coding was clearly linked to the onset of the offer cue, effort signaling was more sustained, being evident in the pre-cue and even the pre-trial period in most structures (all CPD phasicness tests: |*Z*| < 0.79, *p* > 0.050; Figure 3A, middle), with similar density (all |*Z*| < 0.85, *p* > 0.050) and peak CPD latency (Figure 3B, middle; all |*Z*| < 1.62, *p* > 0.050) in all. Choice, on the other hand, elicited phasic firing rate modulation in the DMS (Figure 3A, right; DMS CPD phasicness: |*Z*| = 1.89, *p* = 0.002; all other |*Z*| < 0.93, *p* > 0.050); however, density estimates did not differ between the recorded areas (all |*Z*| < 1.25, *p* > 0.050), and the peak CPD was weighted toward the go cue and action epochs in all regions (Figure 3B, right).

We conjectured that these phasic vs. distributed signals could be underpinned by groups of neurons that differ in their coding valence with respect to the decision parameters.^36^^,^^37^ As can be seen in Figure 3C, distributed reward coding in all areas appeared to be driven predominantly by *negatively* coding neurons, while phasic coding in the DMS and STA was mediated mostly by *positively* tuned single units (Figures 3C and S5–S7). In line with this, the maximum CPD latencies for reward were significantly longer in negative-coding DMS and STA neurons compared to positive ones (comparison of population-median maximum CPD latencies: both |*Z*| > 5.30, *p* < 0.001, Mann-Whitney U test). Such temporally disperse coding can be underpinned by either (1) persistent temporally disperse activity within single units or (2) tiling of task time by phasic signals of individual neurons.^38^^,^^39^ Examination of the phasicness of individual positively vs. negatively reward-coding units (Figure S5) revealed that the latter were significantly less phasic and thus more persistently active than the former in the DMS and STA (both |*Z*| > 3.08, *p* < 0.001, one-sided Mann-Whitney U test), while phasicness did not differ between these populations in other regions or for other task variables (all other |*Z*| < 2.67, *p* > 0.050).

Taken together, these data demonstrate that the temporal dynamics of rate-based encoding varied with respect to brain area, direction, and variable. Notably, positively encoding neurons in basal ganglia structures had particularly strong phasic encoding of prospective reward during the offer window, but the peak encoding of the majority of populations was spread across the entire task.

### Neurons across the cortico-basal ganglia network form cell assemblies during cost-benefit decision-making

Such diversity in the single neuronal firing rate readouts poses a challenge at the output interpretation level. How can levels of reward, effort, and intention to accept/reject these offers be inferred from such spatiotemporally varying inputs? One candidate mechanism of coordination across varying single neuronal signals is the formation of coactive assemblies, defined as groups of neurons that synchronize their activity in short time windows.^25^ We thus examined whether cell assemblies could be detected in the cortico-basal ganglia network during cost-benefit decision-making and, if so, whether they could encode decision-relevant variables through the coordination of diverse populations.

**Figure 4:**
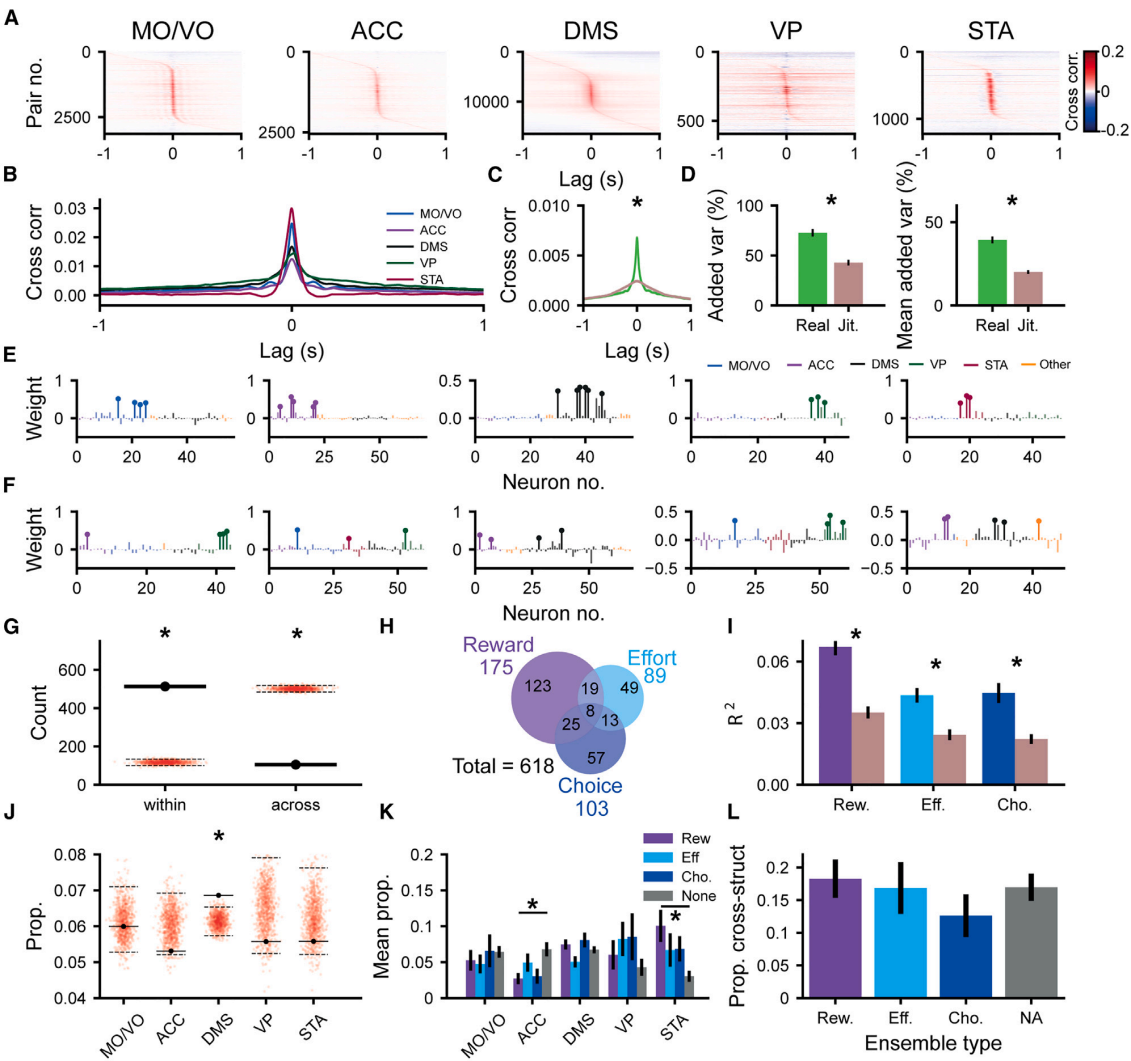
Co-firing assemblies across the frontal-basal ganglia network represent combinations of reward, effort, and decision (A) Heatmap showing the cross-correlation between pairs of units in the MO/VO, ACC, DMS, VP, and STA, sorted by the time of peak pairwise cross-correlation. (B) The mean cross-correlation between pairs of units in the MO/VO, ACC, DMS, VP, and STA. (C) The mean cross-correlation between pairs of units across all structures with (brown) or without (green) random jittering of spike trains in the range −250 to 250 ms (0 ms peak height, *p* < 10^−20^, Wilcoxon signed-rank test, n = 81,894 unit pairs). (D) The mean variance added over that of a single unit for the first eigenvector (left, *p* < 10^−12^, Wilcoxon signed-rank test, *n* = 68 PCAs) and mean variance added of the first 5 eigenvectors (right, *p* < 10^−12^, Wilcoxon signed-rank test, *n* = 68 PCAs) with or without random jittering of spike trains in the range −250 to 250 ms. (E) Lollipop plot showing example assembly patterns with members solely within the MO/VO, ACC, DMS, VP, and STA (left to right). Member neurons are marked with a circle on top of the bar indicating their weight. The color scheme is marked on top. Units identified histologically as outside of the 5 targeted brain regions are marked in orange. (F) Example assembly patterns that span multiple anatomical regions. Member neurons are marked with a circle on top of the bar indicating their weight. The color scheme is the same as that in (E). (G) The number of assembly patterns that span multiple anatomical structures (black marker) as compared to the proportion expected due to chance when shuffling the anatomical labels of neurons (red dots). The black dashed lines mark the 2.5^th^ and 97.5^th^ percentiles of this shuffled distribution. (H) Venn diagram showing the number of assemblies that have their coactivity significantly modulated by reward, effort, and decision outcome. (I) The variance in reward (R^2^ from simple linear regression, *p* < 10^−18^, Wilcoxon signed-rank test), effort (R^2^ from simple linear regression, *p* < 10^−6^, Wilcoxon signed-rank test), or decision outcome (pseudo-R^2^ from logistic regression, *p* < 10^−11^, Wilcoxon signed-rank test) explained by assembly pattern expression strength with (brown) or without (blue) random jittering of spike trains in the range of −250 to 250 ms for significantly encoding assemblies. (J) The mean proportion of single units from different brain regions that were members of each assembly as compared to the mean proportion when anatomical labels were shuffled among neurons (red dots). The 2.5^th^ and 97.5^th^ percentiles for shuffled distributions are marked with dashed black lines. (K) The mean proportion of single units from different brain regions in assemblies separated by their encoding properties. The proportion of neurons from each anatomical structure in encoding assemblies was compared to assemblies that did not encode canonical variables (significance for *p* < 0.050 determined by permutation test, Benjamini-Hochberg procedure used to control for multiple comparisons). (L) The proportion of cross-structural assemblies by assembly encoding type (*p* > 0.05 for reward, effort, and decision vs. non-coding, z test). ^∗^*p* < 0.050. Rew., reward; Eff., effort; Cho., choice; NA, non-coding.

We first addressed whether such short-latency co-firing was a common feature of these brain areas during our task. Many pairs of units across all anatomical structures were highly cross-correlated within a short interval (±50 ms) around a 0 s lag (Figures 4A and 4B), consistent with a physiologically meaningful readout for downstream neurons.^25^ These short-latency correlations were dependent on the precise temporal relationship between spike trains, as randomly jittering the spike trains between −250 and 250 ms reduced the baseline to peak difference in cross-correlation by 71% (Figure 4C). We used principal-component analysis (PCA) to determine the extent of spike timing coordination across groups of simultaneously recorded cells. The added variance explained by the first principal component was much larger for the unjittered *Z* scored binned spike train (Figure 4D, left), as was the mean of the variance added by the first 5 principal components (Figure 4D, right). This indicates that a significant proportion of neural activity in the frontal cortical-basal ganglia network is coordinated at the fine temporal scale necessary for the formation and expression of cell assemblies.^25^^,^^28^^,^^30^^,^^40^

We thus sought to identify assemblies of neurons that had a statistical tendency to coactivate, both within and across brain structures. To this end, we used PCA followed by independent component analysis (PCA-ICA) on the binned spike trains of neurons from all recorded regions during a time window spanning the start of the offer cue to the end of the action window (STAR Methods^41^^,^^42^). For this analytical pipeline, the spiking of all neurons was binned at 25 ms and *Z* scored. PCA was then applied, and significant principal components were identified based on the size of the associated eigenvalue (a readout of the variance explained by the principal component). ICA was then used to fine-tune the significant assembly patterns from PCA, allowing the analysis to reflect higher-order correlations and removing the requirement for assembly patterns to be orthogonal from one another.^28^^,^^41^^,^^42^ This pipeline outputted a vector for each significant assembly pattern, which contained the weight of each neuron in the assembly. The higher a neuron’s weighting, the greater the contribution it makes to the assembly.

This approach yielded a total of 618 assemblies. Importantly, several significant assemblies could be detected in a single recording (median = 10 assemblies per recording) (Figure S8), suggesting that we were not simply detecting some global tendency for neurons to coactivate. Most had “member neurons” (i.e., neurons with a high weighting in the assembly pattern) restricted to a single brain region (∼83%; Figures 4E and 4G). The remaining assemblies had member neurons that spanned multiple structures (Figures 4F and 4G), including a small proportion (13/618) that were made up of member neurons spanning 3 or more brain regions (e.g., Figure 4F). The proportion of assemblies that had member units from within a single structure was significantly greater than chance (Figure 4G).

We then examined whether such assemblies provide a unified readout of canonical decision variables. To this end, the mean expression of the coactivity pattern was modeled as a weighted linear sum of reward, effort, and choice (STAR Methods). Strikingly, nearly half (48%) of all identified assemblies had a significant degree of selectivity for the prospective reward, current effort cost, and choice, either individually or conjointly (Figure 4H). Several distinct assemblies identified in a single recording session could be found encoding the same variable (e.g., Figure S8), suggesting that the coactivity that encodes canonical variables was not a global brain-wide phenomenon but instead resulted from the co-firing of distinct combinations of units. Moreover, assemblies devoted to encoding each of the canonical decision variables were found in each recorded animal (Figure S11). Jittering the spike trains (Figure S9) preserved the mean firing rates over trials and almost all rate coding within the window of analysis (Figure S10) but substantially disrupted coding at the level of the assembly (Figure 4I).

We next sought to determine if the coactivity and assembly encoding we observed here were a result of the locking of units to task events. In order to investigate this, for each neuron, we shuffled spike trains across trials (for further details, see Figure S9) of the same level of reward, effort, or choice outcome. This exactly preserves the firing rate of neurons over the window investigated while also controlling for any coactivity that arises from the synchronized locking of units to task events. Trial shuffling the spike trains (Figure S9) disrupted effort and choice—but not reward—coding (Figure S12), owing to the highly synchronized activity around the onset of the reward cue. In these cases where many neurons lock their spiking to an external event, the formation of assemblies cannot be dissociated from event-related firing. However, for both choice and effort, it is clear that assembly coding cannot be accounted for by firing driven by the task structure. Overall, assemblies thus provided a substrate of encoding that coordinates activity across populations of neurons but is independent of the underlying correlated changes of firing rate at task-relevant timescales.

To understand how each region contributed to assemblies, we examined the likelihood (i.e., counts normalized by the total number of neurons from each structure recorded in a day) of neurons from any structure-forming assemblies regardless of their underlying tuning properties. We found that DMS neurons were slightly more likely to be members of coactive assemblies (Figure 4J) than would be expected due to chance. We then examined whether neurons from each area were more likely to be members of assemblies coding for one of the canonical decision variables (blue/purple bars, Figure 4K) than assemblies that did not code for any of the decision variables (gray bars, Figure 4K). Though the membership of task-tuned assemblies was relatively even across regions, STA neurons were more than twice as likely to be members of reward-coding assemblies than those that did not encode any decision variable (Figure 4K). In contrast, ACC neurons were *less* than half as likely to be members of assemblies encoding reward than assemblies that did not encode any variable (Figure 4K). Cross-structural assemblies were not significantly more likely to encode any of the decision variables (Figure 4L).

Together, these results demonstrate that assemblies were formed by neurons in each structure across the entire network, with STA neurons in particular having an important role in the expression of reward-related coactivity.

### Assemblies that encode decision variables are comprised of individual neurons with diverse tuning properties

The above analyses demonstrate that both individual neurons *and* assemblies encode and integrate the canonical decision variables. One possibility, therefore, is that the assembly code is closely correlated to each individual neuron’s tuning selectivity. However, it is also technically feasible for the temporal code within an assembly to be distinct from the rate code exhibited by the members of that assembly.

**Figure 5:**
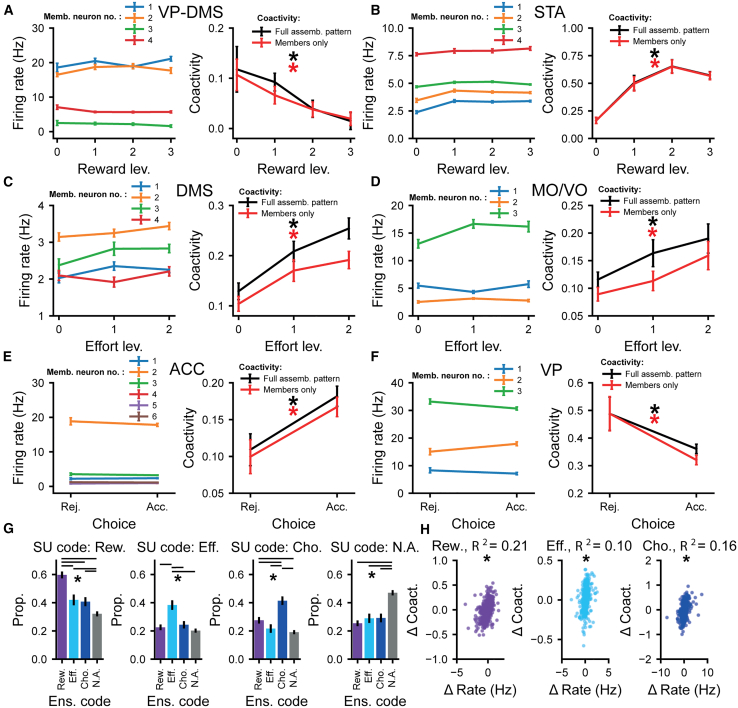
Decision-related encoding by co-firing assemblies can be orthogonal to the rate coding of the individual neurons that comprise them (A–F) Example assemblies that significantly code one of the canonical variables (right). All of the member neurons that make up these assemblies do not significantly rate code for the same canonical variable as the assembly as a whole (left, the firing rate of all of the individual member neurons that make up an assembly divided by levels of the canonical variable coded by the assembly as a whole). Firing rate and assembly pattern coactivity were calculated over the same window of time (the adjusted decision window; see STAR Methods). The single units that made up assemblies (left) are colored arbitrarily for display purposes. The anatomical structure an assembly is from is marked above it with text. (A and B) The firing rates of all the individual member neurons that make up example reward-coding assemblies (left). They show no significant change across reward levels. Assembly coactivity (right) was computed using the full assembly pattern weights (black) or restricting weights to only member neurons (red). Both the coactivity for the full assembly pattern and the coactivity patterns restricted to only member neurons are significantly correlated with the offered reward magnitude. (C and D) The firing rates of all the individual member neurons that make up example effort-coding assemblies (left). They show no significant change in firing rate across effort levels. Assembly coactivity (right) was computed using the full assembly pattern weights (black) or restricting weights to only member neurons (red). Both the coactivity for the full assembly pattern and the coactivity patterns restricted to only member neurons are significantly correlated with the level of effort. (E and F) The firing rates of all the individual member neurons that make up choice-coding assemblies (left). They show no significant change in firing rate with choice outcome. Assembly coactivity (right) was computed using the full assembly pattern weights (black) or restricting weights to only member neurons (red). The choice outcome has a significant effect on the coactivity pattern of both the full and members-only assembly patterns. For (A)–(F), ^∗^*p* < 0.050; significance was determined using linear regressions with firing rate or coactivity as the dependent variable and reward, effort, and choice as the independent variables. The CPD of reward, effort, and choice outcome was compared to the null distribution from trial shuffled data. The significance of the coding for both single units and assemblies is defined in the adjusted decision window. (G) The proportion of member neurons of reward, effort, choice, and non-encoding assemblies that rate code reward (left), effort (middle left), or choice outcome (middle right) or do not rate code for the canonical variables (right). All assemblies from across all animals and brain structures were pooled in this analysis. The significance of the coding for both single units and assemblies is defined in the adjusted decision window (see STAR Methods). ^∗^*p* < 0.050, z test, Benjamini-Hochberg procedure was used to control for multiple comparisons. (H) The change in the firing rates of member neurons across the levels of reward (left), effort (middle), and choice (right) outcomes only explains a small proportion (<22%) of the change in assembly expression strength (see STAR Methods). All of the assemblies from across all animals and brain structures were pooled in this analysis. The change in firing rate of single units and assembly coactivity was calculated over the adjusted decision window (see STAR Methods). ^∗^*p* < 0.050. Rew., reward; Eff., effort; Cho., choice; Rej., rejected trials; Acc., accepted trials. Reward levels 0, 1, 2, and 3 represent nil, low, medium, and high rewards, respectively. Effort levels 0, 1, and 2 represent the number of barriers in the linear track.

To address this, we asked whether ensembles that encoded a given decision variable were comprised of neurons that *rate coded* the same variable. Strikingly, we found examples of assemblies where the rate coding of individual neurons clearly diverged completely from that of the overall ensemble (Figures 5A–5F). To quantify the intersection of the encoding properties of single neurons (Figure 2F) and assemblies (Figure 4H), we defined the proportion of each type of rate-coding neuron that contributed to each type of encoding assembly (Figure 5G). Although single units that encoded a given variable were more likely to contribute to assemblies that encoded the same variable than any other assembly type (Figure 5G), between approximately 40% and 60% of the neurons that contributed to assemblies did not code for the same variable as the assembly as a whole (Figure 5G). Only a small proportion of the variance in the assembly pattern expression strength dynamics (<22%, for reward, effort, and choice) across levels of decision variables could be explained by changes in the firing rate of member neurons (Figure 5H), demonstrating that this bias was not simply a consequence of correlated, task-related firing rate across assembly members.

Taken together, these findings suggest that though a member neuron’s individual tuning often aligns with that of their assembly, assembly pattern expression strength changes were poorly explained by firing rate dynamics. Furthermore, neurons often contributed to assemblies encoding a different parameter to their own. Coactivity is thus separable from the rate code and can integrate units with diverse tuning properties.

### Encoding of reward, effort, and choice by cortico-basal ganglia assemblies aligns with relevant task events

Our analyses of assemblies so far defined encoding using expression strength across the entire choice window. If assemblies provide population-level encoding that has an ongoing influence on cost-benefit decisions akin to that proposed for single neurons, then their temporal dynamics should align with key task epochs.

**Figure 6:**
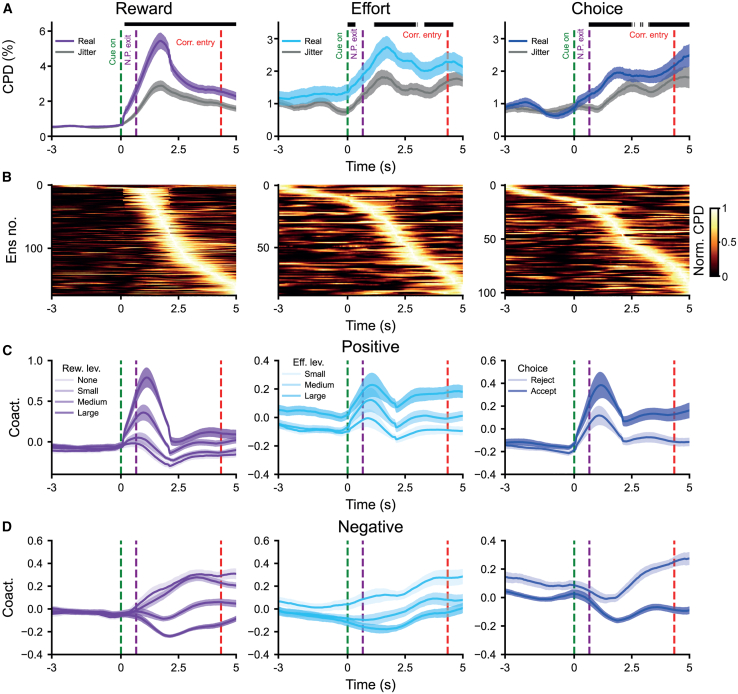
Co-firing assemblies can dynamically encode decision variables on task-relevant timescales This figure shows the coding of canonical variables across time pooling across assemblies from all brain regions. We specifically looked at all recorded assemblies that significantly coded reward (left), effort (middle), and choice (right) (as in Figure 4H). (A) CPD for reward, effort, and choice (left to right) regressed onto the time series of the expression strength of cell assemblies either with (gray) or without (blue/purple) the random jittering of the spike train in the range of −250 to 250 ms for all of the reward-, effort-, and choice-coding assemblies (left to right) (pooling assemblies across all brain structures and animals). A time of 0 s is the onset of the reward cue, and periods of time after cue onset where the CPD is significantly higher for the unjittered than the jittered assembly pattern expression strength are marked with a black bar (Wilcoxon signed-rank test, *p* < 0.050). The Benjamini-Hochberg procedure was used to control for multiple comparisons. There were no periods of significance prior to cue onset for reward-, effort-, or choice-coding assemblies (up to 3 s before cue onset, Wilcoxon signed-rank test, all *p* > 0.050, Benjamini-Hochberg procedure). (B) Heat plot where rows represent the normalized CPD for reward, effort, and choice regressed onto the time series of the expression strength of cell assemblies for each reward-, effort-, and choice-coding assembly, respectively (left to right) (pooling assemblies across all brain structures and animals). CPD was normalized by dividing by its peak value for each assembly, and assemblies were sorted by the time of peak encoding. A time of 0 s is the onset of the reward cue. (C and D) The *Z* scored, smoothed (using a Gaussian of SD = $2\;/\;\sqrt{12}\;s$) coactivity across the different levels of decision variables for positively (C) and negatively (D) reward-, effort-, and choice-encoding assemblies (left to right) (pooling assemblies across all brain structures and animals). Increasing levels of reward and effort and accept trials are shown in a darker color. A time of 0 s is the onset of the reward cue. Rew., reward; Eff., effort; Cue on, reward cue onset; N.P. exit, nose poke exit; Corr. entry, corridor entry.

To examine whether this was the case, we analyzed the time course of encoding (as measured by CPD) for significant *assemblies* with respect to the mean onset of the reward offer, action initiation, and nose-poke exit and subsequent accept/reject decision. Significance at a given time point was defined in relation to assembly coactivity constructed from jittered spike trains (Figure S9; STAR Methods). Reward-coding assemblies encoded at above-chance levels during virtually all time bins from the reward offer until the end of the decision point, whereas choice-encoding assemblies were largely significant from shortly after the nose-poke exit to corridor entry (Figures 6A and 6B). Effort encoding assemblies had patchier encoding that spanned from reward cue onset to corridor entry. As seen in the rate code of individual neurons, assemblies could encode positively (e.g., maximum expression strength for maximum reward) or negatively (e.g., maximum expression strength for minimum reward). Interestingly, the time course of assemblies that negatively encoded reward was more sustained/less phasic than for positively encoding assemblies (Figures 6C and 6D).

Taken together, this shows that assembly coactivity—reflecting temporally coordinated activations within and across frontal and basal ganglia structures—dynamically encodes reward, effort, and choice over timescales relevant to decision-making.

## Discussion

Cortico-basal ganglia circuits play a key role in choosing actions based on information about prospective costs and benefits, yet the mechanisms behind this have remained elusive. To gain a comprehensive understanding of potential encoding schemes, we developed an “accept-reject” decision paradigm inspired by diet selection foraging^43^ and previous effort-based decision-making paradigms.^44^^,^^45^ Single neurons, most prominently in the DMS and STA, though also in all other recorded regions, signaled information about the reward offer following cue presentation, as well as the current effort block and then the accept/reject decision. Nonetheless, a substantial fraction of all recorded neurons displayed a wide range of temporal activity patterns across the task space. Crucially, these same neurons formed cell assemblies, dynamically encoding the cost-benefit variables and decisions to act through synchronization of their spiking activity.

While there was an overrepresentation of reward-coding neurons in the DMS and STA, for the most part, encoding was anatomically and temporally distributed across the cortex and basal ganglia. At least 20%–30% of neurons in all five structures encoded *each* decision parameter, with neurons in each brain region having highly variable time courses for their coding. This temporally and spatially distributed code is consistent with that found in large-scale recordings with Neuropixel probes, where there is widespread neural activity across brain regions coding for variables such as action and task engagement.^46^ Our findings are consistent with a shift from a modular understanding, where each brain region has distinct coding properties, to a more distributed model of brain function.

Our behavioral results clearly demonstrate that both the current reward value and stored knowledge of the current effort requirement were used to determine the animal’s choice. Both neuroimaging^47^ and electrophysiological^36^^,^^48^ studies consistently implicate the MO, ACC, and striatum in signaling the net effort-discounted value of reward. Here, we found that multiplexed combinations of reward and effort were more prevalent in basal ganglia than cortical structures, particularly in the DMS and STA. Striatal neurons have previously been shown to integrate reward with other decision variables,^49^^,^^50^ but to the best of our knowledge, this is the first report of this extending to conjoint cost-benefit coding.

Having demonstrated that rate-based encoding is generally distributed across the network, we hypothesized that synchronized firing could provide a means through which neurons with diverse tuning properties could be coordinated. We demonstrate that neurons across all the recorded cortico-basal ganglia regions can form cell assemblies and that these assemblies can encode decision parameters. Cell assemblies have been mostly described in hippocampal neurons during spatial navigation^25^^,^^40^ and during the representation of contextual information^30^ but, to our knowledge, have rarely been described in cortico-basal ganglia networks and never for key cost-benefit decision variables.^27^^,^^51^ Here, we provide evidence that assemblies within and across the cortex, striatum, and other basal ganglia structures (although particularly in the DMS and STA) can encode the levels of prospective reward and effort over timescales relevant to the utilization of that information to regulate choice behavior.

Assemblies promote firing within the membrane time constant (10–30 ms) that is optimal for discharging downstream neurons/assemblies and strengthening synaptic weights between those neurons.^25^ Instead of acting as individual processing units, neurons in cell assemblies, both within and across structures, represent information cooperatively via their synchrony. This synchronous firing can produce a composite downstream effect that cannot be achieved by single neurons alone.^25^^,^^26^^,^^40^ These spatiotemporal interactions in the frontal cortex and basal ganglia could promote the integration of network-level connectivity akin to that in hippocampal networks, where the key property of the assembly is to simultaneously mobilize enough pyramidal neurons to discharge downstream neurons and promote synaptic plasticity.

Strikingly, assemblies that encoded a given decision variable were often comprised of single neurons coding for either a different parameter or none of the canonical variables at all (see the schematic representation in Figure S13). Equally, we demonstrate via various analytical controls that assembly activity does not trivially result from single-unit activity. This finding supports the idea that such assemblies are *emergent*: the activity of the population is more than a simple sum of the individual members, echoing previous findings in the hippocampus.^30^ This has important implications for future studies of decision-making in these circuits, as it demonstrates that neurons that do not have clear, temporally consistent rate-coding responses (Figures 2 and 3) can still play an important role at the population level (Figure S13). This view is not mutually exclusive to neurons of a specific type having a specialized role within the network, as striatal neurons appear to do here. Rather, we suggest that higher-level emergent activity constitutes a parallel level of organization that binds individual neurons with disparate rate-coding properties.

Our findings suggest that while individual neurons across the brain can represent decision variables through a spatially and temporally distributed rate code, they can also do so via a population representation formed of co-firing assembly members. These two coding schemas may increase the coding capacity of the brain by providing distinct channels for the representation of information and may support parallel computations within the same brain circuit.

### Limitations of the study

First, while we endeavored to restrict our analyses to a choice-invariant interval of the trial, we were not completely able to rule out the possibility that some unobserved behavioral differences (e.g., anticipatory licking, turning, nose poking) may have contributed to the observed canonical decision variable coding. However, even when controlling for behavioral variables such as velocity and nose-poke exit latency *post hoc* in our linear models, canonical decision-variable-coding tendencies in individual neuron firing rates were still identifiable.

Second, our trial-shuffling control showed that much of the coactivity that drives reward encoding cannot be distinguished from cue-locked neural activity. While this does not rule out a role for assemblies in the coding of reward, novel methods, including manipulations to prove causality, will be needed to prise this apart in future studies where widespread, short-latency, task-related firing is present.

Third, despite attempts to target the projections from the MO/VO and ACC to the parts of the recorded basal ganglia,^32^^,^^33^ we could not confirm that the neurons we recorded were monosynaptically connected across the 5 recorded brain regions. As such, it is possible that a less distributed and more modular coding schema could be identified if we had greater anatomical precision. It is, however, reassuring that cross-structural assemblies could consistently be identified, suggesting that the brain regions we recorded were functionally connected.

Fourth, as we only used male rats, our findings are limited in generalizability across genders.

## Resource availability

### Lead contact

Further information and requests for resources and reagents should be directed to and will be fulfilled by the lead contact, Andrew Sharott (andrew.sharott@bndu.ox.ac.uk).

### Materials availability

The study did not use any unique reagents or materials.

### Data and code availability

• Data availability: data will be made available at https://data.mrc.ox.ac.uk.
• Code availability: core analysis code will be made available as of the date of publication at https://data.mrc.ox.ac.uk.
• Other items: any additional information required to reanalyze the data reported in this paper is available from the lead contact upon request.

## Acknowledgments

We would like to thank T. Akam, M. Rothwell, and B. Micklem for methodological and technical assistance and D. Dupret for feedback during the project. This work was supported by The Clarendon Fund and Mary Somerville Clarendon Graduate Scholarship in conjunction with the Department of Experimental Psychology (award SFF1819_CB2_MSD_ 1196514 to O.H.), the Medical Research Council UK (awards MC_UU_00003/6 to A.S. and MR/X022080/1 to M.E.W., A.S., and S.G.M.), the Oxford National Institute for Healthcare Research (NIHR) Biomedical Research Centre to S.G.M., and the 10.13039/100010269Wellcome Trust (Sir Henry Wellcome Fellowship 209120/Z/17/Z to C.G.M. and 202831/Z/16/Z and 214314/Z/18/Z to M.E.W.).

## Author contributions

Conceptualization, O.H., I.G., C.G.M., S.G.M., M.E.W., and A.S.; technical development, O.H., R.T., T.D., and C.G.M.; investigation, O.H., B.P., and I.G.; analysis, O.H., B.P., and I.G.; resources and funding acquisition, A.S. and M.E.W.; writing – original draft, O.H., I.G., and A.S.; writing – reviewing & editing, O.H., I.G., B.P., R.T., C.G.M., H.C., S.G.M., M.E.W., and A.S.; supervision, A.S., M.E.W., S.G.M., H.C., and T.D.

## Declaration of interests

The authors declare no competing interests.

## STAR★Methods

### Key resources table

**Table:** 

REAGENT or RESOURCE	SOURCE	IDENTIFIER
**Deposited data**		

Spike timings	This paper	https://data.mrc.ox.ac.uk.
Behavioral timestamps	This paper	https://data.mrc.ox.ac.uk.

**Software and algorithms**		

signal from SciPy: convolve(), used to convolve spike trains with Gaussians	Virtanen et al., 2020^62^	https://docs.scipy.org/doc/scipy/reference/signal.html
stats from Scipy: wilcoxon() used for Wilcoxon signed-rank test and mannwhitneyu() used for the Mann-Whitney U test ks_2samp() used for the Kolmogorov-Smirnov test	Virtanen et al., 2020^62^	https://docs.scipy.org/doc/scipy/reference/stats.html
decomposition from scikit-learn: PCA() and FastICA(), for assembly analysis	Pedregosa et al., 2011^63^	https://scikit-learn.org/stable/api/sklearn.decomposition.html
statsmodels.api: OLS() for linear regression	Seabold et al., 2010^64^	https://www.statsmodels.org/v0.10.2/
statsmodels.discrete. discrete_model: Logit() for logistic regression	Seabold et al., 2010^64^	https://www.statsmodels.org/v0.10.2/
statsmodels.stats. weightstats: ztest() for z-testing	Seabold et al., 2010^64^	https://www.statsmodels.org/v0.10.2/
NumPy: Matrix/array operations, helper functions, histogram() used to bin spike trains, corrcoef() used to compute correlation matrices	Harris et al., 2020^65^	https://numpy.org
random from NumPy: permutation() for data shuffling, rand() used for adding random jittering to spike timing	Harris et al., 2020^65^	https://numpy.org/doc/stable/reference/random/index.html
PyTorch: GPU parallelisation of matrix operations	Paszke et al., 2019^66^	https://pytorch.org
pandas: DataFrame(), used for the storage and manipulation of data	McKinney, 2010^67^	https://pandas.pydata.org
matplotlib.pyplot: ax.scatter(), ax.plot(), ax.bar(), ax.errorbar(), ax.hist(), ax.fill_between(), ax.imshow() used for data visualisation	J.D. Hunter, 2007^68^	https://matplotlib.org/3.5.3/api/_as_gen/matplotlib.pyplot.html
matplotlib_venn: venn3() for generating Venn diagrams	Konstantin Tretyakov (no accompanying paper)	https://pypi.org/project/matplotlib-venn/
Pymer4: Pymer4.Lmer used to fit linear mixed effects' models in Python	Eshin Jolly 2018^69^	https://eshinjolly.com/pymer4/
Phy: Phy graphical user interface used for spike curation	Cyrille Rossant et al., 2016^70^	https://github.com/cortex-lab/phy
KiloSort2: KiloSort2 package used for spike sorting	Pachitariu et al., 2023^71^	https://github.com/jamesjun/Kilosort2
Custom code	This paper	https://data.mrc.ox.ac.uk.

### Experimental model and study participant details

All procedures were carried out in accordance with the UK Animals (Scientific Procedures) Act (1986) and its associated guidelines. We used 12 group-housed Lister Hooded naive male rats (Charles River, U.K.) weighing 300-390 g at the beginning of training. Rats were maintained on a 12-h light/dark cycle (lights on at 7 a.m.). During testing periods, rats were food restricted to a target weight range of 85–90% of their free-feeding weight. Water was available *ad libitum* in their home cages. Following implantation with multielectrode drives, animals were singly housed.

### Method details

#### Behavioral apparatus

The operant box was designed in Fusion360 (Autodesk) and consisted of two 17.6cm diameter custom-designed 3D-printed plastic semi-circular arenas, connected by a 130 cm-long Perspex corridor with walls on either side. The corridor had 6 equidistantly placed infra-red (IR) light-gates to register the rats' position and 3 equidistant slots for barriers to increase the travel cost between the ends of the corridor. Each arena housed a custom designed 3D-printed plastic nose-poke and reward port, positioned symmetrically on either side. Nose-poke and reward ports contained a infrared light-gate and a white LED house light. Reward ports were connected with 20-mg food pellet dispensers (Med-associates) fitted outside the walls of the arenas using medical-grade silicone tubing. Each reward port housed a moveable floor that was able to obstruct the port. The electronics were controlled by a Teensy 3.6 microcontroller that ran on custom-written firmware. The apparatus was lit using far-red LEDs to minimise visibility during training and testing.

#### Behavioral paradigm

Rats initiated trials by poking and holding in the nose-poke port of the starting arena ("pre-cue"). The starting arena was randomly allocated on the 1st trial. After a pre-cue period (0.3–0.4s), an auditory cue ("offer") was again presented from the distal end of the apparatus to signal the available reward at end of the track from where they initiated. The available reward was pseudo-randomly varied trial-by-trial (4 sizes: nil, low, mid, high, corresponding to zero, one, three or six 20mg sucrose pellets), signaled by a distinct auditory cue. The cue-reward associations were counter-balanced across subjects. Rats were required to maintain nose-poking for another 0.6–0.8s until the onset of a visual Go cue, signaled by a white light LED. Premature exiting of the nose-poke either during the pre-cue or offer period would elicit a 5-s error time-out, indicated by a flashing house-light in the nose-poke, followed by a 5s ITI. If rats exited prematurely during the auditory cue, the same cue would be repeated on the next nose-poke attempt. A grace period of up to 100ms during nose-poking - where nose-poke exits did not count toward an error - allowed for minor adjustments in body position. To accept the offer, rats needed to enter the corridor (cross a light-gate 24cm away from the arena) within a time window; otherwise, that offer was rejected. The cost of pursuing reward was pseudo-randomly varied across blocks of 17–23 trials (3 levels: low, moderate, high), implemented using varying numbers of barriers (0–2 or 1–3 in implanted or non-implanted animals, respectively) in the corridor. The barriers were 0.5 cm thick and were 12 cm and 18 cm tall at their lowest and highest points respectively, with a 45-degree slant mid-way between to generate a more effortful zigzag of running between the ends of the corridor.

#### Behavioral training

Rats were handled daily for a week prior to training. Training consisted of 5 steps in both versions of the task.

(1) Step 1 ("magazine training") consisted of rats being introduced into the apparatus with bedding from their home cages and receiving 20 rewards in each food dispenser, which they were free to consume in 20 min.
(2) In Step 2 ("cue-reward association training") - entry of the starting arena as detected by entry/presence in the arena of latest reward delivery would trigger one of 4 auditory cues, predicting the magnitude of the promised reward in the distal reward port. The offer cue was played until the animal made an entry into the distal reward port. Next, the reward port LED was activated for a 1s pre-reward delay, followed by delivery of the reward corresponding to offer cue. 2.5s after reward delivery, the moveable reward port floor was elevated in that same arena, resulting in the blocking of that port to indicate that reward would not be available there on the next trial. After the rats had reached a criterion of initiating 120 trials in £ 60 min, rats were taken to step 3.
(3) In step 3 ("nose-poke training"), in v1, rats had to learn to sustain a nose-poke with a jittered 1-1.2s length in order to elicit the offer cue. If rats sustained a 0.3–0.4s-long nose-poke ("pre-cue"), one of 4 auditory cues would be played. Importantly, rats were not free to leave until another 0.6–0.8s later ("offer") once a visual Go cue, signaled by a white light LED in the nose-poke port, had turned on. This was implemented to ensure a movement-free period following offer presentation for electrophysiological analyses. The pre-cue nose-poke requirement was incremented from 0.1s to 0.4s in increments of 25 ms after every successful trial. Once animals had learned to sustain successful pre-cue nose-pokes, the cue nose-poke requirement was incremented from 0.0s to 0.8s in steps of 20 ms after every successful trial. Once animals had learned to complete 120 trials with the full 1.2s nose-poke requirement, jitters of 0.1s ("pre-cue") and 0.2s ("offer") were introduced. To allow for small changes in body posture that may have caused the nose-poke sensor to stop registering an entry, a 0.1s grace period was introduced which did not result in a nose-poke error. Importantly, if a nose-poke was interrupted for more than 0.1s (the grace period) during the "offer" window, the same offer cue would be repeated during the next trial. This was not an intentional feature, but rather the side effect of using a pre-determined sequence of offers. Once rats had reached a criterion of initiating 120 trials within 60 min, they were taken on to step 4.
(4) In step 4 ("barrier training") rats needed to nose-poke to elicit offer cues as outlined previously, but now needed to additionally learn to scale barriers when traversing the corridor between the arenas. Barriers were added in one by one in blocks of 40 trials, starting from 1 barrier and going up to a maximum of 3 barriers. Once rats had successfully managed to complete 120 trials within 60 min, they were taken on to step 5.
(5) In step 5 ("final training for non-implanted animals") *r*ats were required to either accept or reject a distal reward offer available at the other side of the corridor (Figure 2). After the onset of the Go cue, to accept the offer, rats needed to enter the corridor within a 3s time window as determined by a light-gate placed near the border of the arena; otherwise, that offer is rejected. The effort cost which was fixed across blocks of ∼20 trials (3 levels: low – 1 barrier; moderate – 2 barriers; high – 3 barriers; Table 3). Upon corridor entry, they were allowed a further 5s to traverse the corridor and reach the distal reward port. To ensure that animals did not suffer an additional time cost by choosing to pursue an offer on accepted trials, we sought to match the duration of accepted and rejected trials. This was done by shortening the ITIs on ‘accept’ trials on each given recording day by the previous day’s cohort median reward magazine entry latency (the interval between Go cue onset and reward magazine entry on accepted trials). If animals chose to reject a reward-effort offer by not running across the linear track, they were able to initiate a new trial on the same side of the track.
(6) In step 5 ("post-surgery re-training"), the corridor entry and traverse times were increased to 15s and 10s respectively post-surgery to account for the additional constraints on implanted rats with a recording tether. Additionally, to avoid collisions between the implant and the barriers, the number of barriers per each level of effort was reduced by 1. Rats were considered ready for the recording experiment once they managed to complete 120 trials within 60 min.

#### Outcome devaluation experiment

Following standard testing, outcome devaluation was performed on a subset of rats (*n* = 6, first cohort) in a within-subjects regular Latin square design. In devaluation sessions, subjects were placed in a solitary "feeding" cage with *ad libitum* lab chow for 1h prior to the start of the testing session. Rats were placed in a solitary "control" cage for 1h on control sessions. The outcome devaluation lasted 3 days altogether, out of which days 1 and 3 were testing days and day 2 was a baseline training day.

#### Multielectrode drive

For single unit recordings we used a bespoke driveable multielectrode device weighing ∼20g. The implant was comprised of a lid which housed a 128-pin connector designed for Intan 64-channel head-stages (Intan Technology), a plastic corpus that housed the electrode movement mechanisms, glass tubing and a base. All components were assembled in house. For assembly, the elements of the corpus were first 3D printed and post-processed with UV radiation and propanol. Second, holes were drilled in the corpus to allow threading of electrode wires and the driving mechanisms. Third, driving support mechanisms were created using metal rods, plastic shuttles and screws. Fourth, glass tubing was introduced into the driving mechanisms as electrode supports. Fifth, strands of 50-μm diameter tungsten wire (California Fine Wire) were twisted and heated to create 4- and 6-strand electrodes (tetrodes and hextrodes, respectively). Sixth, these were threaded through the glass tubing and the rest of the implant corpus. Seventh, ground wire stubs (connected to ground wires on the animal during surgery, see below) were introduced into the implant using a dedicated set of stationary holes. Finally, the implant was sealed and the electrodes cut to length by hand.

Three of the subjects (gh001, gh009, gh010) reported here received an implant that targeted all 5 regions, whereas one subject (oh011) received a modified drive without the VP electrodes (Electrode table). Electrodes in the MO/VO, ACC, and VP were arranged as a row along one sagittal plane. DMS electrodes were as rows along two sagittal planes and STA electrodes were arranged in triangular fashion around a center point. The ML and AP positions of each electrode in the drive were planned around a centre-point co-ordinate obtained as an average between readings in two different versions of a commonly used rat brain atlas^54^^,^^55^ that visually offered the most exposure in each structure, while also avoiding the ventricles (Figure S2A). Relative electrode positions in the AP and ML dimensions were planned around centroid co-ordinates.

**Table:** **Electrode table |** Overview of the number of electrodes and channels in each subject and targeted recording area.

	Number of electrodes (channels) in targeted area				
–	MOFC	ACC	DMS	VP	STN
oh011	4 (24)	4 (16)	5 (30)	0 (0)	3 (18)
gh001	4 (24)	4 (16)	4 (24)	2 (12)	3 (18)
gh009	4 (24)	4 (16)	5 (30)	2 (12)	3 (18)
gh010	4 (24)	4 (16)	5 (30)	2 (12)	3 (18)

#### Implantation of multielectrode drives for recordings in behaving rats

Electrode implantations were performed in a subset of expert animals (*n* = 4; electrode table). Surgeries were performed under isoflurane anesthesia (5% for induction, 1.5–2.5% maintenance) and oxygen (2L/min). Subcutaneous local anesthetic (Marcaine, 2 mg/kg, 2.5 mg/mL) and a non-steroidal anti-inflammatory drug (Metacam, 1 mg/kg, 5 mg/mL) were administered at the beginning of each surgery. Post-operative opioid analgesia (Vetergersic, 0.3 mg/mL, 0.03 mg/kg) was administered subcutaneously for three consecutive post-operative days.

Craniotomies were created above each of the targeted areas and *dura mater* thoroughly removed from the visible brain surface. The drive was aligned on the animals' heads by using the centre-point of the STA electrode bundle as a reference. Next, the drive was lowered to target depth on a stereotaxic frame (David Kopf Instruments) based on the DV reading of the STA electrode bundle from brain surface. The implant was then secured to skull-attached screws using adhesive dental cement and dental acrylic. Recordings were referenced to two supra-cerebellar screws which were connected to the implant during surgery. Neuronal recordings began at least 2 weeks after surgery. Electrode placement was confirmed using histology.

### Electrophysiology data acquisition and processing

Wideband and accelerometer data were recorded, amplified and digitised on 2 tethered Intan 64-channel head-stages at 20,000 Hz using an Intan acquisition board (Intan Technologies). No hardware filtering was applied during recording. Files were saved in binary format. Each electrode was lowered by at least 125 μm up to 2h ahead of each recording session to ensure identification of unique units every day.

### Quantification and statistical analysis

#### Histological data analysis

To verify electrode placements, after the experiments were completed, animals were deeply anesthetized with isoflurane (5%) and intraperitoneal pentobarbital (3 mL, Pentoject, 200 mg/mL and perfused *trans*-cardially with phosphate buffered saline (PBS) followed by fixative solution (paraformaldehyde dissolved in PBS, 4%, wt/vol). For postmortem fixation, the sectioned heads were further submerged in fixative for another 24h, following which brains were extracted and washed 3 times in PBS and stored in a PBS solution containing 0.02% sodium azide until sectioning. Brains were sectioned at 0.1mm thickness using a vibratome (Leica), immunohistologically stained for FoxP2 and TH, and mounted in Vectashield. Unfiltered "bright-field" epifluorescence microscope images were acquired using 5× magnification and Zen Blue Pro (Zeiss). Histological results are reported in Figure S2–S3.

#### Electrode localisation and histological verification

To estimate all the possible recorded areas (Figure S2B) and exclude cells from neighboring regions, we (1) estimated each identifiable electrode tract’s DV end-depth from our epifluorescence imaging data, (2) averaged this across all identifiable electrode tracts to get an average end-depth, (3) estimated each day’s targeted structure based on a log of putative lowering distances averaged across electrodes within a region. We did not restrict the cell yield for the STA based on histological data. For other structures we restricted our cell yields to only the MO or VO for MO/VO, Cg1 and Cg2 for the ACC, CPu for the DMS; and VP only for the VP. Estimation of the position of the electrode tip on a given recording day was based on the estimated extent of electrode advancement (i.e., the number of turns of the screw driving the electrode within the implant) and the tract location in the histological data. Wherever neurons were detected on days where the anatomical localisation of the electrode tip suggested we were either out of the brain near a target structure or in a nearby white matter tract, but that electrode trajectory was consistent with being in the target areas, we presumed that the error was in the estimate of the electrode tip and these cells were included in the analyzed cell yield for that area. Neurons were not included if the entire electrode trajectory was not on target.

#### Spike sorting

Clustering and curation of units were carried out in KiloSort2^56^ and Phy (available at https://phy.readthedocs.io/en/latest/), respectively. Both are open-source libraries optimised for high-density multielectrode recordings. Individual units were isolated from the broadband signal based on waveforms using the KiloSort2 automatic clustering algorithm. A number of quality control steps were used in order to verify the single units identified by KiloSort2. Each neuron was manually inspected in Phy, and multiunits were excluded. Single units were first screened by the shape of the waveform. Only neurons with a stable waveform with spikes present across the entire recording block were included in further analysis. Next, we inspected the ISIs of each single unit to ensure that a clear refractory period was present. Cluster cutting was used to remove any contamination by electrical artifacts. Finally, the cross correlation between single units and their waveforms were used to merge those single units with a matching waveform and shared refractory period. Finally, single units with a firing rate less than <0.1Hz were excluded. No multi-units were used in the reported set of analyses. Numbers of isolated single units and their firing rate characteristics are reported in Figure 2 and Figure S2, respectively.

#### Behavioral data analysis

All data were analyzed in Python. Numbers of subjects and data depiction details are indicated in Results, Figure 1 and Figure S1. A binomial mixed effects model using the pymer4 library^57^ was used to regress the following fixed effects onto trial-by-trial choice.

• *reward*, coded as 0, 1, 3 or 6
• *effort*, coded as 1, 2, or 3 (0, 1, or 2 in implanted animals)
• *reward × effort interaction*, coded as the multiplication of those vectors
• *reward count*, coded as the number of pellets earned since the start of the session.

To safeguard against type I errors, we aimed to fit a maximal random effects structure whenever possible.^58^ To account for day-by-day and subject-by-subject covariation in the data, random slopes were paired with a random intercept for either the testing day or the subject ID. Whenever model fitting failed to converge, random effects that fit close to zero variance were removed to simplify the random effects' structure. The resulting decision model for the non-implanted cohort had the following formula:

accept = reward + effort + reward x effort + reward count + (reward | date) + (reward | subject)

while the implanted rat decision model was formulated as follows:

accept = reward + effort + reward x effort + reward count + (reward + effort + reward count| date) + (reward + effort + reward count | subject)

For the outcome devaluation experiment, we added pre-feeding and its interaction with reward and effort as an additional predictor, resulting in the following formula:

accept = reward + effort + pre-feeding + reward x effort + reward x pre-feeding + effort x pre-feeding + reward x effort x pre-feeding (1| date) + (1| subject)

All predictors were standardised to facilitate side-by-side comparison. *p*-values were obtained using the likelihood ratio test.

#### Firing rate modulation during task execution

For visualising task event-locked firing rates for representative neurons (Figure 2B), spiking counts were quantified in 10ms *non-overlapping* bins sampled in 10 ms increments −400ms to +400ms around the initiation of the nose-poke (termed "Pre-Cue"), −400ms to +400ms around the onset of the offer cue (termed "Offer"), −500ms to +500ms around the onset of the Go cue (termed "Go Cue") and −500ms pre-to +1000ms following nose-poke exit (termed "Action"). Data were subsequently averaged across trials of the same magnitude of offered reward, the required effort and prospective decision, each time averaging across the other two variables. Peri-stimulus time histogram (PSTH) data were smoothed with a Gaussian filter of standard deviation 5.0 bins for display purposes.

To facilitate alignment of firing rate data on the task (Figure 3C), time on trial was transformed into a uniform bin space by dividing the Pre-Cue epoch (−400 ms before Pre-Cue up to Offer) into 16 bins, the Offer epoch (from Offer up to Go Cue) into 16 bins, the Go Cue epoch (from Go Cue up to Action) into 13 bins and the Action epoch (from Action to 0.9s before observed corridor entry on accepted trials or 0.9s before the session median of corridor entry latencies on rejected trials) into 18 bins. For justification of the 0.9s cut-off, please see the section "Defining the decision window" below. Firing rates were quantified in 50 ms bins centered around each such bin and z-scored relative to a concatenated series of ITI data from the ongoing effort block.

#### Defining the decision window

To isolate a portion of the trial that was invariant to velocity changes between accepted and rejected trials, and thus representative of the decision process without movement confounds, we used DeepLabCut^59^ to extract rats' moment-to-moment head positions throughout videos recorded simultaneously to the electrophysiological data (Figure S4). We first examined the accuracy of position estimation in this dataset compared to the ground truth (light-gate activation). To do this, we visualised the DLC estimate of the rat head position during activation of one of four light-gates (left/right nose-poke or left/right reward magazine; Figure S4). We then calculated the percentage of DLC-estimated head positions that were within the true light-gate perimeter (figures S4C–S4F). Accuracy across all sessions in our electrophysiology dataset was 77–92% (figures S4D and S4F), with few instances of mal-positioned head locations scattered throughout the rest of the apparatus figures S4A–S4B). We then compared speed estimates obtained from our DeepLabCut data in 100ms bins ranging from −3 to +3s from corridor entry (as measured by triggered light gates) on accepted and rejected trials, respectively, and serially carried out one-way ANOVAs on each bin with choice specified as the independent variable. The earliest time bin of significance was identified by extracting the first Benjamini-Hochberg-corrected *p* < .05 in the obtained series of *p*-values. This revealed that the speed profiles meaningfully diverged 0.90s before corridor entry (main effect of choice, F_1,2_ = 8.52, *p* = .027), driven by higher average speeds on accepted trials (Figure S4G). This led us to establish a cut-off of 0.9s before corridor entry (adjusted corridor entry) as being the end of the decision window.

#### DeepLabCut video analyses

We carried out the above analysis on the positional data extracted from DeepLabCut. We trained the resnet_50 model on labeled frames from 664 videos (3 frames labeled per video) which spanned all four recorded animals across all configurations of barriers. Specifically, we labeled the position of the back of the head of animals, between their ears. The model was trained over 900,000 iterations until the loss had plateaued. Model performance was then checked by visual inspection of labeled videos and using the alignment with light gate data (see Figure S4).

#### Individual neuron regression analyses

General linear models (GLMs) were employed to quantify the influence of reward, effort and prospective decision on z-scored individual unit firing rates. Only single units whose firing rates in any given time bin of analysis was less than or equal to 0.1Hz were excluded. The loading of each variable on z-scored firing rates were obtained using the closed-form least-squares solution:

$$b={\left(X^{T}\;X\right)}^{-1}\;X^{T}\;Y$$

where X is a design matrix of shape [trials x variables] and Y is a vector of z-scored firing rates of shape [trials x 1] from one recording day. The 4 regressors used in the design matrix X were constructed as follows.

• Intercept: a vector of ones.
• Reward: a vector containing the values 1, 3 or 6 corresponding to small, medium or large reward trials.
• Effort: a vector containing the values 1, 2 or 3 corresponding to low, moderate or high effort trials.
• Decision: a vector comprised of the values −0.5 and +0.5 corresponding to rejected or accepted trials.

All regressors except the intercept were z-scored to themselves before fitting the models and obtaining coefficient loadings. From these GLMs, coefficients of partial determination (CPD), reflecting the unique variance explained by a variable, were obtained as follows:

$${\text{CPD}}_{i}=100\times \left(\frac{{\text{SSE}}_{∼i}-{\text{SSE}}_{i}}{{\text{SSE}}_{∼i}}\right)$$

where SSE_∼i_ and SSE_i_ represent the sum of squared errors of models that, respectively, do not or do include the regressor of interest. Using CPD ensured that the encoding strength for a given variable was not secondary to another (e.g., higher firing rate/encoding on accept trials due to more of these trials having higher prospective reward). To identify significant coding, permutation testing was used. For each neuron and decision variable, 1,000 shuffled datasets were generated by randomly shuffling the trial indices within each recording day. Individual neuron regression coefficient *p*-values were calculated as the fraction of (1,000) permuted CPD values which exceeded a neuron’s non-permuted CPD.

Significant neurons (Figures 2F, 3B, 3C, and S5–S7) were identified on the basis of CPD *p*-values less than 0.05 in *one single time bin* abutting the onset of the offer cue to 0.9s before observed corridor entry on accepted trials or the session median thereof on rejected trials. Individual neuron examples in Figure 2B were selected if they met this criterion and displayed some consistency in the discrimination response over time based on visual assessment.

To elucidate fine temporal dynamics contributing to encoding reported Figure 2F, regression results (Figures 3A and 3B) and firing rates (Figures 3C and S5–S7) were displayed as a time series of overlapping 50 ms time bins. The centre-points of those bins were obtained by dividing the Pre-Cue epoch (−400 ms before Pre-Cue up to Offer) into 16 bins, the Offer epoch (from Offer up to Go Cue) into 16 bins, the Go Cue epoch (from Go Cue up to Action) into 13 bins and the Action epoch (from Action to 0.9s before observed corridor entry on accepted trials or the session median thereof on rejected trials) into 18 bins. For justification of the 0.9s cut-off, please see the section "*Defining the decision window" above*. Regression models (Figures 3A, 3B, and S5–S7) were fitted on each resultant bin. The firing rate time series in Figure 3C were z-scored relative to a concatenated series of ITI data from the ongoing effort block.

Instantaneous population CPD *p*-values (Figure 3A) were calculated as the fraction of permutations for which the (1,000) shuffled population average CPDs were larger than the non-permuted population mean CPD. The Benjamini-Hochberg (BH) procedure was applied to correct for false discovery rate across tested time points. The proportions of significantly coding neurons between regions were compared pairwise between areas using the z-test of proportions with BH correction for the number of unique comparisons.

#### Quantification of CPD density (Figure 3A insets)

To characterise the degree to which individual unit CPDs magnitudes were temporally localised vs. distributed across the trial timeline, we calculated *density.* This is related to *sparsity* as defined by previous studies^28^^,^^60^ and indexed the extent to which the CPD time series for a population of neurons was dominated by a small number of time points. For each neuron in each area, it was defined as follows:

$$\frac{\surd n-\sum \left|v_{i}\right|}{\surd n-1}$$

where n is the length of the CPD vector (i.e., the number of time points) and v_i_ is the CPD vector’s i^th^ element. For this analysis, the input CPD time series was a unit vector where the sum of the squares of all elements added up to one. Density distributions were compared between brain areas using the two-sided Mann-Whitney U test with BH correction for the number of pairwise comparisons. All Mann-Whitney U test results reported in this work as expressed as a *Z* score obtained as:

$$z=\frac{U-\frac{n_{A}n_{B}}{2}}{\sqrt{\frac{n_{A}n_{B}\left(n_{A}{+n}_{B}+1\right)}{12}}}$$

where U is the Mann-Whitney U test statistic and n_A_ and n_B_ represent the sample sizes in the two comparison groups.

#### Quantification of latency to peak coding response and phasicness (Figure 3A, lower panel)

To quantify the latency to peak coding response, we calculated the time bin of maximal CPD magnitude for each neuron and plotted the distribution across all neurons as violin plots using the python Seaborn library. To evaluate whether these peak CPD latencies tended to co-occur close-by in time (phasic responding) more than expected by chance, we compared these values with a null-distribution formed by bootstrapped sampling of all observed unique latency values with replacement to match the original number of sampled neurons. The null and real distributions were compared using the two-sided two-sample Kolmogorov-Smirnov test and the false-discovery rate corrected for by using the BH correction. The z-scores of the obtained test statistics were calculated as follows:

$$z=\sqrt{\frac{n^{2}}{2}\cdot D}$$

where n represents the size of both the original and bootstrapped distribution and D represents the two-sample Kolmogorov-Smirnov test statistic.

#### Quantification of single unit firing rate encoding of effort change (Figure S5F)

To investigate whether there were such transitions in the neural activity, we visualised the z-scored firing rates on the first five trials of any given block and organised them by the magnitude of the effort change (Figure S5E). We then calculated the Pearson’s correlations between the firing rates and effort delta scores on a unit-by-unit basis and identified significantly coding as those whose correlations had a *p*-value <.05.

#### Co-activity analysis

Assembly patterns were determined using the combination of principal component analysis and independent component analysis (PCA-ICA) as detailed previously.^41^^,^^42^ This analysis was conducted using neurons from all brain structures simultaneously over 68 recordings across the 4 implanted rats. This allowed for the unsupervised extraction of assembly patterns which could be contained entirely within an anatomical structure or could span multiple brain regions. Spike trains were taken from full trials (on a trial-by-trial basis) on the linear track after cue onset. That is, for accepted trials spike trains were taken from cue onset to 2s after reward delivery. On rejected trials, where the animal did not enter the corridor, spike trains were taken from cue onset to cue offset when the window in which rats could enter the corridor and receive a reward had elapsed. The spike trains for all units during time periods specified above were binned at 25ms, z-scored and concatenated into an *n* x *B* matrix, where *n* was the number of neurons and *B* was the number of 25ms bins. The element of this matrix at position (i, b) was the z-scored spike count of neuron i in bin b.

PCA was then applied to the z-scored spike count matrix. The number of significant patterns embedded in the z-scored spike counts was determined using the Marcenko-Pastur law. This is an analytical result from random matrix theory, which bounds the magnitude of the eigenvalues from PCA that can occur due to chance. Principal components with eigenvalues above the upper limit of the bound set by the Marcenko-Pastur law were classified as significant. Principal components with an eigenvalue greater than this threshold captured more of the correlation which existed between the binned firing of neurons than should occur if all neurons spiked independently of one and other.^28^

The z-scored spike count matrix was then projected onto the significant principal components and ICA was applied to the resulting matrix. The output of ICA was an unmixing matrix. This unmixing matrix was then projected back onto the original basis, where each dimension represents an individual neuron. The columns of the resulting matrix represent assembly patterns. These assembly patterns contain a weight for each neuron. The greater this weight is, the greater the contribution the neuron makes to the identified coactivity pattern.^28^

There are several advantages to using the combination of PCA and ICA as opposed to using either technique alone.^28^^,^^41^^,^^42^ For instance, PCA has been found to group several assembly patterns into the first principal component. PCA also artificially constrains identified assembly patterns to be orthogonal to each other, meaning that a high weighting of a neuron in one assembly pattern limits the weighting of that neuron is subsequently identified patterns. Finally, PCA is only based on pairwise correlations and cannot identify higher order correlations. However, if ICA was applied directly to the z-scored binned spike count matrix, one could identify the same number of assembly patterns as there are neurons. This could result in the extraction of spurious patterns. To address these caveats, ICA is applied to the z-scored binned spike count matrix following dimensionality reduction using PCA. For an overview of the total number of assemblies, including their partition by canonical decision variable coding, please refer to Figure 4.

In order to determine the expression of assembly patterns across time, the outer product of each assembly pattern was determined.^28^The expression strength (R) of assembly pattern k can be determined as follows:

$$R_{k}\left(t\right)={z\left(t\right)}^{T}P_{k}z\left(t\right)$$

where P_k_ is the outer product of assembly pattern k with elements in the main diagonal set to 0, and z(t) is a column vector containing the smoothed z-scored firing rate of neurons at time t. The main diagonal of $P_{k}$ must be set to zero to ensure that assembly pattern expression strength cannot be driven solely by isolated increases in the firing rate of individual units. The instantaneous firing rate of neurons in z(t) were computed by convolving the spike train of each neuron with a Gaussian kernel and then z-scoring the resulting timeseries for each neuron. The standard deviation of the Gaussian kernel was set to 25/$\sqrt{12}$ ms to match the 25ms bin used for PCA-ICA.^61^ Convolution with a Gaussian kernel allows for greater temporal resolution when compared to the 25ms bins which were used to define the assembly patterns via PCA-ICA.

Linear and logistic regression was conducted using either the python module statsmodel or in custom written pytorch functions. The general strategy utilized in the linear regressions was to take an epoch in each trial, determine the value of variables such as the spike rate or the mean coactivity in this epoch and use this to predict reward, effort or choice outcome across trials, or vice versa.

For identifying coding ensembles (as in single units), the mean expression strength of an ensemble was calculated from an interval starting with offer cue onset and ending either 0.9s before corridor entry (adjusted corridor entry) or, in instances where the animal did not enter the corridor, the median duration taken for corridor entry on that recording session less 0.9s (see above, in Defining the decision window). This wide bin was necessary because co-incident spiking in shorter time windows was a relatively rare phenomenon and would have precluded meaningful identification of co-active ensembles. The mean expression strength was then fitted as the dependent variable in a linear regression with reward, effort and choice outcome as independent regressors. Significance was determined by comparing the CPD of reward, effort and choice to that of the 95th percentile of the null distribution where reward and effort levels or choice outcomes were shuffled across trials. After identifying ensembles with significant modulation by the level of reward, effort or choice, we probed the time course of this modulation. This was achieved by fitting regression models with reward, effort and choice outcome as independent variables to the smoothed assembly pattern expression strength at different time points. Smoothing was achieved by convolution with a Gaussian with a standard deviation of $2s\;/\;\sqrt{12}$ and a duration of 2s. The convolved signal was aligned in time with task events such that only coactivity preceding a given time point was available to models. The CPD for reward, effort and choice was compared to that of linear models fitted to the assembly pattern expression strength in jittered spike trains.

The expression strength of assembly patterns was calculated again as part of 2 analytical controls (see Figure S9 for more details).

• where the spike trains were jittered randomly in the range of −250ms–250ms.
• where the spike train of each neuron was randomly shuffled across trials with either the same level of reward, effort or choice outcome.

Simple linear regression or logistic regression with decision variables (i.e., reward and effort level and choice outcome) as the dependent variable and mean assembly coactivity over the decision window as the independent variable was used to evaluate the ability of assembly activity to predict decision variables via R^2^ (linear regression-for reward and effort level) or McFadden’s pseudo-R^2^ (logistic regression-for choice outcome).

We tested the relationship between the coactivity change across levels of canonical variables and related this to the change in firing rate of member neurons across the levels of the same variable. We wanted to exclude the possibility that increases in coactivity were trivially a result of increases in the firing rate of member neurons. An example of this would be an assembly that’s coactivity increases with increasing reward driven entirely by more spiking across member neurons as the reward increased. Using the same window described above, for each assembly we modeled coactivity using multiple linear regression with reward, effort and choice as independent variables. We then stored the beta values from multiple linear regressions, representing the change in coactivity that occurs after increasing each of the canonical variables by one level. In the instance of choice, the beta coefficient represents the change in coactivity from a reject to accepted trial. We carried out the same procedure for each firing rate of each of the member neurons of each assembly. That is, we modeled the firing rate of these neurons using multiple linear regression with reward, effort and choice as independent variables. Again, we stored the beta variables from the linear regressions, representing the change in firing rate that occurs with an increase in each of the canonical variables by one level. For choice, the beta variable represents the change in firing rate from a rejected to an accepted trial. We then asked how much variance in the change in coactivity across levels of a canonical variable (using the coactivity-variable beta for each assembly) could be explained by the mean change in firing rate across member neurons (the mean of the firing rate-variable beta values across all of the member units of the assembly).

### Additional resources

Data was analyzed using the following open-source python packages/functions listed below (key resources table).

## Supplemental information

Document S1. Figures S1–S13

Table S1. Direction encoding during the decision window We regressed reward, effort, and side of the available reward (left/right) on neuronal firing rates on accepted trials during the original decision window used in fig. 2f. Neurons encoding the side of the reward were ubiquitous across the targeted frontal-basal ganglia circuit (all proportions >26%, all p < .05, one-sided binomial test against a chance rate of 5% with Benjamini-Hochberg adjustment for multiple tests). To clarify whether including the side of the reward led to a reduction in the proportion of reward/effort coding neurons in the DMS and STA, we compared the prevalence of reward and effort coding neurons between this model and the original firing rate model (fig. 2f) in all areas. We found that the original proportions were preserved (all |z| < 1.17, p > .244, two-sided z-test of proportions) except for reward-encoding neurons in the STA (36.25% in the original vs. 29.44% in the current model: z = −2.08, p = .038, two-sided z-test of proportions)

Table S2. Nose-poke exit latency encoding between go cue onset up to nose-poke exit To examine coding of latencies to respond, we focused on encoding of nose-poke exit latencies in the interval between Go cue onset up to nose-poke exit (see latency distributions depicted in Fig. S1f). A significant proportion of neurons in all structures coded for this variable (all p < .05, one-sided binomial test against a chance rate of 5% with Benjamini-Hochberg adjustment for multiple tests). Including this variable in the regression models revealed largely preserved multiplexing tendencies compared to the original model (fig. 2f) with minimal overlap of units encoding nose-poke exit latencies and other variables (all multiplexing units <6%)

Table S3. Nose-poke error encoding during the offer window To examine premature nose-poke exit encoding (labeled cue nose-poke exit in this table), we focused exclusively on the Offer window (the interval between the onset of the offer cue and the Go cue) and asked whether the outcome of this interval– either a successful nose-poke or a premature nose-poke exit - was encoded in the firing rates of individual neurons, along with the other canonical decision variables. It is important to note that offer windows with successful nose-poke attempts were repeats of the same reward offer as encountered on any previous failed nose-poke attempt(s). Premature exits were coded by significant proportions of units in each area (all p < .029, one-sided binomial test against a chance rate of 5% with Benjamini-Hochberg adjustment for multiple tests). Compared to the original regression model (fig. 2f), this analysis demonstrated a decrease in the proportion of choice-coding units in all regions except the VP (|z| = 0.90, p > .050; all other |z| > 2.69, p < .006, z-test of proportions) - however it is important to note that there was relatively little choice coding during the offer window (fig. 3b). There was very little evidence of multiplexing of the two variables (all premature exit and choice multiplexing units <6%), suggesting that these behaviors have different underlying encoding patterns

Table S4. Run velocity encoding proportions To examine how many neurons encoded running speed, we extracted the averaged head movement velocities from our DeepLabCut-processed video dataset during the interval between nose-poke exit and either reward magazine entry on accepted trials or the end of the action period on rejected trials. As expected, the velocity distributions between the two trial types were meaningfully different (fig. S4e, t_8287.63_ = −168.95, p < .001, two-sample student t-test). Again, significant proportions of single units in all areas encoded both the canonical decision variables as well as velocity (all p < .001, one-sided binomial test against a chance rate of 5% with Benjamini-Hochberg adjustment for multiple tests). However, in this model reward encoding was less frequent than in the original model (|z| > 2.49, p < .012 in MO/VO, DMS and STA; |z| < 1.36, p > .050 in the ACC and VP), possibly owing increased temporal separation from the onset of the offer cue where coding was the strongest. By contrast, effort and choice coding was much more pronounced in this time interval (|z| > 2.06, p < .039 in all except the VP; VP: z = 1.66, p > .050). Velocity was encoded by more than 33% of units in each area, with variable but mostly small degrees of multiplexing (up to 9%) with the canonical decision variables

Document S2. Article plus supplemental information
