# Multi-level encoding of reward, effort and choice across the frontal cortex and basal ganglia during cost-benefit decision making

**DOI:** 10.1016/j.celrep.2024.115209

**Published:** 2025-01-22

**Authors:** Oliver Härmson, Isaac Grennan, Brook Perry, Robert Toth, Colin G. McNamara, Timothy Denison, Hayriye Cagnan, Sanjay G. Manohar, Mark E. Walton, Andrew Sharott

**Affiliations:** 1 Medical Research Council Brain Network Dynamics Unit, Nuffield Department of Clinical Neurosciences, University of Oxford, Oxford, United Kingdom; 2 Department of Experimental Psychology, University of Oxford, Oxford, United Kingdom; 3 Department of Engineering Science, University of Oxford, Oxford, OX1 3PJ, UK; 4 Nuffield Department of Clinical Neurosciences, University of Oxford, Oxford, United Kingdom; 5 Wellcome Centre for Integrative Neuroimaging, University of Oxford, Oxford, United Kingdom

**Keywords:** Cost-benefit decision-making, cortico-basal ganglia networks, single neurons, ensembles.

## Abstract

Adaptive value-guided decision-making requires weighing up the costs and benefits of pursuing an available opportunity. Though neurons across frontal cortical-basal ganglia circuits have been repeatedly shown to represent decision-related parameters, it is unclear whether and how this information is coordinated. To address this question, we performed large-scale single unit recordings simultaneously across 5 medial/orbital frontal and basal ganglia regions as rats decided whether to pursue varying reward payoffs available at different effort costs. Single neurons encoding combinations of decision variables (reward, effort and choice) were represented within all recorded regions. Co-active cell assemblies, ensembles of neurons that repeatedly co-activated within short time windows (<25ms), represented the same decision variables, despite the members often having diverse individual coding properties. Together, these findings demonstrate a multi-level encoding structure for cost-benefit computations, where individual neurons are coordinated into larger assemblies that can represent task variables independently of their constituent components.

## Introduction

Deciding whether to invest effort in the pursuit of potential rewards is a challenge that organisms continually face. A wide range of areas across the medial frontal cortical-basal ganglia circuit have been implicated in such cost-benefit decisions ^1–9^. Single-units within individual regions have been shown to code decision-relevant variables such as expected reward value and effort cost ^10–20^. However, we currently lack a mechanistic understanding of how activity across these frontal and basal ganglia regions account for how animals evaluate a beneficial opportunity and regulate whether actions are initiated to pursue that opportunity.

One potential reason is that while work to date has focused on how single neurons in individual regions encode key cost-benefit decision parameters, much less is known about how such coding is *coordinated* across an extended network of brain regions. A common proposition is that single neuron responses reflect and contribute to modular processing across specialised brain areas that arrive at a behavioural output through serial information transmission ^21–23^. In sensorimotor tasks, information from cortex is transferred reliably and topographically to connected areas of striatum ^24^. However, this need not be the case for cost-benefit calculations, where information presented over different timescales needs to be integrated and then utilised to regulate subsequent choice.

One mechanism to achieve such distributed processing is the formation and expression of co-firing assemblies, whereby groups of neurons repeatedly fire action potentials within a short-latency (10-30ms) of each other ^25,26^. These assemblies have been demonstrated to play a vital role in population-level representations in areas such as the hippocampus^27–29^. Short-timescale coincident spiking can effectively be read out by downstream neural populations on timescales that are optimal for inducing plasticity ^25,30^. Despite the potential of co-firing assemblies to co-ordinate neural activity underlying cost-benefit decision-making, it is not clear whether they are formed in cortico-basal ganglia circuits and, if so, whether they can encode relevant variables. Determining this requires simultaneous large-scale recording of neurons across the network during cost-benefit decision making, which hitherto has rarely been conducted.

To address these questions, we performed simultaneous recordings of single neurons across interconnected frontal and basal ganglia regions – anterior cingulate cortex (ACC), medial/ventral orbitofrontal cortex (MO/VO), dorsal medial striatum (DMS), ventral pallidum (VP) and subthalamic nucleus area (STA) – while rats evaluated whether to accept or reject cost-benefit offers. Individual neurons across the entire network encoded combinations of reward, effort and choice, with a particularly pronounced phasic and multiplexed signal in the DMS and STA. Importantly, co-firing assemblies were also detected within and across all cortico-basal ganglia sites encoding the three main decision parameters independently from the individual firing rates of the member neurons. Remarkably, these often included individual neurons whose firing rates signalled a parameter *different* to the tuning of the overall assembly. Co-firing assemblies thus coordinate individual neurons with wide-ranging coding properties and give rise to emergent representations that can mediate cost-benefit computations at the network level.

## Results

### Choice on a novel rat accept/reject cost-benefit choice paradigm is shaped by anticipated costs and benefits

**Figure 1:**
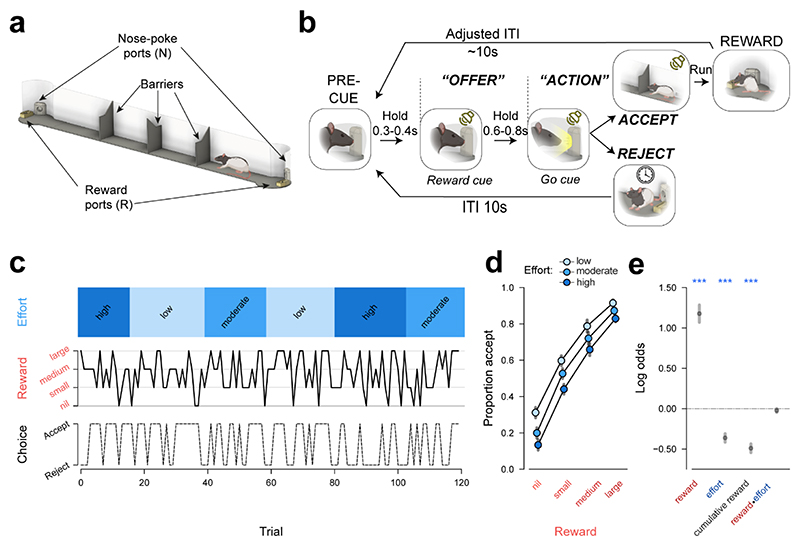
Choice on a novel rat accept/reject choice paradigm is shaped by the costs and benefits. **a**, Illustration of the experimental apparatus which consisted of a 130cm corridor with a variable number of barriers with circular arenas at either end, each of which contained a nose-poke port and a reward port. **b**, Rats chose between reward, signalled by an auditory cue during the “offer” window while the rat was in the nose-poke port, available at the far end of a linear corridor (’accept’) versus opting out (’reject’). To ensure average reward rate was minimally affected by rats’ accept/reject choices, ITIs were adjusted on ‘accept’ trials by subtracting the cohort median run time (STAR methods). Trials could always be initiated from the same arena where the last trial had finished (same arena on rejected trials; opposite side after an accepted trial). **c**, Reward/effort variation and choice in an example session. Reward magnitude (4 levels, middle panel) is signalled trial-by-trial by an auditory cue, whereas effort (3 levels, upper panel), is expressed in blocks as the number of barriers in the corridor. Choice is depicted as a dotted line on the bottom panel. **d**, Rates of accepting offers as a function of reward (x-axis) and effort (n=12). Coloured dots indicate mean ± SEM, adjusted for the within-subject variance across all levels of effort. **e**, weightings of task variables on behavioural choice from a binomial mixed effects model, depicted as mean ± SEM. Effect of reward x effort: β=-0.01 ± 0.02, p = .69. *** p < .001.

To elicit a trade-off between rewards and associated effort costs, we designed a novel cost-benefit task, inspired by foraging scenarios. Rats chose whether to pursue or forgo a reward available at the other end of a linear corridor based on its size and the set level of effort (Fig. 1a, STAR methods). Trials were initiated by poking and holding their noses in an initiation port (‘pre-cue’) while an auditory cue was presented (‘offer’), which signalled the size of the available reward on that trial (4 levels: nil, small, medium, large; Fig. 1b). Once the port light illuminated (‘Go cue’), rats could then leave the port (‘action’) and decide to either (i) pursue the offered reward by running to the opposite end of the corridor (‘accept’) or (ii) forgo the offered reward by refraining from entering the corridor (‘reject’). In both cases, trials were followed by an intertrial interval (ITI), after which a new trial could be initiated. The effort cost of pursuing the reward was manipulated by placing different numbers of barriers in the corridor, fixed across blocks of ~20 trials (3 levels: low, moderate, high; Fig 1c) in a pseudo-random order over the recording day. Reward offers were pseudo-randomly varied trial-by-trial but were repeated after a failed nose-poke attempt.

Rats’ choices were sensitive to both the benefits and costs of acting: animals were more likely to pursue reward when it was larger and when the effort cost was lower (n=12, non-implanted animals, Fig. 1c-e). This was reflected as a robust positive influence of reward (β=1.15 ± 0.10, p<.001, binomial mixed effects’ model) and a negative influence of effort (β=-0.33 ± 0.02, p<.001) over choice (Fig. 1e, STAR methods). Despite the lack of an explicit cue to signal the effort level, it nonetheless had a negative influence on decisions throughout trial blocks (Fig. S1a). Rats also progressively accepted fewer offers over the course of a session as they gained more rewards (β=-0.47 ± 0.02, p<.001), suggesting sensitivity to outcome value (Fig. 1e). This was corroborated by a follow-up experiment where rats (n=6) were given free access to lab chow for 1h prior to testing, which resulted in a significant reduction in willingness to accept any offer (Fig. S1b-c), indicating that choices on this task are not reflective of a fixed decision-making policy.

Sensitivity to reward and effort remained significant in a subset (n=4) of rats who were implanted for electrophysiological recordings in an adapted version of this paradigm (Fig. S1d-e, STAR methods). Reward offers also consistently influenced action initiation latency on successful trials (Fig. S1f) and premature nose-poke exit rates during the offer cue (Fig. S1j) in both sets of animals. Rejection of trials once already in the corridor only occurred at a rate of 6.81 ± 3.66% (between-animal median ± SEM) across all trials in implanted rats. Rats’ performance on this paradigm was thus robustly sensitive to the offered reward magnitude, effort cost and outcome value.

### Cost-benefit decision variable representations are asymmetrically distributed across the frontal cortical-basal ganglia network

**Figure 2:**
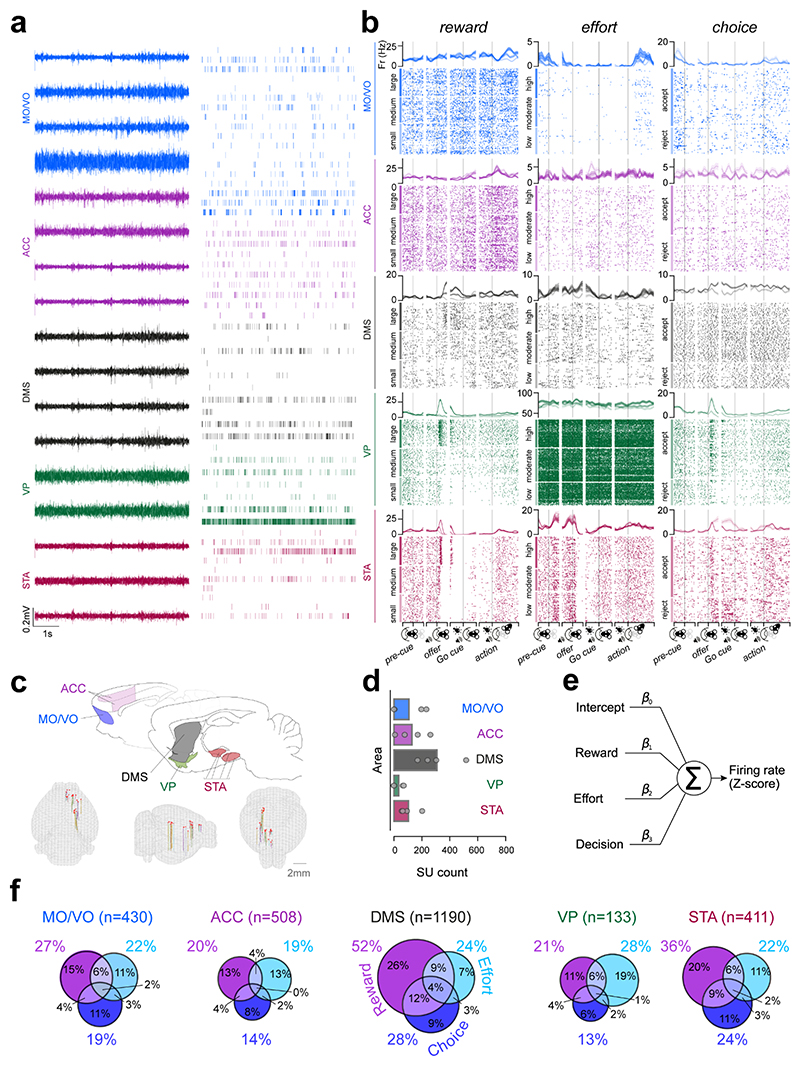
Single neurons encode combinations of reward, effort, and choice across the frontal cortex and basal ganglia. **a**, High-pass filtered signal (left) and individual neuron spiking times (right, STAR methods) displayed from an example five-site simultaneous recording from one example subject. **b**, peri-event time histograms (PSTHs, top panels) and raster plots (bottom panels) of 15 individual example neurons (one per panel) that monotonically and significantly varied their firing rate as a function of levels of reward, effort and the rats’ eventual choice (STAR methods). Data are time-locked to the beginning of the pre-cue, offer, Go cue and action epochs on the x axes, signified by vertical grey bars. PSTH data are displayed as averages across all other task conditions ± SEM (shaded areas). Data are displayed individually for each trial and grouped from top to bottom by levels of a decision variable, indicated on the left by coloured vertical bars. **c**, top: representations of targeted areas on sagittal sections adapted from ^52^; bottom: overview of trode locations on 3D meshes registered to the Allen Common Coordinate Framework 3D space using SharpTrack^53^, viewed from the back (left), right side (middle) and front (right). **d**, Overview of the average and per-subject single unit (SU) yield in each brain area. Means across subjects are depicted as bars and subject-by-subject cellular yields are marked as grey dots. We isolated on average 108 ± 107 (MO/VO, mean ± standard deviation), 129 ± 96 (ACC), 308 ± 130 (DMS) 34 ± 34 (VP) and 104 ± 58 (STA) single units per animal. **e**, Schematic of the multiple linear regression model used to analyse the contributions of decision variables on individual neuronal firing rates. **f**, Proportions of neurons that were tuned to canonical decision variables across a wide time window in each region and which had an average firing rate > 0.1Hz (STAR methods), detected based on significant coefficient of partial determination (CPD) values (p < .050). Pairwise comparisons for reward-coding neuron proportions: DMS vs all other structures: all |z| > 5.54, p < .001, two-sided z-test of proportions, adjusted for multiple comparisons using the BH procedure. STA vs all structures: all |z| > 2.89, p < .004. MO/VO vs ACC: |z| = 2.47, p = .015. All other |z| < 1.37, p > .050. Effort-coding neuron proportion comparisons: all |z| < 2.30, p > 050. Pairwise comparisons for choice-coding proportions: DMS vs all except STA: |z| > 3.62, p < .001; STA vs ACC and VP: both |z| > 2.81, p < .004.; all other |z| < 2.10, p > .050. Pairwise comparisons of the summed percentages of all multiplexing neuron types (intersecting Venn diagram areas) between regions: DMS vs all other regions: all |z| > 3.96, p < .001; STA vs MO/VO, ACC and VP: all |z| > 1.75, p < .040, one-sided z-test of proportions without BH correction. MO/VO = medial/vental orbitofrontal cortex, ACC = anterior cingulate cortex, DMS = dorsal medial striatum, VP = ventral pallidum, STA = subthalamic nucleus area.

Having established that choices on our task were concurrently sensitive to both reward and effort, we first aimed to identify neural correlates of the underlying computations at the level of single neurons. Given the role of the frontal-basal ganglia network in motivating cost-benefit choice^4,9,17,31^, we targeted the MO/VO, ACC, DMS, VP and STA (encompassing the subthalamic nucleus, zona incerta, substantia nigra pars reticulata and compacta) (Fig 2a-d, Fig. S2-3) using a bespoke driveable multielectrode implant to record individual neurons from well-trained rats (n=4) performing the cost-benefit task (STAR methods). The stereotaxic coordinates in the cortico-basal ganglia network were planned to maximise the possibility of recording from interconnected areas of the MO/VO, ACC and their downstream projections in the DMS^32^ and STA^33^ as well as VP - a key intermediary nucleus between regions of the striatum and the subthalamic nucleus^34^.

We first examined the propensity of cortical and/or basal ganglia neurons to encode single or multiple decision variables. We modelled each identified neuron’s z-scored firing rate as the weighted sum of offered reward, effort and subsequent accept/reject choice (Fig. 2e, STAR methods) during epochs containing the *entire decision process*. This interval started at offer cue onset and ended 0.9s before observed corridor entry on accepted trials or 0.9s before the median of corridor entry latencies on rejected trials (STAR methods; Fig. S4), allowing us to isolate a portion of the trial that was invariant to velocity changes between accepted and rejected trials. Significant proportions of units in each area linearly signalled each canonical decision variable (Fig. 2f, all p < .001, one-sided binomial test against a chance rate of 5% with Benjamini-Hochberg adjustment for multiple tests). Importantly, neurons significantly coding each of the canonical variables could be identified in each animal with similar proportions (Fig. S5e). Single units also represented the change in effort at effort block transitions, although this was considerably less frequent than trial-by-trial effort coding (fig. S5f). Units in each area also encoded a wide variety of other task and behavioural parameters with variable degrees of overlap with the units encoding canonical task variables (supplemental table 1-4), however including these in our regressions did not fundamentally change the proportional makeup of neurons coding for canonical decision variables. Strikingly, reward and choice tuning was more frequent in the DMS and STA compared to other structures, (Fig. 2f, see legend for statistical comparisons). Moreover, the co-representation of decision parameters within single neurons was also more common in DMS and STA compared to either of the frontal cortical regions (Fig. 2f, see legend for statistical comparisons). Together, these data suggest that single neuronal representations are highly distributed.

### The dynamics of cost-benefit representations in cortex and basal ganglia are underpinned by diversity in temporal and directional properties

**Figure 3:**
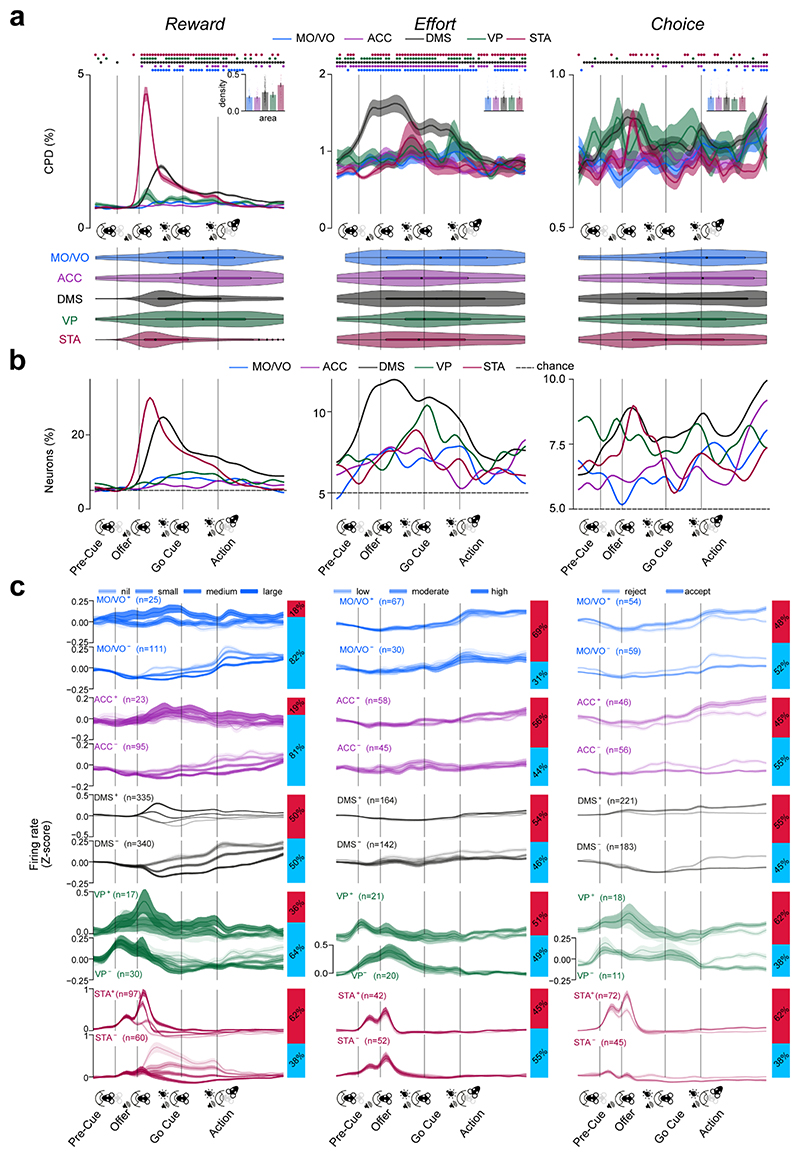
Dynamic and distributed single neuronal representations of reward, effort and choice across the frontal-basal ganglia network. **a**, upper, population average CPD time series, depicted as means (solid lines) ± SEM (shaded areas) across all units, for each decision variable (columns). Colour-coded segments above indicate time points where the population average CPD was significantly higher than expected by chance in each region using a significance threshold of p < .05 (permutation testing, fdr corrected across time points). Insets depict individual unit (coloured dots) and population average (bars) ± SEM phasicness scores for each area. Density comparisons for reward (fig. 3a upper, insets): STA vs all other regions: all |z| > 4.34; p < .001, two-sided Mann-Whitney U test. DMS vs all except VP: |z| > 6.63 p < .001. All other |z| < 1.84, p > .050. Lower, latencies to peak CPD, depicted as kernel density estimates (violins) and superimposed boxplots. Peak CPD latency comparisons for choice: STA vs MO/VO: z = 2.85, p = .004; all other: |z| < 2.67, p > .05. Instances of peak reward CPDs occurring before offer cue onset were detected at below-chance rate (< 3.72%, p > .05, one-sided binomial test). **b**, Instantaneous recruitment of neurons dedicated to signalling any decision variable with a significance cut-off of p < .05. **c**, Population-averaged z-scored firing rates (line graphs) of significant positively or negatively coding neurons (STAR methods), grouped by positive (above) or negative (below) tuning valence and plotted on each level of a decision variable (dark lines) ± SEM (shaded areas). Proportions of neurons encoding each decision variable positively vs negatively in each area (right). Data in each group are averaged across other conditions. Curves in a-c are depicted over a warped trial timeline beginning 0.4s before nose-poke entry up 0.9s before the corridor entry time (STAR methods) and are smoothed with a gaussian filter with a standard deviation of 1.0 bins.

Our finding that basal ganglia neurons were more specialised than those in the frontal cortex argues against models of cortico-basal ganglia circuits in which abstract decision variables (subjective reward value and effort cost) are computed in cortical regions and used to regulate action initiation and invigoration via basal ganglia structures^22,35^. Nonetheless, such organisation could be more evident over finer timescales. We thus analysed the coefficients of partial determination (CPD) with finer temporal resolution for each canonical decision variable, neuron, and area (Fig. 3a, STAR methods). To characterise the temporal properties of the CPD signal, we further analysed (1) CPD-“phasicness” and density - indices of the dispersion of encoding in each area’s single neuron population over time (STAR methods) - and (2) latency to maximal CPD.

Prospective reward was significantly encoded from the onset of the reward offer cue until the decision point in all areas (Fig. 3a, upper left). Nonetheless, the reward signal in the DMS and STA units was phasic, peaking after presentation of the offer cue, while encoding in the other recorded areas was more dispersed (STA and DMS phasicness: all |z| > 2.78, p < .001, all other |z| < 0.98, p > .05; two-sided two-sample Kolmogorov-Smirnov test against a bootstrapped null distribution; Fig. 3a, lower). Peak reward CPD and recruitment of neurons was reached earlier in the STA compared to all other areas (Fig. 3a, b left; STA maximum CPD latency vs other areas: |z| > 3.43, p < .001) and earlier in the DMS than the cortical areas (both |z| > 2.21, p < .027). Whereas the time course of reward coding was clearly linked to the onset of the offer cue, effort signalling was more sustained, being evident in the pre-cue and even pre-trial period in most structures (all CPD phasicness tests, |z| < 0.79, p > .050; Fig. 3a, middle), with similar density (all |z| < 0.85, p > .050) and peak CPD latency (Fig. 3b middle; all |z| < 1.62, p > .050) in all. Choice, on the other hand, elicited phasic firing rate modulation in the DMS (Fig. 3a, right; DMS CPD phasicness: |z| = 1.89, p = .002; all other |z| < 0.93, p > .050), however density estimates did not differ between the recorded areas (all |z| < 1.25, p > .050) and the peak CPD was weighted towards the Go cue and action epochs in all regions (Fig. 3b right).

We conjectured that these phasic vs. distributed signals could be underpinned by groups of neurons that differ in their coding valence with respect to the decision parameters^36,37^. As can be seen in Fig. 3c, distributed reward coding in all areas appeared to be driven predominantly by *negatively* coding neurons, while phasic coding in the DMS and STA was mediated mostly by *positively* tuned single units (Fig. 3c, Fig. S5-7). In line with this, the maximum CPD latencies for reward were significantly longer in negative-coding DMS and STA neurons compared to positive ones (comparison of population-median maximum cpd latencies: both |z| > 5.30, p < .001, Mann-Whitney U test). Such temporally disperse coding can be underpinned by either (1) persistent temporally disperse activity within single units or (2) tiling of task time by phasic signals of individual neurons^38,39^. Examination of the phasicness of individual positively vs. negatively reward-coding units (fig. S5) revealed that the latter were significantly less phasic and thus more persistently active than the former in the DMS and STA (both |z| > 3.08, p < .001, one-sided Mann-Whitney U test), while phasicness did not differ between these populations in other regions or for other task variables (all other |z| < 2.67, p > .050).

Taken together, these data demonstrate that the temporal dynamics of rate-based encoding varied with respect to brain area, direction and variable. Notably, positively encoding neurons in basal ganglia structures had particularly strong phasic encoding of prospective reward during the offer window, but the peak encoding of the majority of populations was spread across the entire task.

### Neurons across the cortico-basal ganglia network form cell assemblies during cost-benefit decision-making

Such diversity in the single neuronal firing rate read-outs poses a challenge at the output interpretation level. How can levels of reward, effort, and intention to accept/reject these offers be inferred from such spatiotemporally varying inputs? One candidate mechanism of coordination across varying single neuronal signals is the formation of *co-active assemblies*, defined as groups of neurons that synchronise their activity in short time windows^25^. We thus examined whether cell assemblies could be detected in the cortico-basal ganglia network during cost-benefit decision-making and, if so, whether they could encode decision-relevant variables through the coordination of diverse populations.

**Figure 4:**
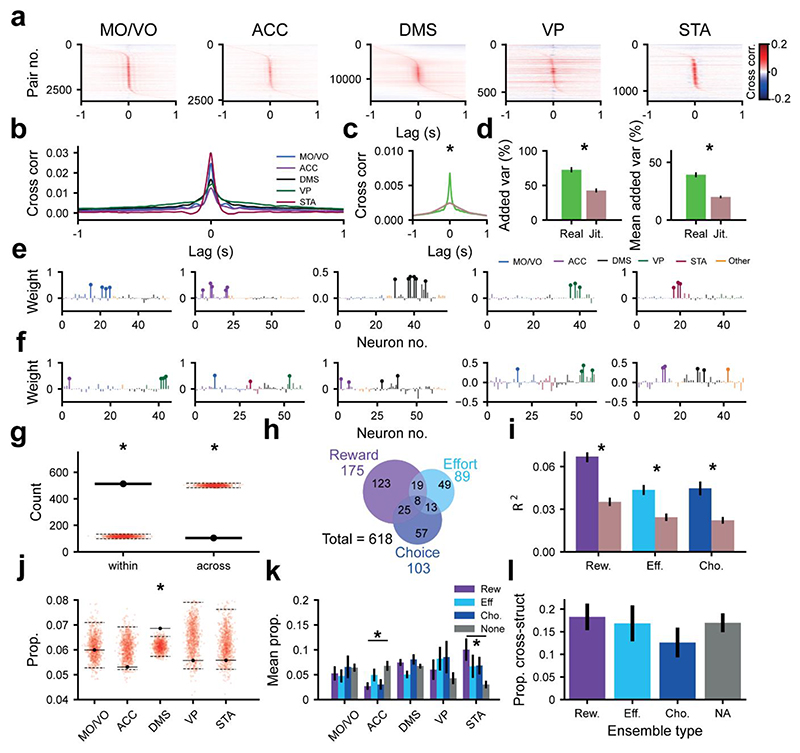
Co-firing assemblies across the frontal-basal ganglia network represent combinations of reward, effort and decision. **a**, heat map showing the cross correlation between pairs of units in the MO/VO, ACC, DMS, VP and STA, sorted by the time of peak pairwise cross-correlation. **b**, the mean cross correlation between pairs of units in the MO/VO, ACC, DMS, VP and STA. **c**, the mean cross correlation between pairs of units across all structures with (brown) or without (green) random jittering of spike trains in the range -250ms to 250ms (0ms peak height, p<10^-20^, Wilcoxon signed-rank test, n=81894 unit pairs). **d**, The mean variance added over that of a single unit for the first eigenvector (left, p<10^-12^, Wilcoxon signed-rank test, n=68 PCAs) and mean variance added of the first 5 eigenvectors **(**right, p<10^-12^, Wilcoxon signed-rank test n=68 PCAs) with or without random jittering of spike trains in the range -250ms to 250ms **e**, lollipop plot showing example assembly patterns with members solely within the MO/VO, ACC, DMS, VP and STA (left to right). Member neurons are marked with a circle on top of the bar indicating their weight. Color scheme marked above. Units identified histologically as outside of the 5 targeted brain regions were marked in orange. **f**, example assembly patterns that span multiple anatomical regions. Member neurons are marked with a circle on top of the bar indicating their weight. Color scheme as in **e. g**, the number of assembly patterns that span multiple anatomical structures (black marker) as compared to the proportion expected due to chance when shuffling the anatomical labels of neurons (red dots). The black dashed lines mark the 2.5^th^ and 97.5th percentile of this shuffled distribution. **h**, Venn diagram showing the number of assemblies which have their coactivity significantly modulated by reward, effort and decision outcome. **i**, the variance in reward (R^2^ from simple linear regression, p<10^-18^, Wilcoxon signed-rank test), effort (R^2^ from simple linear regression, p<10^-6^, Wilcoxon signed-rank test) or decision outcome (pseudo-R^2^ from logistic regression, p<10^-11^, Wilcoxon signed-rank test), explained by assembly pattern expression strength with (brown) or without (blue) random jittering of spike trains in the range of -250ms to 250ms for significantly encoding assemblies **j**, the mean proportion of single units from different brain regions which were members of each assembly as compared to the mean proportion when anatomical labels were shuffled amongst neurons (red dots). The 2.5^th^and 97.5^th^ percentiles for shuffled distributions were marked with dashed black lines. **k**, the mean proportion of single units from different brain regions in assemblies separated by their encoding properties. The proportion of neurons from each anatomical structure in encoding assemblies was compared to assemblies which did not encode canonical variables (significance for p < .050 determined by permutation test, Benjamini-Hochberg procedure used to control for multiple comparisons). **l**, the proportion of cross-structural assemblies by assembly encoding type (p>0.05 for reward, effort and decision vs non-coding, z-test). * p < .050. Rew. = reward, Eff. = effort, Cho.= choice, NA=non-coding.

We first addressed whether such short-latency co-firing was a common feature of these brain areas during our task. Many pairs of units across all anatomical structures were highly cross-correlated within a short interval (+/- 50 ms) around 0s lag (Fig. 4a-b), consistent with a physiologically meaningful readout for downstream neurons^25^. These short-latency correlations were dependent on the precise temporal relationship between spike trains, as randomly jittering the spike trains between -250ms to 250ms reduced the baseline to peak difference in cross correlation by 71% (Fig. 4c). We used principal component analysis (PCA) to determine the extent of spike timing coordination across groups of simultaneously recorded cells. The added variance explained by the first principal component was much larger for the unjittered z-scored binned spike train (Fig. 4d, left), as was the mean of the variance added by the first 5 principal components (Fig. 4d, right). This indicates that a significant proportion of neural activity in the frontal cortical-basal ganglia network is coordinated at the fine temporal scale necessary for the formation and expression of cell assemblies^25,28,30,40^.

We thus sought to identify assemblies of neurons which had a statistical tendency to coactivate, both within and across brain structures. To this end, we used PCA followed by independent component analysis (PCA-ICA) on the binned spike trains of neurons from all recorded regions during a time window spanning the start of the offer cue to the end of the action window (STAR methods^41,42^). For this analytical pipeline, the spiking of all neurons was binned at 25ms and z-scored. PCA was then applied, and significant principal components were identified based on the size of the associated eigenvalue (a read out of the variance explained by the principal component). ICA was then used to fine-tune the significant assembly patterns from PCA, allowing the analysis to reflect higher order correlations and removing the requirement for assembly patterns to be orthogonal from one and other^28,41,42^. This pipeline outputted a vector for each significant assembly pattern, which contained the weight of each neuron in the assembly. The higher a neuron’s weighting, the greater the contribution it makes to the assembly.

This approach yielded a total of 618 assemblies. Importantly, several significant assemblies could be detected in a single recording (median=10 assemblies per recording) (Fig S8), suggesting that we were not simply detecting some global tendency for neurons to coactivate. Most had ‘member neurons’ (i.e., neurons with a high weighting in the assembly pattern) restricted to a single brain region (~83% Fig. 4e, g). The remaining assemblies had member neurons that spanned multiple structures (Fig. 4f, g), including a small proportion (13/618) which were made up of member neurons spanning 3 or more brain regions (e.g., Fig. 4f). The proportion of assemblies that had member units from within a single structure was significantly greater than chance (Fig. 4g).

We then examined whether such assemblies provide a unified read-out of canonical decision variables. To this end, the mean expression of the coactivity pattern was modelled as a weighted linear sum of reward, effort and choice (STAR methods). Strikingly, nearly half (48%) of all identified assemblies had a significant degree of selectivity for the prospective reward, current effort cost and choice, either individually or conjointly (Fig. 4h). Several distinct assemblies identified in a single recording session could be found encoding the same variable (e.g., Fig. S8), suggesting that the coactivity that encodes canonical variables was not a global brain-wide phenomenon but instead resulted from the co-firing of distinct combinations of units. Moreover, assemblies devoted to encoding each of the canonical decision variables were found in each recorded animal (Fig. S11). Jittering the spike trains (Fig. S9) preserved the mean firing rates over trials and almost all the rate-coding within the window of analysis (Fig. S10), but substantially disrupted coding at the level of the assembly (Fig. 4i).

We next sought to determine if the coactivity and assembly encoding we observed here was a result of the locking of units to task events. In order to investigate this, for each neuron we shuffled spike trains across trials (for further details, see Fig. S9) of the same level of reward, effort or choice outcome. This exactly preserves the firing rate of neurons over the window investigated while also controlling for any coactivity that arises from the synchronised locking of units to task events. Trial-shuffling the spike trains (Fig. S9) disrupted effort and choice - but not reward - coding (fig. S12), owing to the highly synchronised activity around the onset of the reward cue. In these cases where many neurons lock their spiking to an external event, the formation of assemblies cannot be dissociated from event-related firing. However, for both choice and effort, it is clear that assembly coding cannot be accounted for by firing driven by the task structure. Overall, assemblies thus provided a substrate of encoding that coordinates activity across populations of neurons but is independent of the underlying correlated changes of firing rate at task-relevant timescales.

To understand how each region contributed to assemblies, we examined the likelihood (i.e., counts normalised by the total number of neurons from each structure recorded in a day) of neurons from any structure forming assemblies regardless of their underlying tuning properties. We found that DMS neurons were slightly more likely to be members of co-active assemblies (Fig. 4j) than would be expected due to chance. We then examined whether neurons from each area were more likely to be members of assemblies coding for one of the canonical decision variables (blue/purple bars, Fig. 4k) than assemblies that did not code for any of the decision variables (gray bars, Fig. 4k). Though the membership of task-tuned assemblies were relatively even across regions, STA neurons were more than twice as likely to be members of reward-coding assemblies than those that did not encode any decision variable (Fig. 4k). In contrast, ACC neurons were *less* than half as likely to be members of assemblies encoding reward than assemblies that did not encode any variable (Fig. 4k). Cross-structural assemblies were not significantly more likely to encode any of the decision variables (Fig. 4l). Together, this demonstrates that assemblies were formed by neurons in each structure across the entire network, with STA neurons in particular having an important role in the expression of reward-related co-activity.

### Assemblies that encode decision variables are comprised of individual neurons with diverse tuning properties

The above analyses demonstrate that both individual neurons *and* assemblies encode and integrate the canonical decision variables. One possibility therefore is that the assembly code is closely correlated to each individual neuron’s tuning selectivity. However, it is also technically feasible for the temporal code within an assembly to be distinct from the rate code exhibited by the members of that assembly.

**Figure 5:**
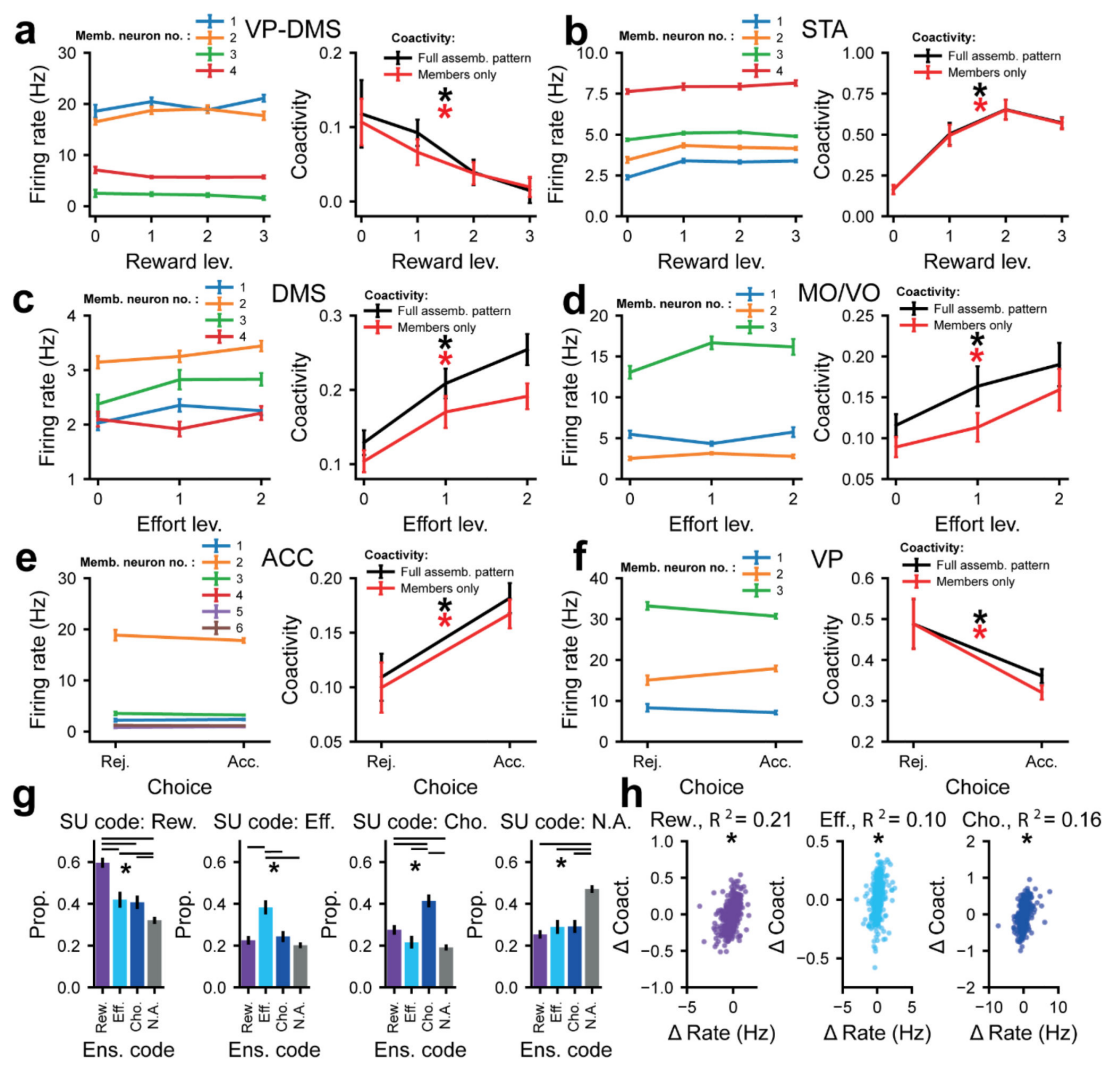
Decision-related encoding by co-firing assemblies can be orthogonal to the rate coding of the individual neurons that comprise them. **a-f**, example assemblies that significantly code one of the canonical variables (right). All of the member neurons that make up these assemblies do not significantly rate code for the same canonical variable as the assembly as a whole (left, the firing rate of all of the individual member neurons that make up an assembly dividing by levels of the canonical variable coded by the assembly as a whole). Firing rate and assembly pattern coactivity were calculated over the same window of time (the decision window, see STAR methods). The single units that made up assemblies (left) were coloured arbitrarily for display purposes. The anatomical structure an assembly was from was marked above them with text. **a, b**, the firing rate of all the individual member neurons that make up example reward coding assemblies (left). They show no significant change across reward levels. Assembly coactivity (right) computed using the full assembly pattern weights (black) or restricting weights to only member neurons (red). Both the coactivity for the full assembly pattern and the coactivity patterns restricted to only member neurons are significantly correlated with the offered reward magnitude. **c, d**, the firing rate of all the individual member neurons that make up example effort coding assemblies (left). They show no significant change in firing rate across effort levels. Assembly coactivity (right) computed using the full assembly pattern weights (black) or restricting weights to only member neurons (red). Both the coactivity for the full assembly pattern and the coactivity patterns restricted to only member neurons are significantly correlated with the level of effort. **e** and **f**, the firing rate of all the individual member neurons that make up choice coding assemblies (left). They show no significant change in firing rate with choice outcome. Assembly coactivity (right) computed using the full assembly pattern weights (black) or restricting weights to only member neurons (red). The choice outcome has a significant effect on the coactivity pattern of both the full and members-only assembly pattern. For a-f, * p<.050, significance was determined using linear regressions with firing rate or coactivity as the dependent variable and reward, effort and choice as the independent variables. The CPD of reward, effort and choice outcome was compared to the null distribution from trial shuffled data. Significance of the coding for both single units and assemblies was defined in the decision window. **g**, the proportion of member neurons of reward, effort, choice and non-encoding assemblies which rate code reward (left), effort (middle-left), choice outcome (middle-right) or do not rate code for the canonical variables (right). All assemblies from across all animals and brain structures were pooled in this analysis. Significance of the coding for both single units and assemblies was defined in the ‘decision window’ (see STAR methods). *p <.050, z-test, Benjamini-Hochberg procedure used to control for multiple comparisons. **h**, the change in the firing rate of member neurons across the levels of reward (left), effort (middle) and choice (right) outcome only explains a small proportion (<22%) of the change in assembly expression strength (see STAR methods). All of the assemblies from across all animals and brain structures were pooled in this analysis. The change in firing rate of single units and assembly coactivity was calculated over the ‘decision window’ (see STAR methods). * p < .050. Rew. = reward, Eff. = effort, Cho.= choice. Rej.=rejected trials, Acc.=accepted trials. Reward levels 0, 1, 2, 3 represent nil, low, medium and high reward respectively. Effort levels 0, 1, 2 represent the number of barriers in the linear track.

To address this, we asked whether ensembles that encoded a given decision variable were comprised of neurons that *rate-coded* the same variable. Strikingly, we found examples of assemblies where the rate-coding of individual neurons clearly diverged completely from that of the overall ensemble (Fig. 5a-f). To quantify the intersection of the encoding properties of single neurons (Fig. 2f) and assemblies (Fig. 4h), we defined the proportion of each type of rate-coding neuron that contributed to each type of encoding assembly (Fig. 5g). Although single units that encoded a given variable were more likely to contribute to assemblies that encoded the same variable than any other assembly type (Fig. 5g), between approximately 40-60% of the neurons that contributed to assemblies did not code for the same variable as the assembly as a whole (Fig. 5g). Only a small proportion of the variance in the assembly pattern expression strength dynamics (<22%, for reward, effort and choice) across levels of decision variables could be explained by changes in the firing rate of member neurons (Fig. 5h), demonstrating that this bias was not simply a consequence of correlated, task-related firing rate across assembly members.

Taken together, these findings suggest that though a member neuron’s individual tuning often aligns with that of their assembly, assembly pattern expression strength changes were poorly explained by firing rate dynamics. Furthermore, neurons often contributed to assemblies encoding a different parameter to their own. Co-activity is thus separable from the rate code and can integrate units with diverse tuning properties.

### Encoding of reward, effort and choice by cortico-basal ganglia assemblies aligns with relevant task events

Our analyses of assemblies so far defined encoding using expression strength across the entire choice window. If assemblies provide population-level encoding that has ongoing influence on cost-benefit decisions akin to that proposed for single neurons, their temporal dynamics should align with key task epochs.

**Figure 6:**
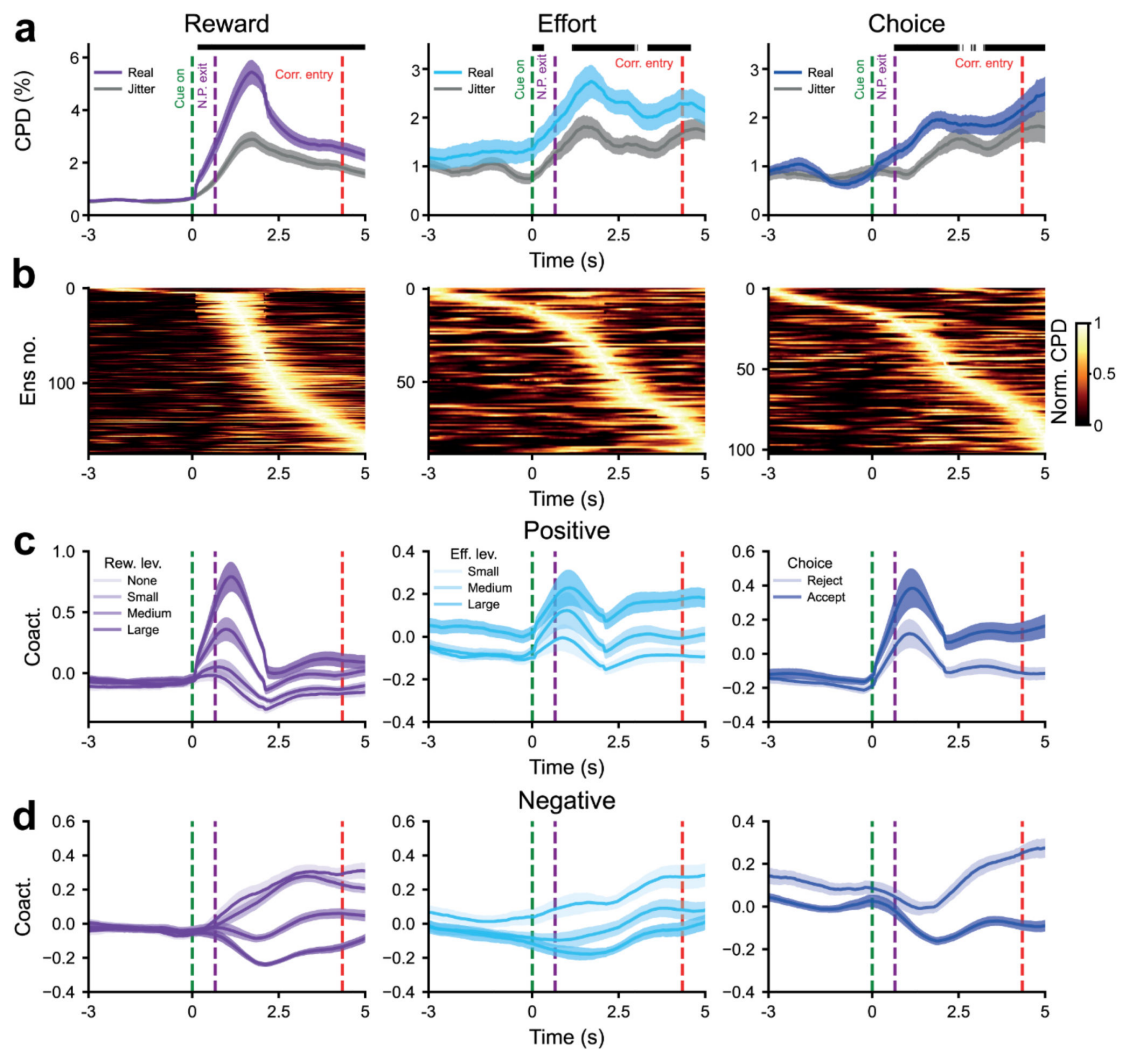
Co-firing assemblies can dynamically encode decision variables on task-relevant timescales. This figure shows the coding of canonical variables across time pooling across assemblies from all brain regions. We specifically looked at all recorded assemblies which significantly coded reward (left), effort (middle) and choice (right) (as in Fig 4h). **a**, CPD for reward, effort and choice (left to right) regressed onto the time-series of the expression strength of cell assemblies either with (grey) or without (blue/purple) the random jittering of the spike train in the range of -250ms to 250ms for all of the reward, effort and choice coding assemblies (left to right) (pooling assemblies across all brain structures and animals). Time of 0s is the onset of the reward cue and periods of time after cue onset where the CPD is significantly higher for the unjittered than the jittered assembly pattern expression strength were marked with a black bar (Wilcoxon signed-rank test, p < .050). The Benjamini-Hochberg procedure was used to control for multiple comparisons. There were no periods of significance prior to cue onset for reward, effort or choice coding assemblies (up to 3s before cue onset, Wilcoxon signed-rank test, all p>.050, Benjamini-Hochberg procedure). **b**, heat plot where rows represent the normalised coefficient of partial determination (CPD) for reward, effort and choice regressed onto the time-series of the expression strength of cell assemblies for each reward, effort and choice coding assemblies respectively (left to right) (pooling assemblies across all brain structures and animals). CPD was normalised by dividing by its peak value for each assembly and assemblies were sorted by the time of peak encoding. Time of 0s is the onset of the reward cue. **c-d**, the z-scored, smoothed (using a gaussian of $\text{std}=2/\sqrt{12}s$) coactivity across the different levels of decision variables for positively (**c**) and negatively (**d**) reward, effort and choice encoding assemblies (left to right) (pooling assemblies across all brain structures and animals). Increasing levels of reward and effort and accept trials are shown in darker colour. Time of 0s is the onset of the reward cue. Rew. = reward, Eff. = effort. Cue on = reward cue onset, N.P. exit = nose poke exit, Corr. entry = corridor entry.

To examine whether this was the case, we analysed the time course of encoding (as measured by CPD) for significant *assemblies* with respect to the mean onset of the reward offer, action initiation and nose-poke exit and subsequent accept/reject decision. Significance at a given timepoint was defined in relation to assembly coactivity constructed from jittered spike trains (Fig. S9; STAR methods). Reward coding assemblies encoded at above-chance levels during virtually all time-bins from the reward offer until the end of the decision point, whereas choice encoding assemblies were largely significant from shortly after the nose-poke exit to corridor entry (Fig. 6a, b). Effort encoding assemblies had patchier encoding that spanned from reward cue onset to corridor entry. As seen in the rate code of individual neurons, assemblies could encode positively (e.g., maximum expression strength for maximum reward) or negatively (e.g., maximum expression strength for minimum reward). Interestingly, the time course of assemblies that negatively encoded reward was more sustained/less phasic than for positively encoding assemblies (Fig. 6c, d).

Taken together, this shows that assembly co-activity - reflecting temporally co-ordinated activations within and across frontal and basal ganglia structures - dynamically encodes reward, effort and choice over timescales relevant to decision making.

## Discussion

Cortico-basal ganglia circuits play a key role in choosing actions based on information about prospective costs and benefits, yet the mechanisms behind this have remained elusive. To gain a comprehensive understanding of potential encoding schemes, we developed an “accept-reject” decision paradigm inspired by diet selection foraging^43^ and previous effort-based decision-making paradigms^44,45^. Single neurons, most prominently in DMS and STA, though also in all other recorded regions, signalled information about the reward offer following cue presentation, as well as the current effort block and then the accept/reject decision. Nonetheless, a substantial fraction of all recorded neurons displayed a wide range of temporal activity patterns across the task space. Crucially, these same neurons formed cell assemblies, dynamically encoding the cost-benefit variables and decisions to act through synchronisation of their spiking activity.

While there was an overrepresentation of reward coding neurons in the DMS and STA, for the most part, encoding was anatomically and temporally distributed across the cortex and basal ganglia. At least 20-30% of neurons in all five structures encoded *each* decision parameter, with neurons in each brain region having highly variable time courses for their coding. This temporally and spatially distributed code is consistent with that found in large-scale recordings with Neuropixel probes where there is widespread neural activity across brain regions coding for variables such as action and task-engagement^46^. Our findings are consistent with a shift from a modular understanding, where each brain region has distinct coding properties, to a more distributed model of brain function

Our behavioural results clearly demonstrate that both the current reward value and stored knowledge of the current effort requirement were used to determine the animal’s choice. Both neuroimaging^47^ and electrophysiological^36,48^ studies consistently implicate the MO, ACC and striatum in signalling the net effort-discounted value of reward. Here, we found that multiplexed combinations of reward and effort were more prevalent in basal ganglia than cortical structures, particularly in the DMS and STA. Striatal neurons have previously been shown to integrate reward with other decision variables^49,50^, but to the best of our knowledge this is the first report of this extending to conjoint cost-benefit coding.

Having demonstrated that rate-based encoding is generally distributed across the network, we hypothesised that synchronised firing could provide a means through which neurons with diverse tuning properties could be coordinated. We demonstrate that neurons across all the recorded cortico-basal ganglia regions can form cell assemblies, and that these assemblies can encode decision parameters. Cell assemblies have been mostly described in hippocampal neurons during spatial navigation^25,40^ and during the representation of contextual information^30^, but to our knowledge, have rarely been described in cortico-basal ganglia networks, and never for key cost-benefit decision variables^27,51^. Here we provide evidence that assemblies within and across the cortex, striatum and other basal ganglia structures (although particularly in the DMS and STA) can encode the levels of prospective reward and effort over timescales relevant to the utilisation of that information to regulate choice behaviour.

Assemblies promote firing within the membrane time constant (10-30ms) that is optimal to discharge downstream neurons / assemblies and for strengthening synaptic weights between those neurons^25^. Instead of acting as individual processing units, neurons in cell assemblies, both within and across structures, represent information cooperatively via their synchrony. This synchronous firing can produce a composite downstream affect that cannot be achieved by single neurons alone^25,26,40^. These spatiotemporal interactions in the frontal cortex and basal ganglia could promote integration of network-level connectivity akin to that in hippocampal networks, where the key property of the assembly is to simultaneously mobilize enough pyramidal neurons to discharge downstream neurons and promote synaptic plasticity.

Strikingly, assemblies that encoded a given decision variable were often comprised of single neurons coding for either a different parameter or none of the canonical variables at all (see schematic representation in Fig. S13). Equally we demonstrate via various analytical controls that assembly activity does not trivially result from single unit activity. This finding supports the idea that such assemblies are *emergent*: the activity of the population is more than a simple sum of the individual members, echoing previous findings in the hippocampus^30^. This has important implications for future studies of decision-making in these circuits, as it demonstrates that neurons that do not have clear, temporally consistent rate-coding responses (Fig. 2-3) can still play an important role at the population level (Fig. S13). This view is not mutually exclusive to neurons of a specific type having a specialized role within the network, as striatal neurons appear to do here. Rather, we suggest that higher-level emergent activity constitutes a parallel level of organisation that binds individual neurons with disparate rate coding properties.

Our findings suggest that while individual neurons across the brain can represent decision variables through a spatially and temporally distributed rate code, they can also do so via a population representation, formed of co-firing assembly members. These two coding schemas may increase the coding capacity of the brain by providing distinct channels for the representation of information and may support parallel computations within the same brain circuit.

### Limitations of the study

First, we endeavoured to restrict our analyses to a choice-invariant interval of the trial, we were not completely able to rule out the possibility that some unobserved behavioural difference (e.g. anticipatory licking, turning, nose-poking) may have contributed to the observed canonical decision variable coding. However, even when controlling for behavioural variables such as velocity and nose-poke exit latency post-hoc in our linear models, canonical decision variable coding tendencies in individual neuron firing rates were still identifiable.

Second, our trial-shuffling control showed that much of the coactivity that drives reward encoding cannot be distinguished from cue-locked neural activity. While this does not rule out a role for assemblies in the coding of reward, novel methods, including manipulations to prove causality, will be needed to prise this apart in future studies where widespread, short-latency task-related firing is present.

Third, despite attempts to target the projections from the MO/VO and ACC to the parts of the recorded basal ganglia^32,33^, we could not confirm that the neurons we recorded where monosynaptically connected across the 5 recorded brain regions. As such it is possible that a less distributed and more modular coding schema could be identified if we had greater anatomical precision. It is, however, reassuring that cross-structural assemblies could consistently be identified, suggesting that the brain regions we recorded were functionally connected.

Fourth, as we only used male rats, our findings our limited in generalizability across genders.

### Resource Availability

Lead contact: Andrew Sharott: andrew.sharott@bndu.ox.ac.uk

- Materials Availability: The study did not use any unique reagents or materials.
- Other items: Any additional information required to reanalyze the data reported in this paper is available from the lead contact upon request.

Further information and requests for resources and reagents should be directed to and will be fulfilled by the Lead Contact, Andrew Sharott (andrew.sharott@bndu.ox.ac.uk).

## STAR Methods

### Experimental Model and Study Participant Details

All procedures were carried out in accordance with the UK Animals (Scientific Procedures) Act (1986) and its associated guidelines. We used 12 group-housed Lister Hooded naive male rats (Charles River, U.K.) weighing 300-390g at the beginning of training. Rats were maintained on a twelve-hour light/dark cycle (lights on at 7 AM). During testing periods, rats were food restricted to a target weight range of 85-90% of their free-feeding weight. Water was available *ad libitum* in their home cages. Following implantation with multielectrode drives, animals were singly housed.

### Method Details

#### Behavioural apparatus

The operant box was designed in Fusion360 (Autodesk) and consisted of two 17.6cm diameter custom-designed 3D-printed plastic semi-circular arenas, connected by a 130 cm-long Perspex corridor with walls on either side. The corridor had 6 equidistantly placed infra-red (IR) light-gates to register the rats’ position and 3 equidistant slots for barriers to increase the travel cost between the ends of the corridor. Each arena housed a custom designed 3D-printed plastic nose-poke and reward port, positioned symmetrically on either side. Nose-poke and reward ports contained a infrared light-gate and a white LED house light. Reward ports were connected with 20-mg food pellet dispensers (Med-associates) fitted outside the walls of the arenas using medical-grade silicone tubing. Each reward port housed a moveable floor that was able to obstruct the port. The electronics were controlled by a Teensy 3.6 microcontroller that ran on custom-written firmware. The apparatus was lit using far-red LEDs to minimise visibility during training and testing.

#### Behavioural paradigm

Rats initiated trials by poking and holding in the nose-poke port of the starting arena (“pre-cue”). The starting arena was randomly allocated on the 1st trial. After a pre-cue period (0.3-0.4s), an auditory cue (“offer”) was again presented from the distal end of the apparatus to signal the available reward at end of the track from where they initiated. The available reward was pseudo-randomly varied trial-by-trial (4 sizes: nil, low, mid, high, corresponding to zero, one, three or six 20mg sucrose pellets), signalled by a distinct auditory cue. The cue-reward associations were counter-balanced across subjects. Rats were required to maintain nose-poking for another 0.6-0.8s until the onset of a visual Go cue, signalled by a white light LED. Premature exiting of the nose-poke either during the pre-cue or offer period would elicit a 5-s error time-out, indicated by a flashing house-light in the nose-poke, followed by a 5s ITI. If rats exited prematurely during the auditory cue, the same cue would be repeated on the next nose-poke attempt. A grace period of up to 100ms during nose-poking - where nose-poke exits did not count towards an error - allowed for minor adjustments in body position. To accept the offer, rats needed to enter the corridor (cross a light-gate 24cm away from the arena) within a time window; otherwise, that offer was rejected. The cost of pursuing reward was pseudo-randomly varied across blocks of 17-23 trials (3 levels: low, moderate, high), implemented using varying numbers of barriers (0-2 or 1-3 in implanted or non-implanted animals, respectively) in the corridor. The barriers were 0.5 cm thick and were 12 cm and 18 cm tall at their lowest and highest points respectively, with a 45-degree slant mid-way between to generate a more effortful zig-zag of running between the ends of the corridor.

#### Behavioural training

Rats were handled daily for a week prior to training. Training consisted of 5 steps in both versions of the task, summarized in table 3.

Step 1 (“magazine training”) consisted of rats being introduced into the apparatus with bedding from their home cages and receiving 20 rewards in each food dispenser, which they were free to consume in 20 minutes.

In Step 2 (“cue-reward association training”) - entry of the starting arena as detected by entry/presence in the arena of latest reward delivery would trigger one of 4 auditory cues, predicting the magnitude of the promised reward in the distal reward port. The offer cue was played until the animal made an entry into the distal reward port. Next, the reward port LED was activated for a 1s pre-reward delay, followed by delivery of the reward corresponding to offer cue. 2.5s after reward delivery, the moveable reward port floor was elevated in that same arena, resulting in the blocking of that port to indicate that reward would not be available there on the next trial. After the rats had reached a criterion of initiating 120 trials in £ 60 minutes, rats were taken to step 3.

In step 3 (“nose-poke training”), in v1, rats had to learn to sustain a nose-poke with a jittered 1-1.2s length in order to elicit the offer cue. If rats sustained a 0.3-0.4s-long nose-poke (“pre-cue”), one of 4 auditory cues would be played. Importantly, rats were not free to leave until another 0.6-0.8s later (“offer”) once a visual Go cue, signalled by a white light LED in the nose-poke port, had turned on. This was implemented to ensure a movement-free period following offer presentation for electrophysiological analyses. The pre-cue nose-poke requirement was incremented from 0.1s to 0.4s in increments of 25 ms after every successful trial. Once animals had learned to sustain successful pre-cue nose-pokes, the cue nose-poke requirement was incremented from 0.0s to 0.8s in steps of 20 ms after every successful trial. Once animals had learned to complete 120 trials with the full 1.2s nose-poke requirement, jitters of 0.1s (“pre-cue”) and 0.2s (“offer”) were introduced. To allow for small changes in body posture that may have caused the nose-poke sensor to stop registering an entry, a 0.1s grace period was introduced which did not result in a nose-poke error. Importantly, if a nose-poke was interrupted for more than 0.1s (the grace period) during the “offer” window, the same offer cue would be repeated during the next trial. This was not an intentional feature, but rather the side-effect of using a pre-determined sequence of offers. Once rats had reached a criterion of initiating 120 trials within 60 minutes, they were taken on to step 4.

In step 4 (“barrier training”) rats needed to nose-poke to elicit offer cues as outlined previously, but now needed to additionally learn to scale barriers when traversing the corridor between the arenas. Barriers were added in one by one in blocks of 40 trials, starting from 1 barrier and going up to a maximum of 3 barriers. Once rats had successfully managed to complete 120 trials within 60 mins, they were taken on to step 5.

In step 5 (“final training for non-implanted animals”) *r*ats were required to either accept or reject a distal reward offer available at the other side of the corridor (fig. 2). After the onset of the Go cue, to accept the offer, rats needed to enter the corridor within a 3s time window as determined by a light-gate placed near the border of the arena; otherwise, that offer is rejected.

The effort cost which was fixed across blocks of ~20 trials (3 levels: low – 1 barrier; moderate – 2 barriers; high – 3 barriers; table 3). Upon corridor entry, they were allowed a further 5s to traverse the corridor and reach the distal reward port. To ensure that animals did not suffer an additional time cost by choosing to pursue an offer on accepted trials, we sought to match the duration of accepted and rejected trials. This was done by shortening the ITIs on ‘accept’ trials on each given recording day by the previous day’s cohort median reward magazine entry latency (the interval between Go cue onset and reward magazine entry on accepted trials). If animals chose to reject a reward-effort offer by not running across the linear track, they were able to initiate a new trial on the same side of the track.

In step 5 (“post-surgery re-training”), the corridor entry and traverse times were increased to 15s and 10s respectively post-surgery to account for the additional constraints on implanted rats with a recording tether. Additionally, to avoid collisions between the implant and the barriers, the number of barriers per each level of effort was reduced by 1. Rats were considered ready for the recording experiment once they managed to complete 120 trials within 60 minutes.

#### Outcome devaluation experiment

Following standard testing, outcome devaluation was performed on a subset of rats (n=6, first cohort) in a within-subjects regular Latin square design. In devaluation sessions, subjects were placed in a solitary “feeding” cage with *ad libitum* lab chow for 1h prior to the start of the testing session. Rats were placed in a solitary “control” cage for 1h on control sessions. The outcome devaluation lasted 3 days altogether, out of which days 1 and 3 were testing days and day 2 was a baseline training day.

#### Multielectrode drive

For single unit recordings we used a bespoke driveable multielectrode device weighing ~20g. The implant was comprised of a lid which housed a 128-pin connector designed for Intan 64-channel head-stages (Intan Technology), a plastic corpus that housed the electrode movement mechanisms, glass tubing and a base. All components were assembled in house. For assembly, the elements of the corpus were first 3D printed and post-processed with UV radiation and propanol. Second, holes were drilled in the corpus to allow threading of electrode wires and the driving mechanisms. Third, driving support mechanisms were created using metal rods, plastic shuttles and screws. Fourth, glass tubing was introduced into the driving mechanisms as electrode supports. Fifth, strands of 50-micron diameter tungsten wire (California Fine Wire) were twisted and heated to create 4- and 6-strand electrodes (tetrodes and hextrodes, respectively). Sixth, these were threaded through the glass tubing and the rest of the implant corpus. Seventh, ground wire stubs (connected to ground wires on the animal during surgery, see below) were introduced into the implant using a dedicated set of stationary holes. Finally, the implant was sealed and the electrodes cut to length by hand.

Three of the subjects (gh001, gh009, gh010) reported here received an implant that targeted all 5 regions, whereas one subject (oh011) received a modified drive without the VP electrodes (Electrode table). Electrodes in the MO/VO, ACC, and VP were arranged as a row along one sagittal plane. DMS electrodes were as rows along two sagittal planes and STA electrodes were arranged in triangular fashion around a centre point. The ML and AP positions of each electrode in the drive were planned around a centre-point co-ordinate obtained as an average between readings in two different versions of a commonly used rat brain atlas ^54,55^ that visually offered the most exposure in each structure, while also avoiding the ventricles (Fig. S2a). Relative electrode positions in the AP and ML dimensions were planned around centroid co-ordinates.

**Electrode table:** Overview of the number of electrodes and channels in each subject and targeted recording area.

	Number of electrodes (channels) in targeted area				
	MOFC	ACC	DMS	VP	STN
oh011	4 (24)	4 (16)	5 (30)	0 (0)	3 (18)
gh001	4 (24)	4 (16)	4 (24)	2 (12)	3 (18)
gh009	4 (24)	4 (16)	5 (30)	2 (12)	3 (18)
gh010	4 (24)	4 (16)	5 (30)	2 (12)	3 (18)

#### Implantation of multielectrode drives for recordings in behaving rats

Electrode implantations were performed in a subset of expert animals (n=4; electrode table). Surgeries were performed under isoflurane anaesthesia (5% for induction, 1.5-2.5% maintenance) and oxygen (2L/min). Subcutaneous local anaesthetic (Marcaine, 2mg/kg, 2.5 mg/mL) and a non-steroidal anti-inflammatory drug (Metacam, 1mg/kg, 5mg/mL) were administered at the beginning of each surgery. Post-operative opioid analgesia (Vetergersic, 0.3 mg/mL, 0.03 mg/kg) was administered subcutaneously for three consecutive post-operative days.

Craniotomies were created above each of the targeted areas and *dura mater* thoroughly removed from the visible brain surface. The drive was aligned on the animals’ heads by using the centre-point of the STA electrode bundle as a reference. Next, the drive was lowered to target depth on a stereotaxic frame (David Kopf Instruments) based on the DV reading of the STA electrode bundle from brain surface. The implant was then secured to skull-attached screws using adhesive dental cement and dental acrylic. Recordings were referenced to two supra-cerebellar screws which were connected to the implant during surgery. Neuronal recordings began at least 2 weeks after surgery. Electrode placement was confirmed using histology.

#### Electrophysiology data acquisition and processing

Wideband and accelerometer data were recorded, amplified and digitised on 2 tethered Intan 64-channel head-stages at 20,000 Hz using an Intan acquisition board (Intan Technologies). No hardware filtering was applied during recording. Files were saved in binary format. Each electrode was lowered by at least 125 μm up to 2h ahead of each recording session to ensure identification of unique units every day.

### Quantification and Statistical Analysis

#### Histological data analysis

To verify electrode placements, after the experiments were completed, animals were deeply anaesthetised with isoflurane (5%) and intraperitoneal pentobarbital (3 mL, Pentoject, 200 mg/mL and perfused trans-cardially with phosphate buffered saline (PBS) followed by fixative solution (paraformaldehyde dissolved in PBS, 4%, wt/vol). For post-mortem fixation, the sectioned heads were further submerged in fixative for another 24h, following which brains were extracted and washed 3 times in PBS and stored in a PBS solution containing 0.02% sodium azide until sectioning. Brains were sectioned at 0.1mm thickness using a vibratome (Leica), immunohistologically stained for FoxP2 and TH, and mounted in Vectashield. Unfiltered “bright-field” epifluorescence microscope images were acquired using 5x magnification and Zen Blue Pro (Zeiss). Histological results are reported in Fig. S2-3.

#### Electrode localisation and histological verification

To estimate all the possible recorded areas (fig. S2b) and exclude cells yields from neighbouring regions, we (1) estimated each identifiable electrode tract’s DV end-depth from our epifluorescence imaging data, (2) averaged this across all identifiable electrode tracts to get an average end-depth, (3) estimated each day’s targeted structure based on a log of putative lowering distances averaged across electrodes within a region. We did not restrict the cell yield for the STA based on histological data. For other structures we restricted our cell yields to only the MO or VO for MO/VO, Cg1 and Cg2 for the ACC, CPu for the DMS; and VP only for the VP. Estimation of the position of the electrode tip on a given recording day was based on the estimated extent of electrode advancement (i.e. the number of turns of the screw driving the electrode within the implant) and the tract location in the histological data. Wherever neurons were detected on days where the anatomical localisation of the electrode tip suggested we were either out of the brain near a target structure or in a nearby white matter tract, but that electrode trajectory was consistent with being in the target areas, we presumed that the error was in the estimate of the electrode tip and these cells were included in the analysed cell yield for that area. Neurons were not included if the entire electrode trajectory was not on target.

#### Spike sorting

Clustering and curation of units were carried out in KiloSort2^56^ and Phy (available at https://phy.readthedocs.io/en/latest/), respectively. Both are open-source libraries optimised for high-density multielectrode recordings. Individual units were isolated from the broadband signal based on waveforms using the KiloSort2 automatic clustering algorithm. A number of quality control steps were used in order to verify the single units identified by KiloSort2. Each neuron was manually inspected in Phy, and multiunits were excluded. Single units were first screened by the shape of the waveform. Only neurons with a stable waveform with spikes present across the entire recording block were included in further analysis. Next, we inspected the ISIs of each single unit to ensure that a clear refractory period was present. Cluster cutting was used to remove any contamination by electrical artefacts. Finally, the cross correlation between single units and their waveforms were used to merge those single units with a matching waveform and shared refractory period. Finally, single units with a firing rate less than <0.1Hz were excluded. No multi-units were used in the reported set of analyses. Numbers of isolated single units and their firing rate characteristics are reported in Fig. 2 and fig. S2, respectively.

#### Behavioural data analysis

All data were analysed in Python. Numbers of subjects and data depiction details are indicated in Results, Fig. 1 and Fig. S1. A binomial mixed effects model using the pymer4 library ^57^ was used to regress the following fixed effects onto trial-by-trial choice:

- *reward*, coded as 0, 1, 3 or 6
- *effort*, coded as 1, 2, or 3 (0, 1, or 2 in implanted animals)
- *reward x effort interaction*, coded as the multiplication of those vectors
- *reward count*, coded as the number of pellets earned since the start of the session.

To safeguard against type I errors, we aimed to fit a maximal random effects structure whenever possible by modelling each predictor as a random, in addition to a fixed, slope ^58^. To account for day-by-day and subject-by-subject covariation in the data, random slopes were paired with a random intercept for either the testing day or the subject ID. Whenever model fitting failed to converge, random effects that fit close to zero variance were removed to simplify the random effects’ structure. The resulting decision model for the non-implanted cohort had the following formula:

$$\begin{array}{c} \;\text{accept}\;=\;\text{reward}\;+\;\text{effort}\;+\;\text{reward}\;\text{effort}\;+\;\text{reward}\;\text{count}\;+(\;\text{reward}\;|\;\text{date}\;)+(\;\text{reward}\;|\\ \;\text{subject}\;), \end{array}$$

while the implanted rat decision model was formulated as follows:

$$\begin{array}{c} \;\text{accept}\;=\;\text{reward}\;+\;\text{effort}\;+\;\text{reward}\;\text{x}\;\text{effort}\;+\;\text{reward}\;\text{count}\;+(\;\text{reward}\;+\;\text{effort}\;+\;\text{reward}\;\\ \;\text{count}\;|\;\text{date}\;)+(\;\text{reward}\;+\;\text{effort}\;+\;\text{reward}\;\text{count}\;|\;\text{subject}\;) \end{array}$$

For the outcome devaluation experiment, we added pre-feeding and its interaction with reward and effort as an additional predictor, resulting in the following formula:

$$\begin{array}{c} \;\text{accept}\;=\;\text{reward}\;+\;\text{effort}\;+\;\text{pre}-\text{feeding}\;+\;\text{reward}\;\text{effort}\;+\;\text{reward}\;\times \;\text{pre}-\text{feeding}\;+\;\text{effort}\;\text{x}\\ \;\text{pre}-\text{feeding}\;+\;\text{reward}\;\text{x}\;\text{effort}\;\text{x}\;\text{pre}-\text{feeding}\;(1|\;\text{date}\;)+(1|\;\text{subject}\;) \end{array}$$

All predictors were standardised to facilitate side-by-side comparison. P-values were obtained using the likelihood ratio test.

#### Firing rate modulation during task execution

For visualising task event-locked firing rates for representative neurons (Fig. 2b**)**, spiking counts were quantified in 10ms *non-overlapping* bins sampled in 10 ms increments -400ms to +400ms around the initiation of the nose-poke (termed “Pre-Cue”), -400ms to +400ms around the onset of the offer cue (termed “Offer”), -500ms to +500ms around the onset of the Go cue (termed “Go Cue”) and -500ms pre- to +1000ms following nose-poke exit (termed “Action”). Data were subsequently averaged across trials of the same magnitude of offered reward, the required effort and prospective decision, each time averaging across the other two variables. Peri-stimulus time histogram (PSTH) data were smoothed with a gaussian filter of standard deviation 5.0 bins for display purposes.

To facilitate alignment of firing rate data on the task (Fig. 3c), time on trial was transformed into a uniform bin space by dividing the Pre-Cue epoch (-400 ms before Pre-Cue up to Offer) into 16 bins, the Offer epoch (from Offer up to Go Cue) into 16 bins, the Go Cue epoch (from Go Cue up to Action) into 13 bins and the Action epoch (from Action to 0.9s before observed corridor entry on accepted trials or 0.9s before the session median of corridor entry latencies on rejected trials) into 18 bins. For justification of the 0.9s cut-off, please see the section “Defining the decision window” below. Firing rates were quantified in 50 ms bins centered around each such bin and z-scored relative to a concatenated series of ITI data from the ongoing effort block.

#### Defining the decision window

To isolate a portion of the trial that was invariant to velocity changes between accepted and rejected trials, and thus representative of the decision process without movement confounds, we used DeepLabCut^59^ to extract rats’ moment-to-moment head positions throughout videos recorded simultaneously to the electrophysiological data (fig. S4). We first examined the accuracy of position estimation in this dataset compared to the ground truth (light-gate activation). To do this, we visualised the DLC estimate of the rat head position during activation of one of four light-gates (left/right nose-poke or left/right reward magazine; fig. S4). We then calculated the percentage of DLC-estimated head positions that were within the true light-gate perimeter (fig. S4c-f). Accuracy across all sessions in our electrophysiology dataset was 77-92% (fig. S4d, f), with few instances of mal-positioned head locations scattered throughout the rest of the apparatus fig. S4a-b). We then compared speed estimates obtained from our DeepLabCut data in 100ms bins ranging from -3 to +3s from corridor entry (as measured by triggered light gates) on accepted and rejected trials, respectively, and serially carried out one-way ANOVAs on each bin with choice specified as the independent variable. The earliest time bin of significance was identified by extracting the first Benjamini-Hochberg-corrected p < .05 in the obtained series of p-values. This revealed that the speed profiles meaningfully diverged 0.90s before corridor entry (main effect of choice, F_1,2_ = 8.52, p = .027), driven by higher average speeds on accepted trials (Fig. S4g). This led us to establish a cut-off of 0.9s before corridor entry (adjusted corridor entry) as being the end of the decision window.

#### DeepLabCut Video Analyses

We carried out the above analysis on the positional data extracted from DeepLabCut. We trained the resnet_50 model on labelled frames from 664 videos (3 frames labelled per video) which spanned all four recorded animals across all configurations of barriers. Specifically, we labelled the position of the back of the head of animals, between their ears. The model was trained over 900,000 iterations until the loss had plateaued. Model performance was then checked by visual inspection of labelled videos and using the alignment with light gate data (see Fig. S4).

#### Individual neuron regression analyses

General linear models (GLMs) were employed to quantify the influence of reward, effort and prospective decision on z-scored individual unit firing rates. Only single units whose firing rates in any given time bin of analysis was less than or equal to 0.1Hz were excluded. The loading of each variable on z-scored firing rates were obtained using the closed-form least-squares solution:

$$\text{b}={\left({\text{X}}^{\text{T}}\text{X}\right)}^{-1}{\text{X}}^{\text{T}}\text{Y}$$

where X is a design matrix of shape [trials x variables] and Y is a vector of z-scored firing rates of shape [trials x 1] from one recording day. The 4 regressors used in the design matrix X were constructed as follows:

- Intercept: a vector of ones
- Reward: a vector containing the values 1, 3 or 6 corresponding to small, medium or large reward trials.
- Effort: a vector containing the values 1, 2 or 3 corresponding to low, moderate or high effort trials.
- Decision: a vector comprised of the values -0.5 and +0.5 corresponding to rejected or accepted trials.

All regressors except the intercept were z-scored to themselves before fitting the models and obtaining coefficient loadings. From these GLMs, coefficients of partial determination (CPD), reflecting the unique variance explained by variable, were obtained as follows:

$${\text{CPD}}_{\text{i}}=100\times \left(\frac{{\text{SSE}}_{\sim \text{i}}-{\text{SSE}}_{\text{i}}}{{\text{SSE}}_{\sim \text{i}}}\right)$$

where SSE_~i_ and SSE_i_ represent the sum of squared errors of models that, respectively, do not or do include the regressor of interest. Using CPD ensured that the encoding strength for a given variable was not secondary to another (e.g. higher firing rate/encoding on accept trials due to more of these trials having higher prospective reward). To identify significant coding, permutation testing was used. For each neuron and decision variable, 1,000 shuffled datasets were generated by randomly shuffling the trial indices within each recording day. Such shuffling preserves potential autocorrelations between task variables and firing rates and is thus an appropriate estimate of the chance-level outcome. Individual neuron regression coefficient p-values were calculated as the fraction of (1,000) permuted CPD values which exceeded a neuron’s non-permuted CPD.

Significant neurons (Fig. 2f; Fig 3b-c: Fig. S5-7) were identified on the basis of CPD p-values less than 0.05 in *one single time bin* abutting the onset of the offer cue to 0.9s before observed corridor entry on accepted trials or the session median thereof on rejected trials. Individual neuron examples in Fig. 2b were selected if they met this criterion and displayed some consistency in the discrimination response over time based on visual assessment.

To elucidate fine temporal dynamics contributing to encoding reported Fig. 2f, regression results (Fig. 3a-b) and firing rates (Fig. 3c; Fig S5-7) were displayed as a time series of overlapping 50 ms time bins. These centre-points of those bins were obtained by dividing the Pre-Cue epoch (-400 ms before Pre-Cue up to Offer) into 16 bins, the Offer epoch (from Offer up to Go Cue) into 16 bins, the Go Cue epoch (from Go Cue up to Action) into 13 bins and the Action epoch (from Action to 0.9s before observed corridor entry on accepted trials or the session median thereof on rejected trials) into 18 bins. For justification of the 0.9s cut-off, please see the section “*Defining the decision window” above*. Regression models (fig. 3a-b; Fig S5-7) were fitted on each resultant bin. The firing rate time series in Fig. 3c were z-scored relative to a concatenated series of ITI data from the ongoing effort block.

Instantaneous population CPD p-values (Fig. 3a) were calculated as the fraction of permutations for which the (1,000) shuffled population average CPDs were larger than the non-permuted population mean CPD. The Benjamini-Hochberg (BH) procedure was applied to correct for false discovery rate across tested time points. The proportions of significantly coding neurons between regions were compared pair-wise between areas using the z-test of proportions with BH correction for the number of unique comparisons.

#### Quantification of CPD density (fig. 3a insets)

To characterise the degree to which individual unit CPDs magnitudes were temporally localised vs distributed across the trial timeline, we calculated *density*. This is related to *sparsity* as defined by previous studies ^28,60^ and indexed the extent to which the CPD time series for a population of neurons was dominated by a small number of time points. For each neuron in each area, it was defined as follows:

$$\frac{\sqrt{n}-\sum \;\left|v_{i}\right|}{\sqrt{n}-1}$$

where n is the length of the CPD vector (i.e., the number of time points) and v_i_ is the CPD vector’s i^th^ element. For this analysis, the input CPD time series was a unit vector where the sum of the squares of all elements added up to one. Density distributions were compared between brain areas using the two-sided Mann-Whitney U test with BH correction for the number of pairwise comparisons. All Mann-Whitney U test results reported in this work as expressed as a Z-score obtained as:

$$z=\frac{U-\frac{n_{A}n_{B}}{2}}{\sqrt{\frac{n_{A}n_{B}\left(n_{A}+n_{B}+1\right)}{12}}}$$

where U is the Mann-Whitney U test statistic and n_A_ and n_B_ represent the sample sizes in the two comparison groups.

#### Quantification of latency to peak coding response and phasicness (fig. 3a, lower panel)

To quantify the latency to peak coding response, we calculated the time bin of maximal CPD magnitude for each neuron and plotted the distribution across all neurons as violin plots using the python Seaborn library. To evaluate whether these peak CPD latencies tended to co-occur close-by in time (phasic responding) more than expected by chance, we compared these values with a null-distribution formed by bootstrapped sampling of all observed unique latency values with replacement to match the original number of sampled neurons. The null and real distributions were compared using the two-sided two-sample Kolmogorov-Smirnov test and the false-discovery rate corrected for by using the BH correction. The z-scores of the obtained test statistics were calculated as follows:

$$z=\sqrt{\frac{n^{2}}{2}\cdot D}$$

where n represents the size of both the original and bootstrapped distribution and D represents the two-sample Kolmogorov-Smirnov test statistic.

#### Quantification of single unit firing rate encoding of effort change (fig. S5f)

To investigate whether there were such transitions in the neural activity, we visualised the z-scored firing rates on the first five trials of any given block and organised them by the magnitude of the effort change (fig. S5e). We then calculated the Pearson’s correlations between the firing rates and effort delta scores on a unit-by-unit basis and identified significantly coding as those whose correlations had a p-value < .05.

#### Co-activity analysis

Assembly patterns were determined using the combination of principal component analysis and independent component analysis (PCA-ICA) as detailed previously ^41,42^. This analysis was conducted using neurons from all brain structures simultaneously over 68 recordings across the 4 implanted rats. This allowed for the unsupervised extraction of assembly patterns which could be contained entirely within an anatomical structure or could span multiple brain regions. Spike trains were taken from full trials (on a trial-by-trial basis) on the linear track after cue onset. That is, for accepted trials spike trains were taken from cue onset to 2s after reward delivery. On rejected trials, where the animal did not enter the corridor, spike trains were taken from cue onset to cue offset when the window in which rats could enter the corridor and receive a reward had elapsed. The spike trains for all units during time periods specified above were binned at 25ms, z-scored and concatenated into a *n* x *B* matrix, where *n* was the number of neurons and *B* was the number of 25ms bins. The element of this matrix at position (i, b) was the z-scored spike count of neuron i in bin b.

PCA was then applied to the z-scored spike count matrix. The number of significant patterns embedded in the z-scored spike counts was determined using the Marcenko-Pastur law. This is an analytical result from random matrix theory, which bounds the magnitude of the eigenvalues from PCA that can occur due to chance. Principal components with eigenvalues above the upper limit of the bound set by the Marcenko-Pastur law were classified as significant. Principal components with an eigenvalue greater than this threshold captured more of the correlation which existed between the binned firing of neurons than should occur if all neurons spiked independently of one and other ^28^.

The z-scored spike count matrix was then projected onto the significant principal components and ICA was applied to the resulting matrix. The output of ICA was an unmixing matrix. This unmixing matrix was then projected back onto the original basis, where each dimension represents an individual neuron. The columns of the resulting matrix represent assembly patterns. These assembly patterns contain a weight for each neuron. The greater this weight is, the greater the contribution the neuron makes to the identified coactivity pattern ^28^.

There are several advantages to using the combination of PCA and ICA as opposed to using either technique alone ^28,41,42^. For instance, PCA has been found to group several assembly patterns into the first principal component. PCA also artificially constrains identified assembly patterns to be orthogonal to each other, meaning that a high weighting of a neuron in one assembly pattern limits the weighting of that neuron is subsequently identified patterns. Finally, PCA is only based on pairwise correlations and cannot identify higher order correlations. However, if ICA was applied directly to the z-scored binned spike count matrix, one could identify the same number of assembly patterns as there are neurons. This could result in the extraction of spurious patterns. To address these caveats, ICA is applied to the z-scored binned spike count matrix following dimensionality reduction using PCA. For an overview of the total number of assemblies, including their partition by canonical decision variable coding, please refer to Fig. 4.

In order to determine the expression of assembly patterns across time, the outer product of each assembly pattern was determined^28^.The expression strength (R) of assembly pattern k can be determined as follows:

$$R_{k}(t)=z{\left(t\right)}^{T}P_{k}Z(t)$$

where P_k_ is the outer product of assembly pattern k with elements in the main diagonal set to 0, and z(t) is a column vector containing the smoothed z-scored firing rate of neurons at time t. The main diagonal of *P*_*k*_ must be set to zero to ensure that assembly pattern expression strength cannot be driven solely by isolated increases in the firing rate of individual units. The instantaneous firing rate of neurons in *z*(*t*) were computed by convolving the spike train of each neuron with a gaussian kernel and then z-scoring the resulting timeseries for each neuron. The standard deviation of the gaussian kernel was set to $25/\sqrt{12}$ ms to match the 25ms bin used for PCA-ICA ^61^. Convolution with a gaussian kernel allows for greater temporal resolution when compared to the 25ms bins which were used to define the assembly patterns via PCA-ICA.

Linear and logistic regression was conducted using either the python module statsmodel or in custom written pytorch functions. The general strategy utilized in the linear regressions was to take an epoch in each trial, determine the value of variables such as the spike rate or the mean coactivity in this epoch and use this to predict reward, effort or choice outcome across trials, or vice versa.

For identifying coding ensembles (as in single units), the mean expression strength of an ensemble was calculated from an interval starting with offer cue onset and ending either 0.9s before corridor entry (adjusted corridor entry) or, in instances where the animal did not enter the corridor, the median duration taken for corridor entry on that recording session less 0.9s (see above, in Defining the decision window). This wide bin was necessary because co-incident spiking in shorter time windows was a relatively rare phenomenon and would have precluded meaningful identification of co-active ensembles. The mean expression strength was then fitted as the dependent variable in a linear regression with reward, effort and choice outcome as independent regressors. Significance was determined by comparing the CPD of reward, effort and choice to that of the 95*th* percentile of the null distribution where reward and effort levels or choice outcomes were shuffled across trials. After identifying ensembles with significant modulation by the level of reward, effort or choice, we probed the time course of this modulation. This was achieved by fitting regression models with reward, effort and choice outcome as independent variables to the smoothed assembly pattern expression strength at different time points. Smoothing was achieved by convolution with a Gaussian with a standard deviation of $2s/\sqrt{12}$ and a duration of 2s. The convolved signal was aligned in time with task events such that only coactivity preceding a given time point was available to models. The CPD for reward, effort and choice was compared to that of linear models fitted to the assembly pattern expression strength in jittered spike trains.

The expression strength of assembly patterns was calculated again as part of 2 analytical controls (see Fig. S9 for more details):

- where the spike trains were jittered randomly in the range of -250ms to 250ms.
- where the spike train of each neuron was randomly shuffled across trials with either the same level of reward, effort or choice outcome.

Simple linear regression or logistic regression with decision variables (i.e., reward and effort level and choice outcome) as the dependent variable and mean assembly coactivity over the decision window as the independent variable was used to evaluate the ability of assembly activity to predict decision variables via R^2^ (linear regression- for reward and effort level) or McFadden’s pseudo-R^2^ (logistic regression-for choice outcome).

We tested the relationship between the coactivity change across levels of canonical variables and related this to the change in firing rate of member neurons across the levels of the same variable. We wanted to exclude the possibility that increases in coactivity were trivially a result of increases in the firing rate of member neurons. An example of this would be an assembly that’s coactivity increases with increasing reward driven entirely by more spiking across member neurons as the reward increased. Using the same window described above, for each assembly we modelled coactivity using multiple linear regression with reward, effort and choice as independent variables. We then stored the beta values from multiple linear regressions, representing the change in coactivity that occurs after increasing each of the canonical variables by one level. In the instance of choice, the beta coefficient represents the change in coactivity from a reject to accepted trial. We carried out the same procedure for each firing rate of each of the member neurons of each assembly. That is, we modelled the firing rate of these neurons using multiple linear regression with reward, effort and choice as independent variables. Again, we stored the beta variables from the linear regressions, representing the change in firing rate that occurs with an increase in each of the canonical variables by one level. For choice, the beta variable represents the change in firing rate from a rejected to an accepted trial. We then asked how much variance in the change in coactivity across levels of a canonical variable (using the coactivity-variable beta for each assembly) could be explained by the mean change in firing rate across member neurons (the mean of the firing rate-variable beta values across all of the member units of the assembly).

### Additional Resources

Data was analysed using the following open-source python packages/functions listed below (Key Resources’ Table).

**Key resources table:** Overview of all key code libraries used in this project.

REAGENT or RESOURCE	SOURCE	IDENTIFIER
Deposited Data		
Spike timings	This paper	https://data.mrc.ox.ac.uk.
Behavioural timestamps	This paper	https://data.mrc.ox.ac.uk.
Software and algorithms		
signal from SciPy: convolve(), used to convolve spike trains with Gaussians	Virtanen et al., 2020^62^	https://docs.scipy.org/doc/scipy/reference/signal.html
stats from Scipy: wilcoxon() used for Wilcoxon signed-rank test and mannwhitneyu() used for the Mann-Whitney U test ks_2samp() used for the Kolmogorov-Smirnov test	Virtanen et al., 2020^62^	https://docs.scipy.org/doc/scipy/reference/stats.html
decomposition from scikit-learn: PCA() and FastICA(), for assembly analysis	Pedregosa et al., 2011^63^	https://scikit-learn.org/stable/api/sklearn.decomposition.html
statsmodels.api: OLS() for linear regression	Seabold et al., 2010^64^	https://www.statsmodels.org/v0.10.2/
statsmodels.discrete. discrete_model: Logit() for logistic regression	Seabold et al., 2010^64^	https://www.statsmodels.org/v0.10.2/
statsmodels.stats. weightstats: ztest() for z-testing	Seabold et al., 2010^64^	https://www.statsmodels.org/v0.10.2/
NumPy: Matrix/array operations, helper functions, histogram() used to bin spike trains, corrcoef() used to compute correlation matrices	Harris et al., 2020^65^	https://numpy.org
random from NumPy: permutation() for data shuffling, rand() used for adding random jittering to spike timing	Harris et al., 2020^65^	https://numpy.org/doc/stable/reference/random/index.html
PyTorch: GPU parallelisation of matrix operations	Paszke et al., 2019^66^	https://pytorch.org
pandas: DataFrame(), used for the storage and manipulation of data	McKinney, 2010^67^	https://pandas.pydata.org
matplotlib.pyplot: ax.scatter(), ax.plot(), ax.bar(), ax.errorbar(), ax.hist(), ax.fill_between(), ax.imshow() used for data visualisation	J.D. Hunter, 2007^68^	https://matplotlib.org/3.5.3/api/_as_gen/matplotlib.pyplot.html
matplotlib_venn: venn3() for generating Venn diagrams	Konstantin Tretyakov (no accompanying paper)	https://pypi.org/project/matplotlib-venn/
Pymer4: Pymer4.Lmer used to fit linear mixed effects’ models in Python	Eshin Jolly 2018^69^	https://eshinjolly.com/pymer4/
Phy: Phy graphical user interface used for spike curation	Cyrille Rossant et al., 2016^70^	https://github.com/cortex-lab/phy
KiloSort2: KiloSort2 package used for spike sorting	Pachitariu et al., 2023^71^	https://github.com/jamesjun/Kilosort2
Custom code	This paper	https://data.mrc.ox.ac.uk.

## Supplementary Material

Supplementary Materials

## Data Availability

Data will be made available at https://data.mrc.ox.ac.uk.

## Code Availability

Core analysis code will be made available as of the date of publication at https://data.mrc.ox.ac.uk.
