# Supplementary material for: Multi-level encoding of reward, effort and choice across the frontal cortex and basal ganglia during cost-benefit decision making
Source: Cell Rep. Author manuscript; Available in PMC 2025 Feb 28. (PMC11860760; doi:10.1016/j.celrep.2024.115209)
Supplement: Supplementary Materials [file EMS203508-supplement-Supplementary_Materials.pdf]

Supplemental table titles and legends

Supplemental table 1. Direction encoding during the decision window. We regressed reward, effort and side of the available reward (left/right) on neuronal firing rates on accepted trials during the original decision window used in fig. 2f. Neurons encoding the side of the reward were ubiquitous across the targeted frontal-basal ganglia circuit (all proportions > 26%, all $p < .05$, one-sided binomial test against a chance rate of 5% with Benjamini-Hochberg adjustment for multiple tests). To clarify whether including the side of the reward led to a reduction in the proportion of reward/effort coding neurons in the DMS and STA, we compared the prevalence of reward and effort coding neurons between this model and the original firing rate model (fig. 2f) in all areas. We found that the original proportions were preserved (all $|z| < 1.17$, $p > .244$, two-sided z-test of proportions) except for reward-encoding neurons in the STA (36.25% in the original vs 29.44% in the current model: $z = -2.08$, $p = .038$, two-sided z-test of proportions).

Supplemental table 2, nose-poke exit latency encoding between Go cue onset up to nose-poke exit. To examine coding of latencies to respond, we focused on encoding of nose-poke exit latencies in the interval between Go cue onset up to nose-poke exit (see latency distributions depicted in Fig. S1f). A significant proportion of neurons in all structures coded for this variable (all $p < .05$, one-sided binomial test against a chance rate of 5% with Benjamini-Hochberg adjustment for multiple tests). Including this variable in the regression models revealed largely preserved multiplexing tendencies compared to the original model (fig. 2f) with minimal overlap of units encoding nose-poke exit latencies and other variables (all multiplexing units < 6%).

Supplemental table 3. Nose-poke error encoding during the Offer window. To examine premature nose-poke exit encoding (labelled cue nose-poke exit in this table), we focused exclusively on the Offer window (the interval between the onset of the offer cue and the Go cue) and asked whether the outcome of this interval– either a successful nose-poke or a premature nose-poke exit - was encoded in the firing rates of individual neurons, along with the other canonical decision variables. It is important to note that offer windows with successful nose-poke attempts were repeats of the same reward offer as encountered on any previous failed nose-poke attempt(s). Premature exits were coded by significant proportions of units in each area (all $p < .029$, one-sided binomial test against a chance rate of 5% with Benjamini-Hochberg adjustment for multiple tests). Compared to the original regression model (fig. 2f), this analysis demonstrated a decrease in the proportion of choice-coding units in all regions except the VP ($|z| = 0.90$, $p > .050$; all other $|z| > 2.69$, $p < .006$, z-test of proportions) - however it is important to note that there was relatively little choice coding during the offer window (fig. 3b). There was very little evidence of multiplexing of the two variables (all premature exit and choice multiplexing units < 6%), suggesting that these behaviours have different underlying encoding patterns.

Supplemental table 4. Run velocity encoding proportions. To examine how many neurons encoded running speed, we extracted the averaged head movement velocities from our DeepLabCut-processed video dataset during the interval between nose-poke exit and either

reward magazine entry on accepted trials or the end of the action period on rejected trials. As expected, the velocity distributions between the two trial types were meaningfully different (fig. S4e, $t_{8287.63}= -168.95$, $p < .001$, two-sample student t-test). Again, significant proportions of single units in all areas encoded both the canonical decision variables as well as velocity (all $p < .001$, one-sided binomial test against a chance rate of 5% with Benjamini-Hochberg adjustment for multiple tests). However, in this model reward encoding was less frequent than in the original model ($|z| > 2.49$, $p < .012$ in MO/VO, DMS and STA; $|z| < 1.36$, $p > .050$ in the ACC and VP), possibly owing increased temporal separation from the onset of the offer cue where coding was the strongest. By contrast, effort and choice coding was much more pronounced in this time interval ($|z| > 2.06$, $p < .039$ in all except the VP; VP: $z=1.66$, $p > .050$). Velocity was encoded by more than 33% of units in each area, with variable but mostly small degrees of multiplexing (up to 9%) with the canonical decision variables.

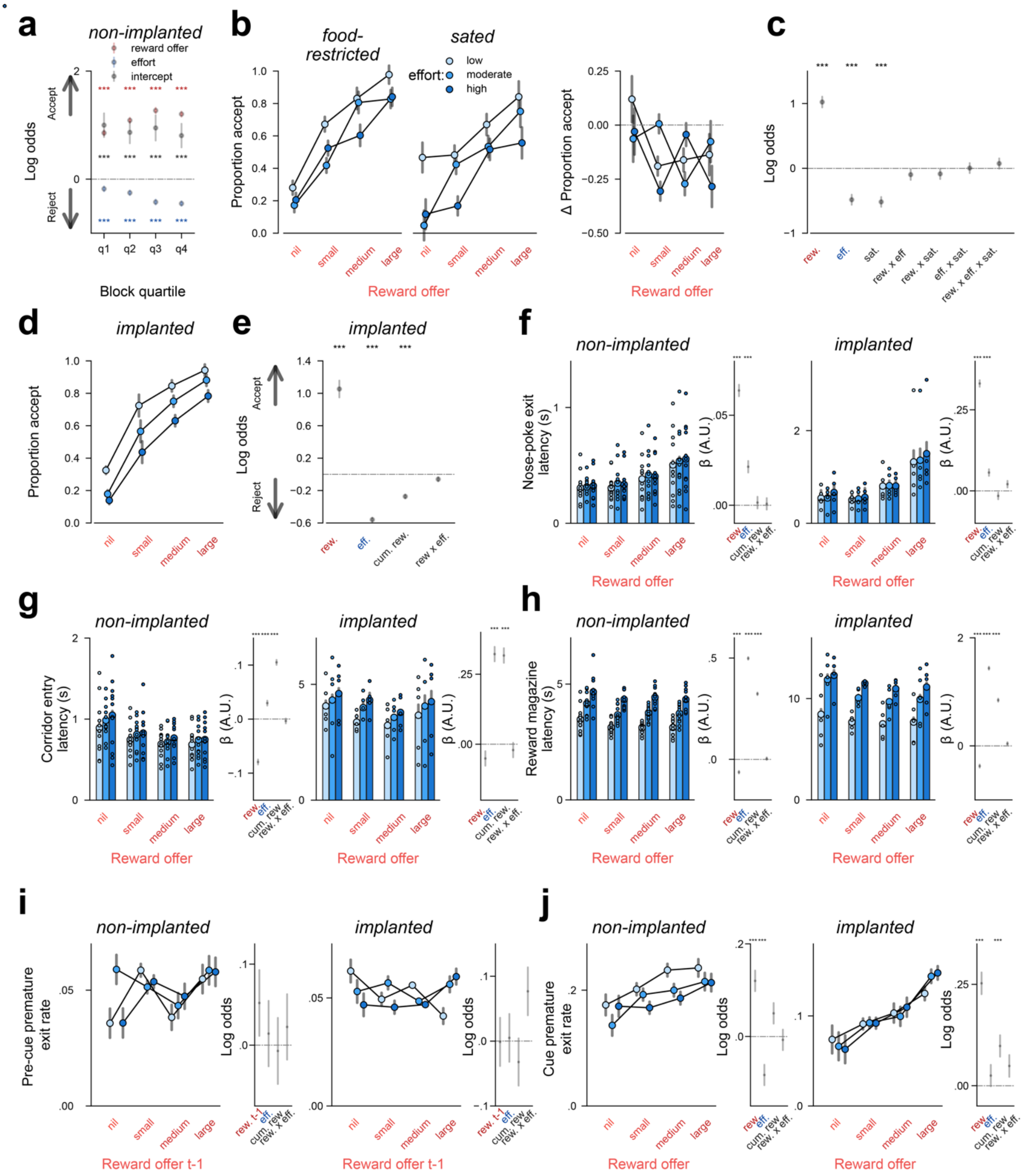

Supplemental Figure. 1 Choice on a rat accept/reject choice paradigm is shaped by the costs and benefits associated with the offer. a, weightings of reward and effort within quartiles of each effort block. Data are depicted as regression coefficients for reward (red) and effort (blue) ± SEM. ***, p < .01, corrected for family-wise error rate. **b,** Psychometric curves depicting rates of accepting offers as a function of reward (x-axis) and effort (black lines dotted with coloured scatter) from our within-subject outcome devaluation experiment (n=6). Data are depicted separately for rats' food-restricted and sated state on the left and right, respectively, with the difference (delta) scores between the sated and food-restricted proportions depicted on the right. **c,** weightings of experimental variables on behavioural choice from a binomial mixed effects' model fitted on data from the outcome devaluation experiment (n=6). **d,** Implanted rats' (n=4, sub-selected from the initial cohort of 12) rates of accepting offers as a function of reward and effort. **e,** weightings of task variables on implanted rats' choice from a binomial mixed effects' model. **f-h,** mean nose-poke exit (f), corridor entry (g), and reward magazine (h) latencies (left panels) and associated coefficient weightings from general linear mixed effects' models (right

panels, STAR methods). Nose-poke exit latencies were defined as the interval between cue onset and nose-poke exit, while corridor and reward magazine entry latencies were calculated as the time duration between nose-poke exit and closest corridor light-gate or reward magazine entry, respectively. Bars and larger scatters represent mean ± within-subject SEM, while small scatter dots represent per-subject median values. Colour-code is expressed as per panel **b**. **i-j,** mean pre-cue and cue premature nose-poke exit rate, respectively, and associated coefficient weightings from a binomial hierarchical model (STAR methods). Generalised linear mixed effects' models in i and j were fitted without nil reward trials. ***, $p < .01$, corrected for family-wise error rate using the Benjamini-Hochberg (BH) method.

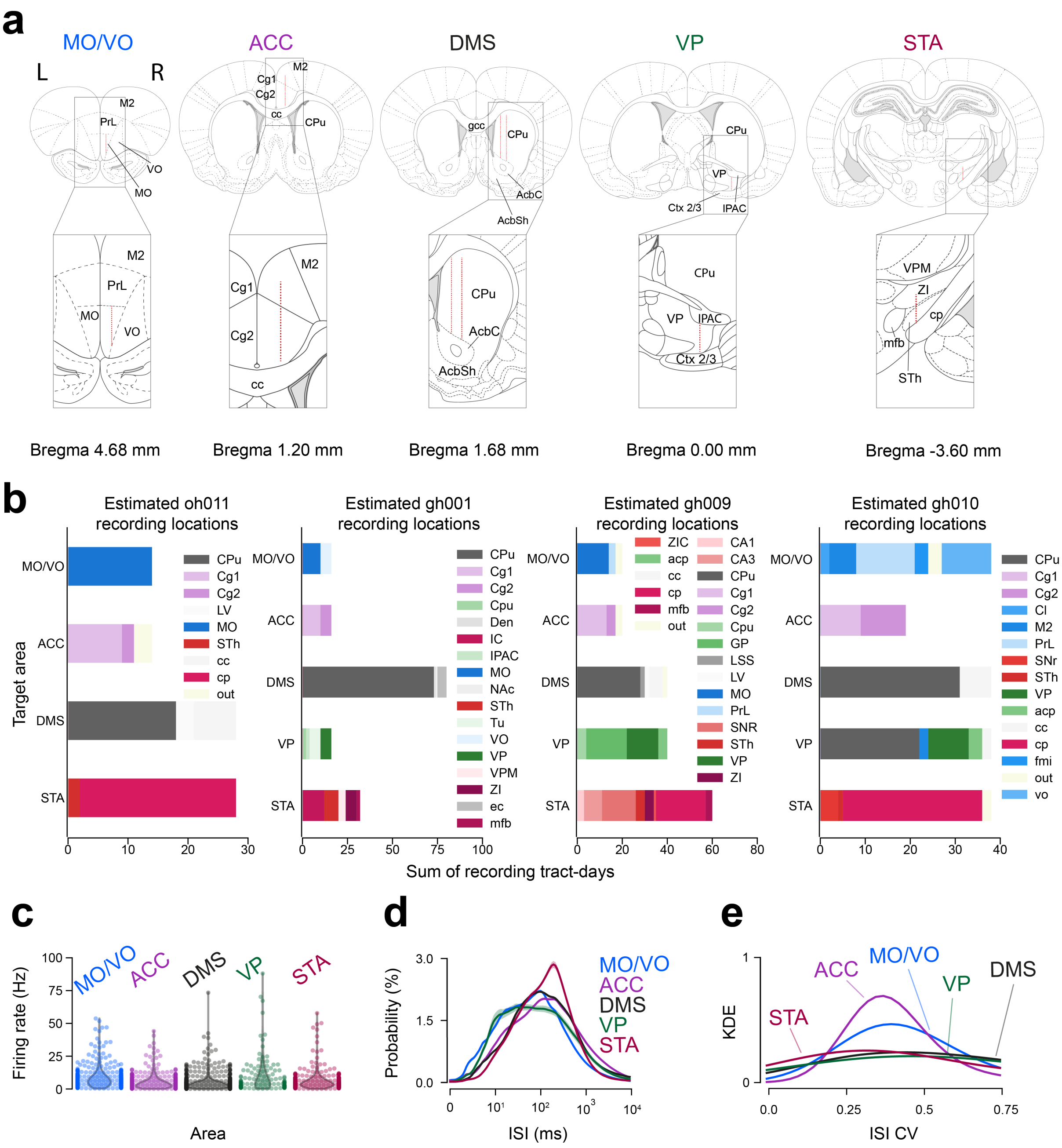

Supplemental Figure 2. Anatomic localisation and basic firing properties of single units recorded across five frontal-basal ganglia regions. a, planned electrode tracts. Electrode trajectories are depicted as dashed red lines superimposed on adapted atlas schematics on whole coronal sections (above) and zoomed insets (below; adapted from Paxinos and Watson 2006). **b,** breakdown of the number of recording days summed over histologically identified electrode tracts in each estimated anatomical location (coloured segments), grouped by the targeted structure (horizontal bars; blue - MO/VO; purple - ACC; grey - DMS; green - VP; red - STA) and animal (subplot). **c,** average firing rates of each identified neuron (dots) and kernel density estimates (KDEs) of the neuronal population (violin bodies) coloured by the targeted area. **d,** histogram of average inter-spike interval (ISI) values of neurons in each region. Values are depicted as the population mean probabilities (dark lines) ± SEM (shaded areas). Histograms values are smoothed with a gaussian kernel with a standard deviation of 1.0. **e,** KDEs of neuronal coefficients of ISI variation (lines, colour-coded by area). AcbC - nucleus accumbens core; AcbSh - nucleus accumbens shell; acp - anterior commissure, posterior part; cp - cerebral peduncle; CA1/3 - field CA1/3 of the hippocampus; Cg1/Cg2 - cingulate gyrus 1/2, cc - corpus callosum; cl - claustrum; CPu - caudate/putamen; cp - cerebral peduncle; ec - external capsule; Den - dorsal endopiriform nucleus; fmi - forceps minor of the corpus callosum; GP - globus pallidus; IC - inferior colliculus; IPAC - internal nucleus of the posterior limb of the anterior commissure; LV - lateral ventricle; LSS - lateral stripe of the striatum; M2 - secondary motor

cortex; mfb - median forebrain bundle; out - outside the brain; NAc - PrL - prelimbic cortex; SNr - substantia nigra reticulata; STh - subthalamic nucleus; Tu - olfactory tubercle; vo - ventral orbital cortex; VPM - ventral posteromedial thalamic nucleus; ZI - zona incerta.

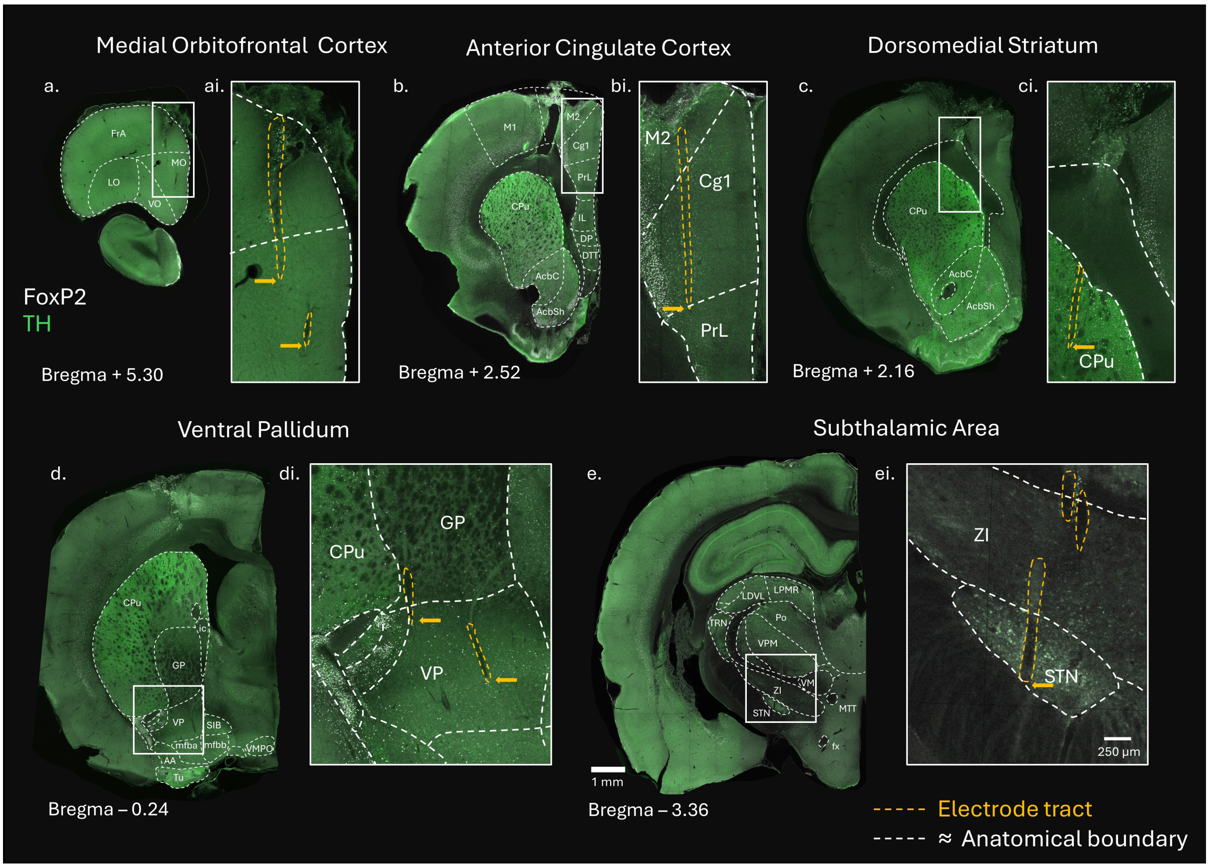

Supplemental Figure 3. Examples of histological targeting across five frontal-basal ganglia regions. a-e, histological images from coronal FoxP2 and tyrosine hydroxylase (TH) immuno-stained sections (to define striatum and STN, respectively) showing example electrode tracts in the five target regions. ai-ei, show enlarged inserts from each section highlighting the location of electrode tracts. Broken white lines indicate the approximate anatomical boundaries of both the target region and surrounding structures. Approximate anterior-posterior coordinates for each section from bregma (based on Paxinos and Watson, 2007) are shown on the bottom left (a-e). Broken orange lines indicate displaced or damaged tissue caused by the lowering of electrodes and solid orange arrows indicate the suspected location of the electrode tip. AA – anterior amygdaloid area; AcbC - nucleus accumbens core; AcbSh - nucleus accumbens shell; Cg1 - cingulate gyrus 1/2, CPu - caudate/putamen; DP – dorsal peduncular cortex; DTT - dorsal tenia tecta; FrA – frontal association cortex; fx – fornix; GP – globus pallidus; ic – internal capsule; IL – infralimbic cortex; IPAC - internal nucleus of the posterior limb of the anterior commissure; LDVL – laterodorsal nucleus of the thalamus ventrolateral part; LO – lateral orbital cortex; LPMR – lateral posterior thalamic nucleus mediorostral part; LSS - lateral stripe of the striatum; M1 – primary motor cortex; M2 - secondary motor cortex; mfba - median forebrain bundle ‘a’ component; mfbb - median forebrain bundle ‘b’ component; MTT – mammillothalamic tract; Po – posterior thalamic nuclear group, PrL - prelimbic cortex; VM – ventromedial thalamic nucleus; VMPO – ventromedial preoptic nucleus; VPM - ventral posteromedial thalamic nucleus; VO – ventral orbital cortex; ZI - zona incerta; SIB - substantia innominata basal part; STh - subthalamic nucleus; TRN – thalamic reticular nucleus; Tu - olfactory tubercle.

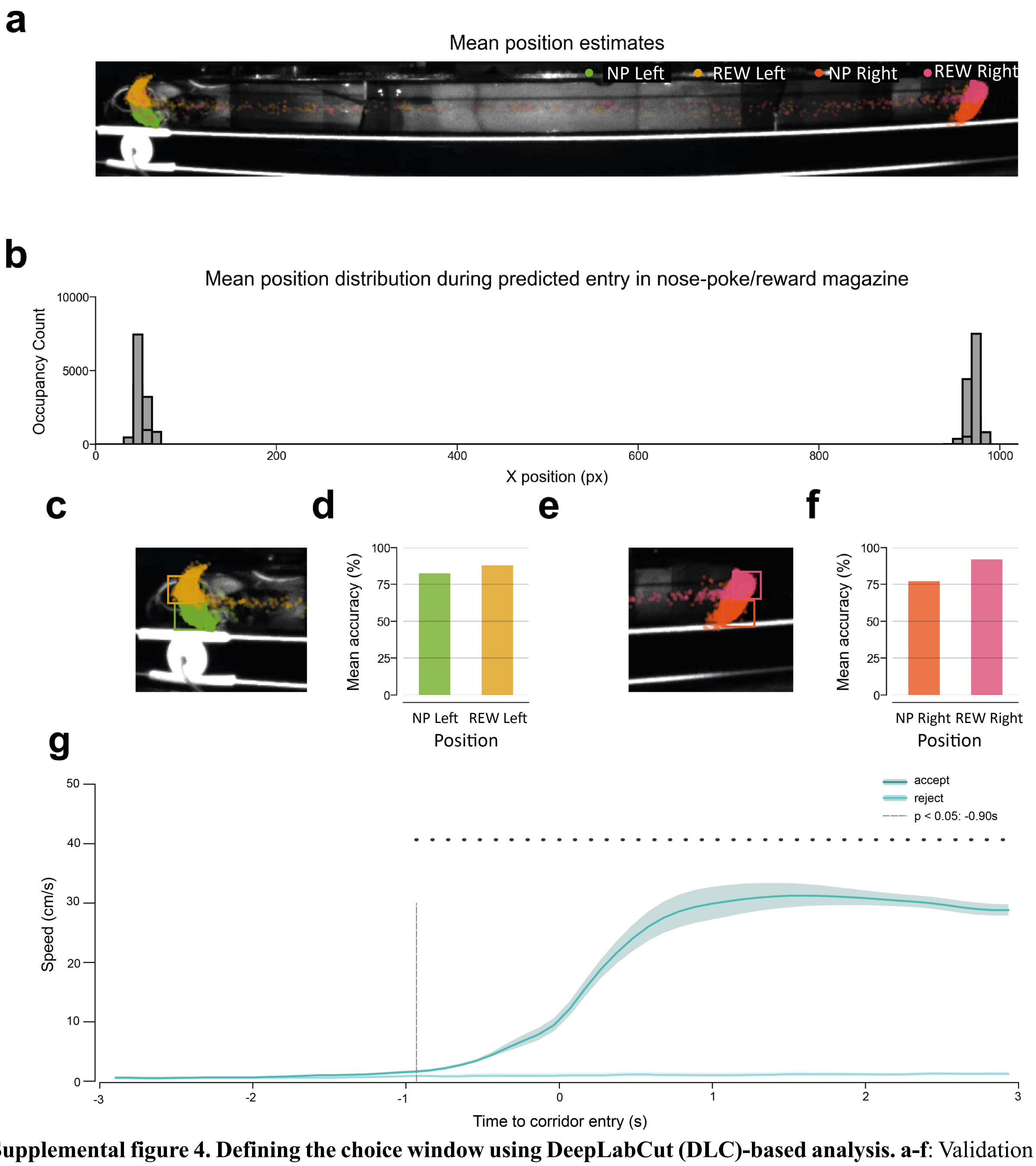

Supplemental figure 4. Defining the choice window using DeepLabCut (DLC)-based analysis. a-f: Validation of DLC position estimation accuracy based on light-gate data. **a**, Scatter of all instances in the entire recorded dataset where the rat should have been in either the left or the right nose-poke (NP Left and NP Right, respectively) during the reward cue or the left or right reward magazine (REW Left or REW Right, respectively) during the pre-reward delay. Each scatter point represents an average video position estimate within a trial at the point of a detected beam break in one of those locations. **b**, Distribution of the positions on the maze (x-axis) during these selected epochs. **c-f**, estimation of DLC position accuracy based on light-gate data. **c** and **e** illustrate the perimeters containing the reward magazine and nose-poke apertures. **d** and **f** depict the percentage of times where the predicted DLC position was within these areas. **g,** Velocity distributions on accepted vs rejected trials. Data is displayed as (trial-then-animal) average mean ± S.E.M on accepted (dark green) vs rejected (light green) trials -3 to +3s around entry of the light-gate closest to the corridor entrance. Dots above indicate any bin with a Benjamini-Hochberg corrected p-value < .05 obtained from one-way ANOVAs on each time instance (see above). The vertical line indicates the first significant time bin.

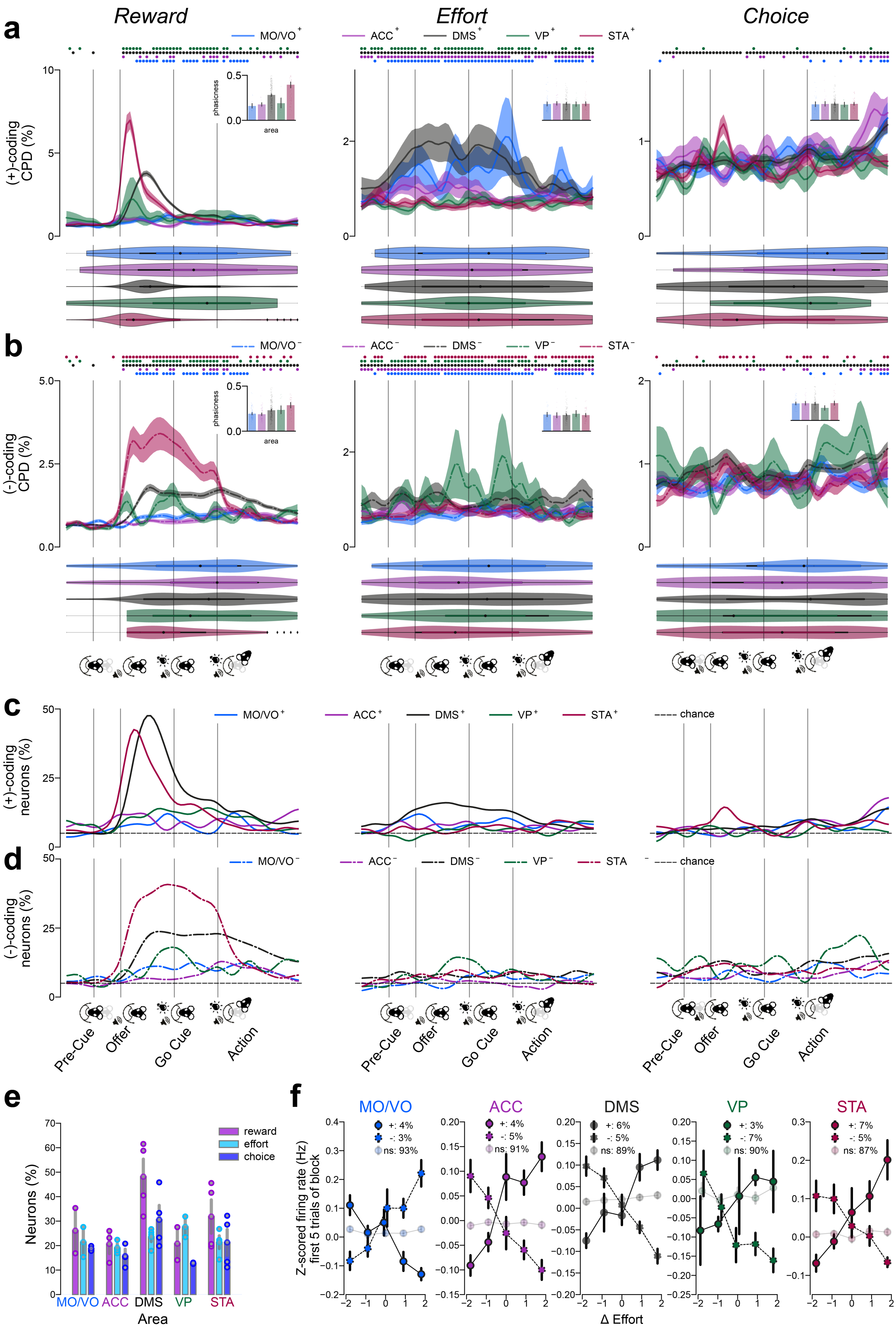

Supplemental Figure 5. Dynamic and variable single neuronal representations of reward, effort and decision across the frontal-basal ganglia network. a-b upper, CPD time series of neurons with positive (a) or

negative (b) tuning valence with regards to the canonical decision variables, depicted as means (lines) ± SEM (shaded areas) across all positively or negatively tuned units, for each decision variable (columns). Colour-coded dots above indicate time points where the population average CPD was significantly higher than expected by chance in each region using a significance threshold of $p < .05$ (permutation testing, fdr corrected across time points). Insets depict individual unit (coloured dots) and population average (bars) ± SEM phasicness scores for each area across the sets of positively (a) or negatively (b) coding units. Lower, latencies to peak CPD, depicted as kernel density estimates (violins) and superimposed boxplots. **c-d,** Instantaneous recruitment of positively (c) or negatively (d) coding neurons with a significance cut-off of $p < .05$. Time series in each line graph are smoothed with a Gaussian kernel of standard deviation 1.5 bins. **e,** single units coding for canonical variables were found in every animal. Data is represented as the per-cohort mean (bar), superimposed by between-animal error-bars representing SEM and scatter representing the proportions in each animal. Results are depicted only for those animals and areas where the total single unit count was above five. There were no differences between the percentages of units signalling any decision variable between the rats (all $\chi^2 < .16$, $p > .798$; Fig. 5). Further, within each animal, significant proportions of units in each area linearly signalled each canonical decision variable (all $p < .037$, one-sided binomial test against a chance rate of 5% with Benjamini-Hochberg adjustment for multiple tests). **f,** The relationship of firing rates on the first five trials of a novel effort block to the magnitude of effort change at block transitions. The data is depicted as degree of effort change (x-axis) at block transitions visualised against the mean (±SEM) firing rate of all neurons that had a positive, negative or non-significant relationship to the effort change (opaque, dashed, translucent, respectively; see figure legend) in the first five trials of an ongoing effort block.

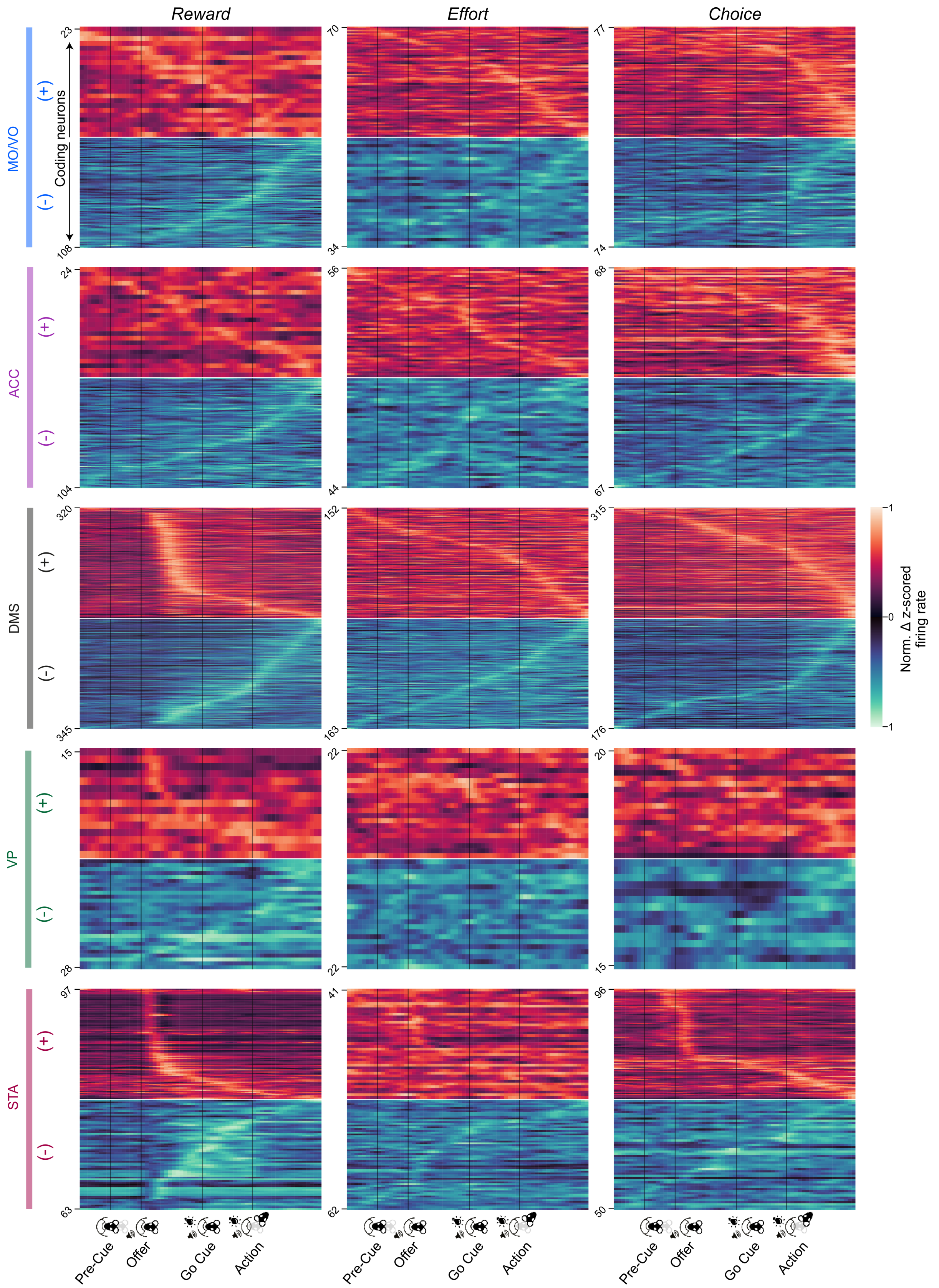

Supplemental Figure 6. Dynamic single neuronal representations of reward, effort and decision across the frontal-basal ganglia network. Data are depicted as normalized z-scored firing rate differences for each neuron (row) between the highest and lowest category of each decision variable and sorted by the time bin of peak difference. Heatmap and PSTH data are grouped by positive (above) or negative (below) tuning valence. Neurons

were selected on the basis of significant regression coefficients compared to trial-shuffled null distributions (STAR methods). Time series for each neuron are smoothed with a Gaussian kernel of standard deviation 1.5 bins.

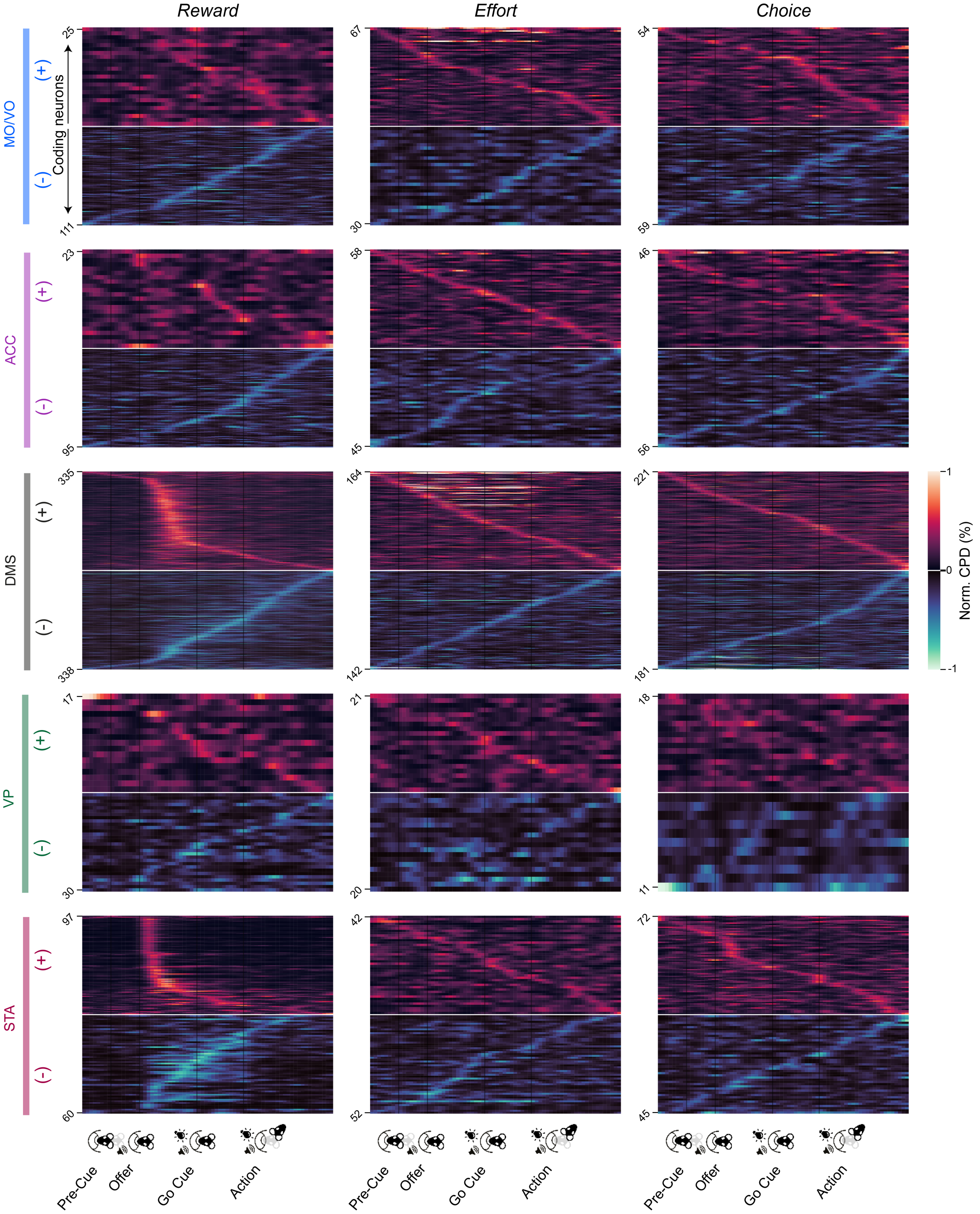

Supplemental Figure 7. Dynamic and variable single neuronal representations of reward, effort and decision across the frontal-basal ganglia network. Heatmap data are depicted as CPD values for each significantly coding neuron (row) and sorted by the time bin of peak CPD, while PSTH data represent mean ± SEM across all significantly coding neurons. Heatmap and PSTH data are grouped by positive (above) or negative (below) tuning valence. Neurons were selected on the basis of significant regression coefficients compared to trial-shuffled null distributions (STAR methods). Time series for each neuron on the heatmaps and for population-average traces are smoothed with a Gaussian kernel of standard deviation 1.5 bins.

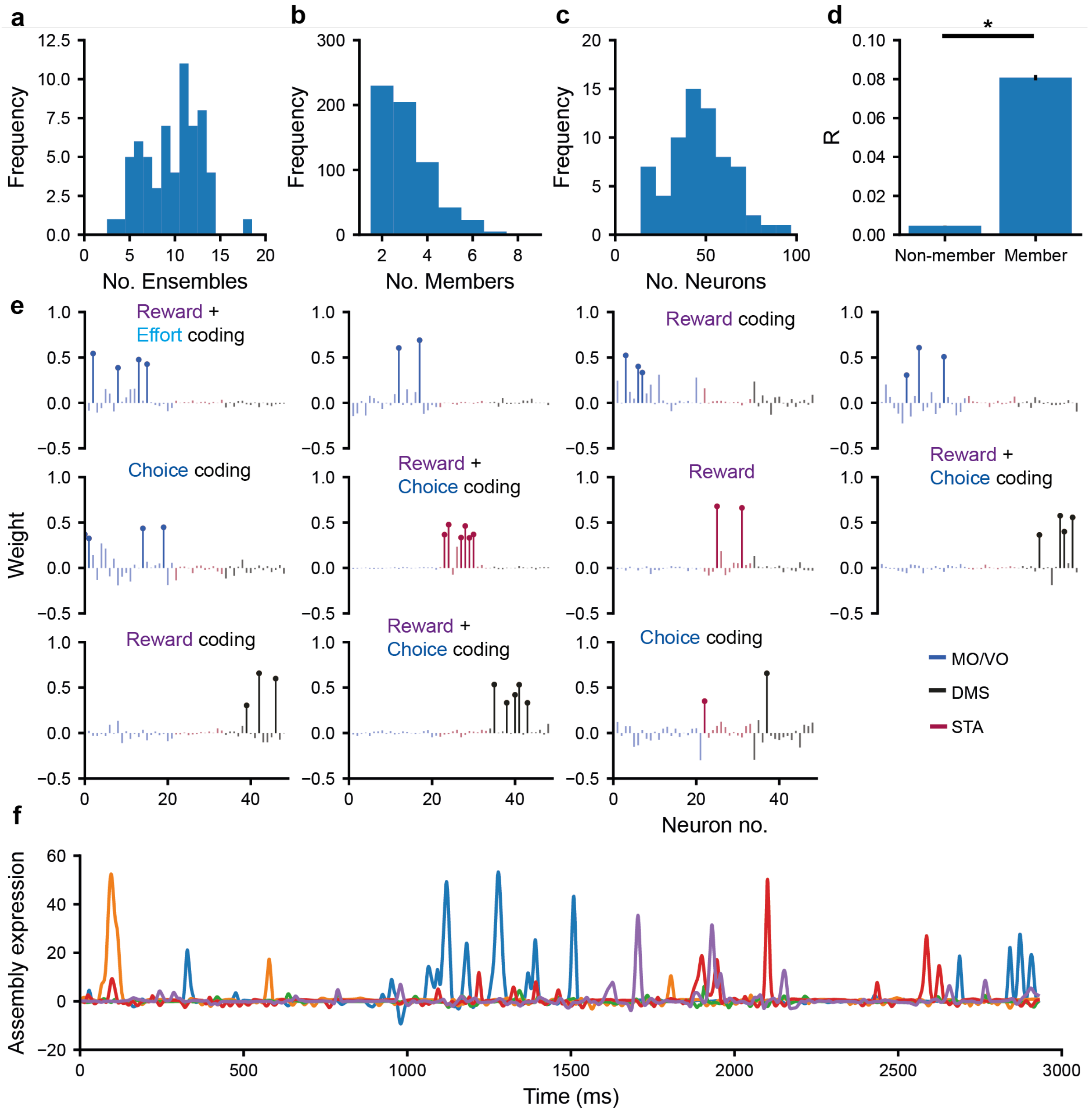

Supplemental Figure 8. An example session with 11 independent assemblies recorded simultaneously. a, the number of assemblies detected simultaneously per recording. A median of 10 assemblies were recorded simultaneously per session. **b,** the number of member neurons per assembly. This was in the range of 2-8. **c,** the number of neurons recorded simultaneously per session. This is included to contextualise the number of ensembles/member neurons. **d,** the Pearson's R between the spike trains (binned at 25 ms, from cue onset to the end of the action window for all trials) of pairs of member neurons (n=2415 pairs) is significantly greater than pairs of non-member neurons (n=79479 pairs) (Mann-Whitney U test, $p<10^{-20}$). **e,** the assembly patterns of 11 assemblies made of distinct patterns of neurons identified simultaneously in one recording. Member neurons are marked with a circle on top of the bar indicating their weight. In this session, we recorded single units in the MO/VO, DMS and STA. We identified 5 MO assemblies, 2 STA assemblies and 3 DMS assemblies. One assembly was identified with a STA and a DMS neuron as members. If assemblies significantly coded for reward level, effort level or choice outcome (Fig. 4h), this was marked with text above the assembly pattern. **f**, the assembly pattern expression strength in the 3s after cue onset of an example trial for the 5 assemblies in **c** which significantly coded for choice outcome (coloured arbitrarily for display purposes). As has previously been reported, expression strength had a stable baseline with sharp peaks. Although the 5 assemblies all encode choice outcome, they activate at distinctly different time periods.

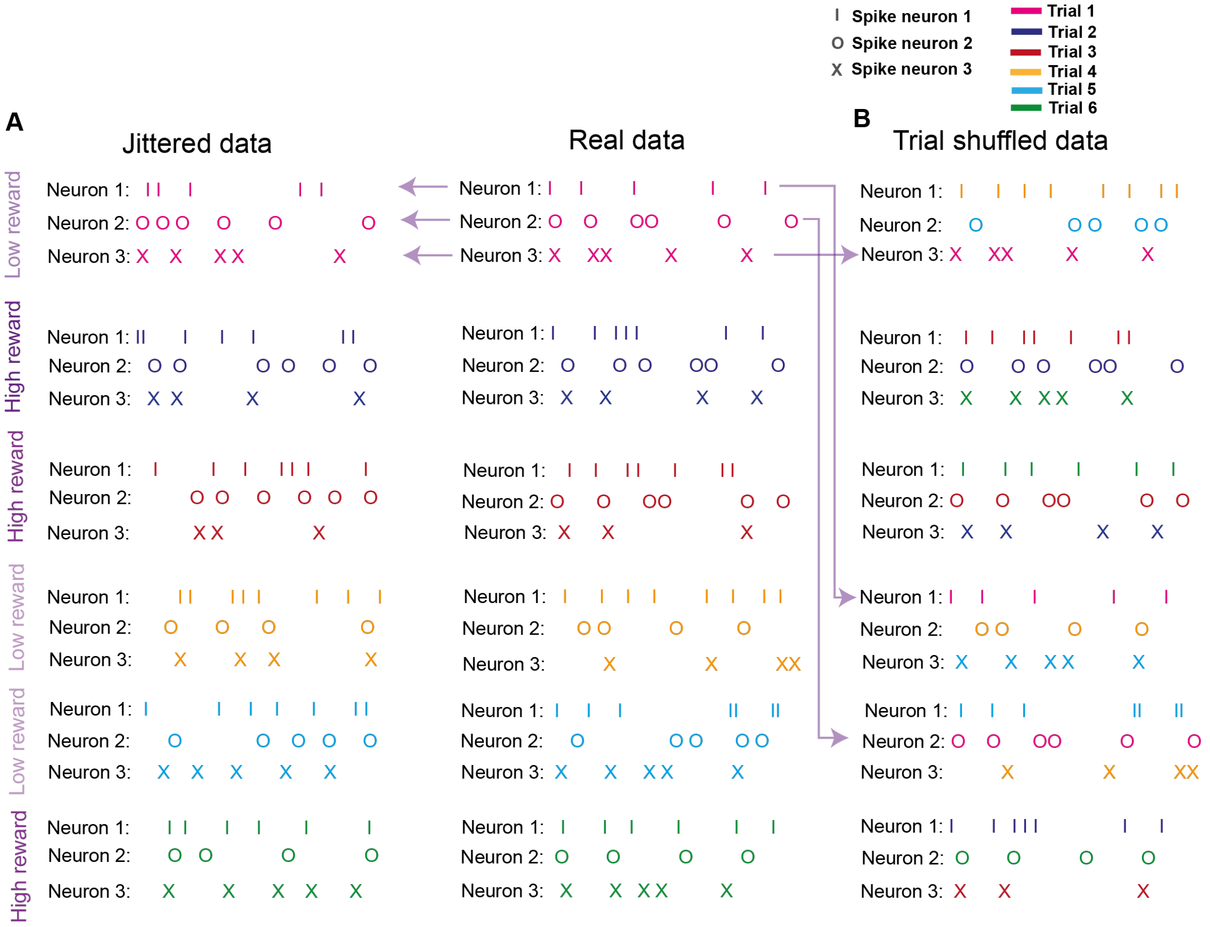

Supplemental Figure 9. A schematic showing the jittered spiking and trial shuffle controls for assembly coding. This schematic represents the spike trains of 3 hypothetical neurons (with spikes marked with a I, O and X) across 2 reward levels. Each trial is coloured distinctly (trial 1: pink, trial 2: dark blue, trial 3: red, trial 4: orange, trial 5: light blue and trial 6: green). **a,** For the random jittering control, the spike timings for each neuron were jittered by a random amount between -250ms and 250ms**.** This preserves slower changes in the rate of firing in neurons but disrupts fine timescale coactivity. In the schematic, the spike timings of each of the spike trains are changed but the overall firing rate remains largely the same. **b,** For the trial-by-trial shuffle control, the spike trains of each neuron was shuffled across trials of the same level of reward, effort and choice outcome. For instance, in the example above, the spike trains for each neuron are switched across trials of the same reward level (high reward trials are swapped with other high reward trials, and low reward trials are swapped with other low reward trials). This perfectly preserves the task triggered firing response (including the average rate response of each neuron to each reward level, effort level, or choice outcome) but breaks up any coactivity which does not result from the task structure. For reward coding assemblies, which show peak encoding around cue onset (**Fig 6**), we shuffled the spike trains from the 3s after cue onset for all neurons recorded. This controls for firing responses which are locked to the onset of the cue. The coactivity and coding properties of assemblies after the trial shuffle were compared to the coding of reward levels over the same time window (3s after cue onset) in the unshuffled data. For effort and choice coding assemblies, which show a more sustained coding/a peak encoding around the time of corridor entry (**Fig 6**), we shuffled spike trains from the 3s preceding adjusted corridor entry. Shuffling neural activity in this way controls for any coactivity that arises as a correlate of action initiation. The coactivity and coding properties of assemblies after the trial shuffle were compared to the coding of effort levels or choice outcome over the same time window (3s prior to adjusted corridor entry) in the unshuffled data.

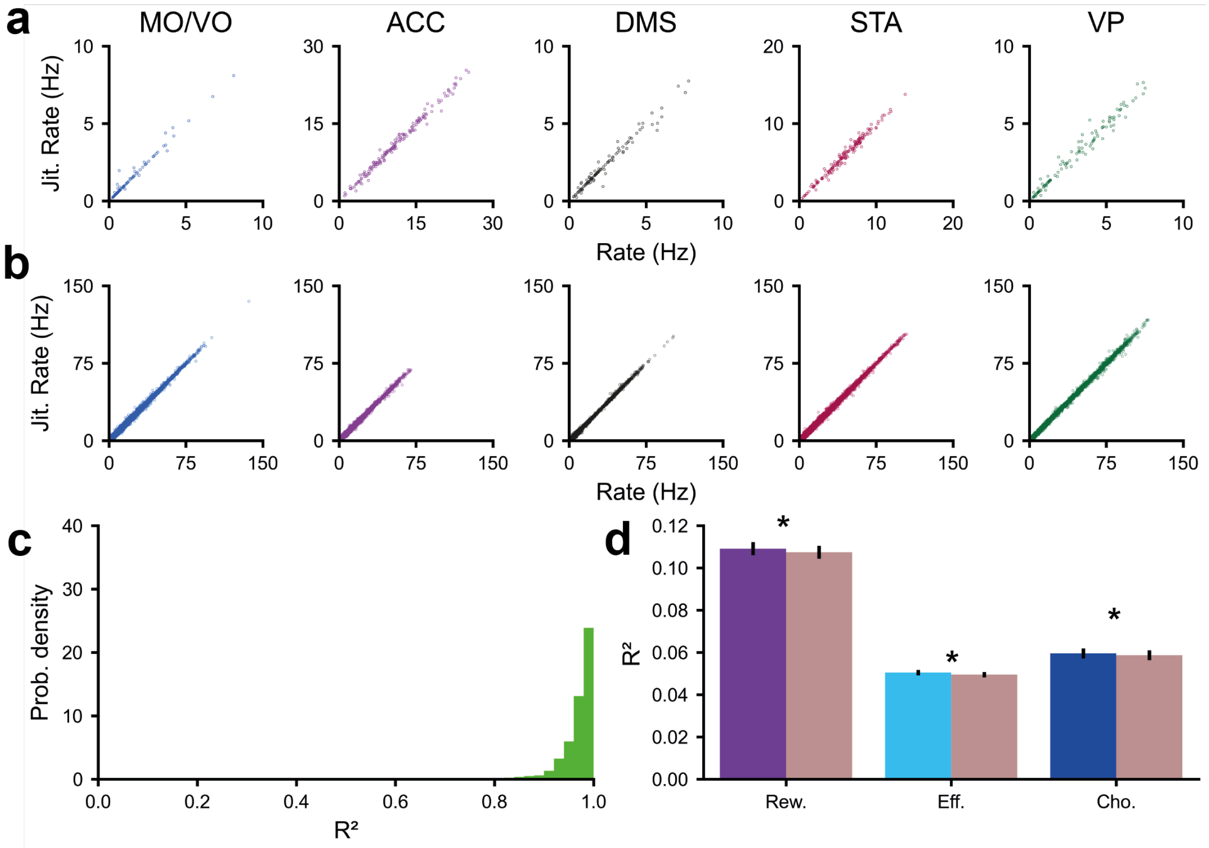

Supplemental Figure 10. Cofiring assemblies across the frontal-basal ganglia network represent combinations of reward, effort and decision. a, The trial-by-trial jittered firing rate plotted against the real firing rate for example neurons from the MO/VO, ACC, DMS, STA and VP. **b**, The trial-by-trial jittered firing rate plotted against the real firing rate pooled across all neurons in the MO/VO, ACC, DMS, STA and VP (brain structures ordered as in **a**). **c**, The distribution of the R^2 for the correlation between the trial-by-trial jittered firing rate and the real firing rate for each of the recorded neurons. **d**, The R^2 between the firing rate of unjittered (brown) as compared to jitttered spike trains (blue) in the decision window for reward (R^2 from simple linear regression, p= 5.7 x 10-6, Wilcoxon signed-rank test), effort (R^2 from simple linear regression, p = 2.1 x 10-5, Wilcoxon signed-rank test) and decision (pseudo-R^2 from logistic regression, p= .029, Wilcoxon signed-rank test) for significantly coding neurons pooling across all brain regions (significance determined using p-values from linear regressions with firing rate as a dependent variable and reward, effort and choice as independent variables). While significant, the change in R^2 was very low with almost all coding being preserved in the jittered spike trains. Rew. = reward, Eff. = effort, Cho.= choice.

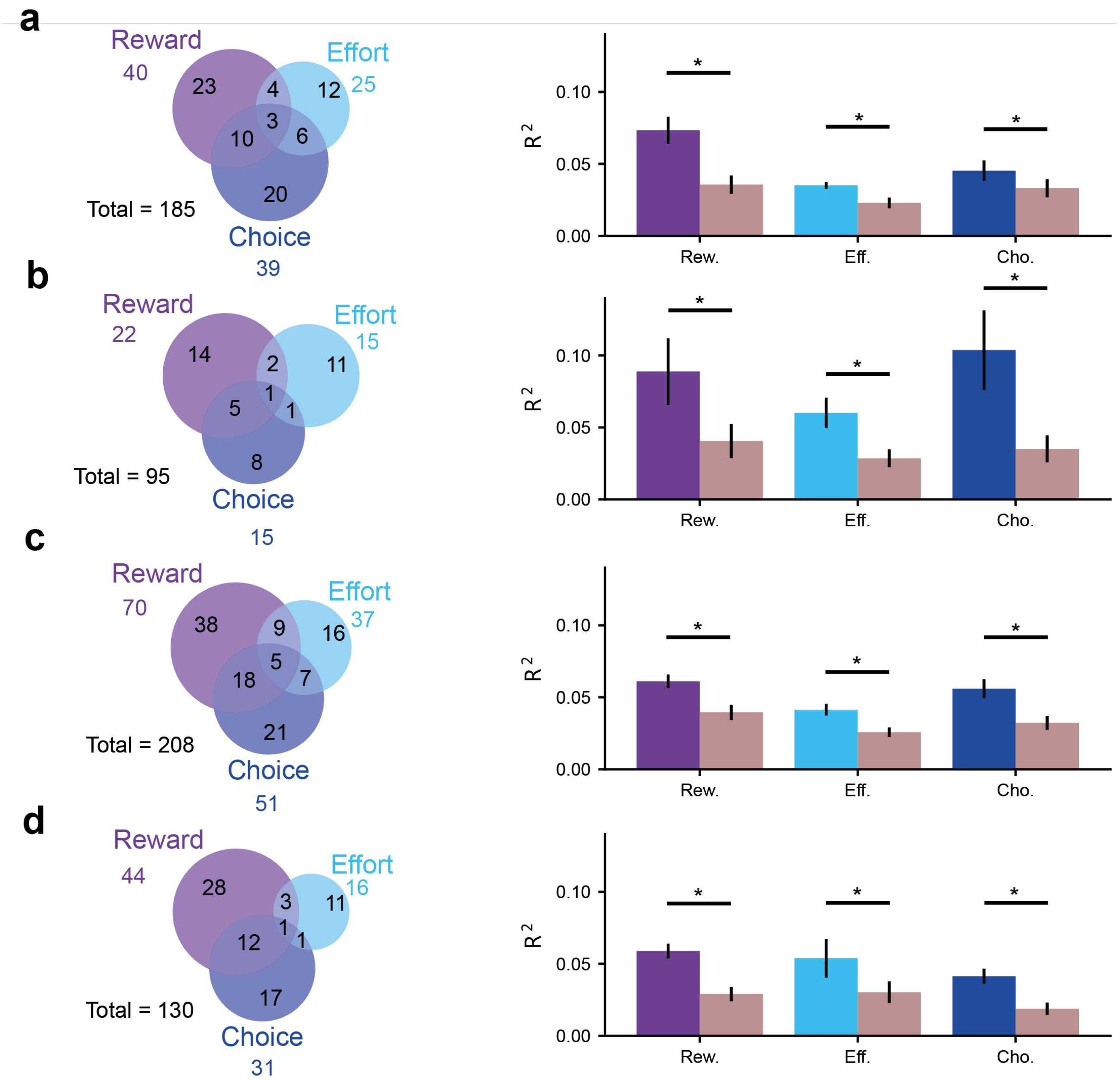

Supplemental Figure 11. Cofiring assemblies represent combinations of reward, effort and decision in all rats. a, b, c, d, Each row represents the encoding properties of assemblies in a single animal. Importantly, assemblies that represented reward, effort and choice outcome could be identified in all animals. Equally, encoding of these variables by assemblies was significantly disrupted by randomly jittering the spike timings, even when separating assemblies by which animal they were recorded in. **a-d, left,** Venn diagram showing the number of assemblies which have their coactivity significantly modulated by reward, effort and choice outcome separating by animal. **a-d, right,** The variance in reward (R^2 from simple linear regression), effort (R^2 from simple linear regression) or decision outcome (pseudo-R^2 from logistic regression) explained by assembly pattern expression strength with (brown) or without (blue) random jittering of spike trains in the range of -250ms to 250ms for significantly encoding assemblies separating by animal. Wilcoxon signed-rank test, $p<0.05$ for jittered vs. non-jittered for all variables across all animals. Rew. = reward, Eff. = effort, Cho.= choice.

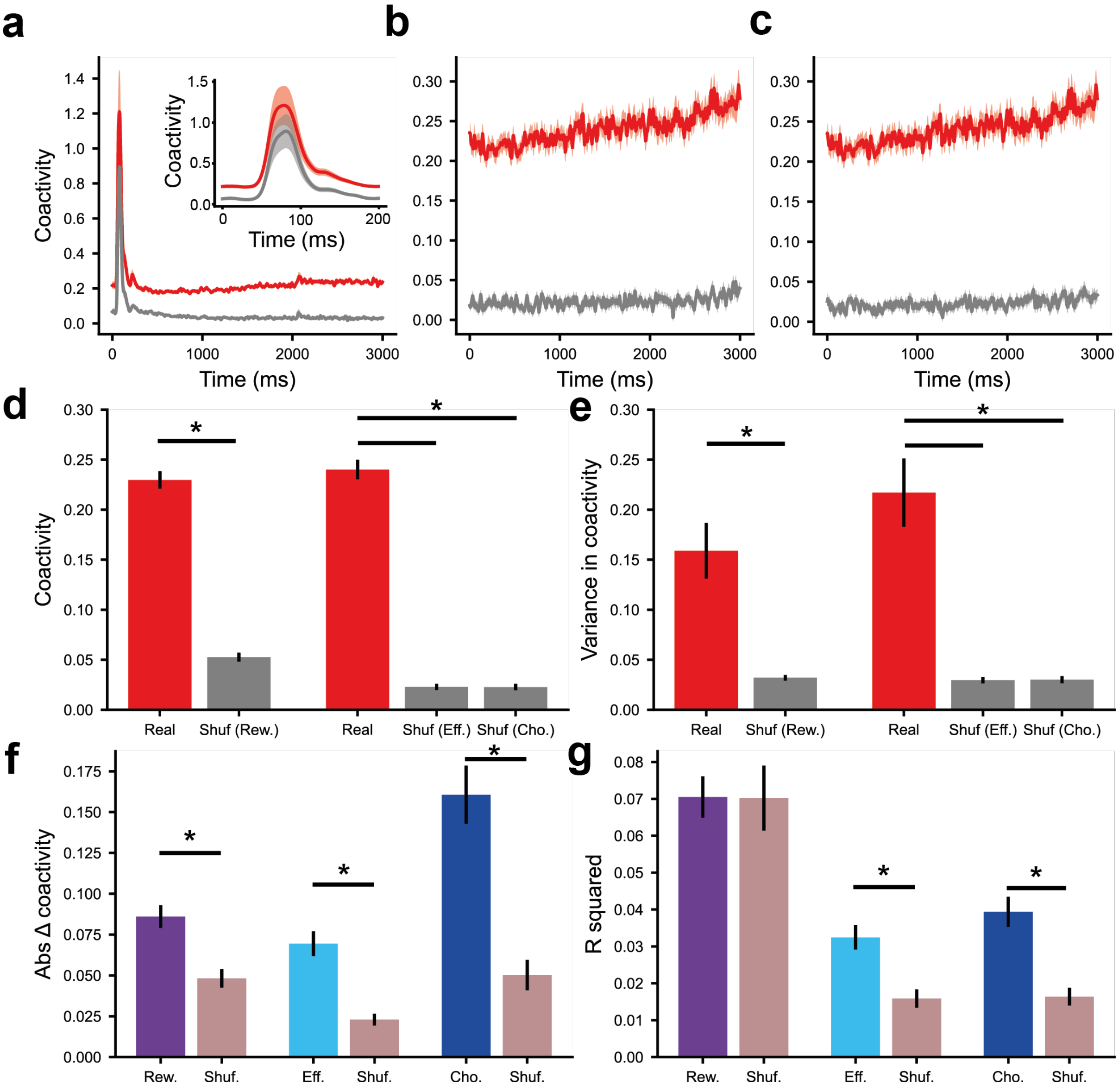

Supplemental Figure 12. The cofiring of assemblies does not arise only on account of task structure. This figure shows data from all assemblies across all structures. In order to investigate if the structure of the task was the sole driver of coactivity in assemblies, we shuffled spike trains for each neuron across trials and calculated the resulting coactivity. Specifically, for each neuron we shuffled the spike trains across trials with the same levels of reward, effort or choice outcome. This preserves the triggered firing responses to the structure of the task but removes any coactivity that does not result from the task structure itself. For reward coding assemblies which have peak encoding around cue onset, we shuffled spikes over the first 3s across trials. For effort and decision coding assemblies, which show peak coding around the time of corridor entry we shuffled spikes across trials for the 3s prior to adjusted corridor entry. **a**, the coactivity calculated in real data (red) as compared to spike trains shuffled across trials of the same reward level (grey). Time 0s is cue onset. We include an inset showing coactivity in real and trial-by-trial shuffled coactivity in the first 200ms after cue onset. **b**, the coactivity calculated in real data (red) as compared to spike trains shuffled across trials of the same effort level (grey). Time 3000ms is adjusted corridor entry. **c**, the coactivity calculated in real data (red) as compared to spike trains shuffled across trials of the same choice outcome (grey). Time 3000ms is adjusted corridor entry. **d**, the mean coactivity over the 3s in **a-c** was greater in real data (red) as compared to spike trains shuffled across trials of the same reward level (Wilcoxon signed-rank test, $p<10^{-20}$), effort level (Wilcoxon signed-rank test, $p<10^{-20}$) or choice outcome (Wilcoxon signed-rank test, $p<10^{-20}$) (grey). **e**, the mean variance in coactivity over 3s calculated in real data (red) as compared to spike trains shuffled across trials of the reward (Wilcoxon signed-rank test, $p<10^{-20}$), effort (Wilcoxon signed-rank

test, $p<10^{-20}$) or choice outcome (Wilcoxon signed-rank test, $p<10^{-20}$) (grey). **f,** the absolute beta coefficient from simple linear regression between the reward level, effort level and choice outcome and the coactivity in assemblies with or without shuffling in significant reward, effort and choice encoding assemblies (Fig 4h), respectively. Again, the real/shuffled spikes were from the 3s following cue onset for reward trial shuffling, whereas for effort and choice outcome the 3s prior to adjusted corridor entry were used. The change in coactivity across reward levels (Wilcoxon signed-rank test, $p<10^{-18}$), effort levels (Wilcoxon signed-rank test, $p<10^{-11}$) and choice outcomes (Wilcoxon signed-rank test, $p<10^{-16}$) was greater in real than shuffled data across all variables. **g,** The variance explained in the reward level, effort level and choice outcome by the coactivity in assemblies with or without shuffling in significant reward, effort and choice encoding assemblies (Fig 4), respectively. For reward and effort, a simple linear regression was used, whereas for choice outcome a logistic regression was utilised. Again, the real/shuffled spikes were from the 3s following cue onset for reward trial shuffling, whereas for effort and choice outcome the 3s prior to adjusted corridor entry were used. The variance explained of effort (R squared, Wilcoxon signed-rank test, $p<10^{-4}$) and choice outcome (pseudo-R squared, Wilcoxon signed-rank test, $p<10^{-9}$) by coactivity for the effort and choice coding assemblies respectively was significantly greater for real than shuffled data. There were no significant differences between the variance in reward explained by coactivity in real and shuffled data for reward coding assemblies (Wilcoxon signed-rank test, $p=0.07$). This suggests that much of the coactivity that drives reward encoding is driven by cue-locked neural activity. Rew. = reward, Eff. = effort, Cho.= choice.

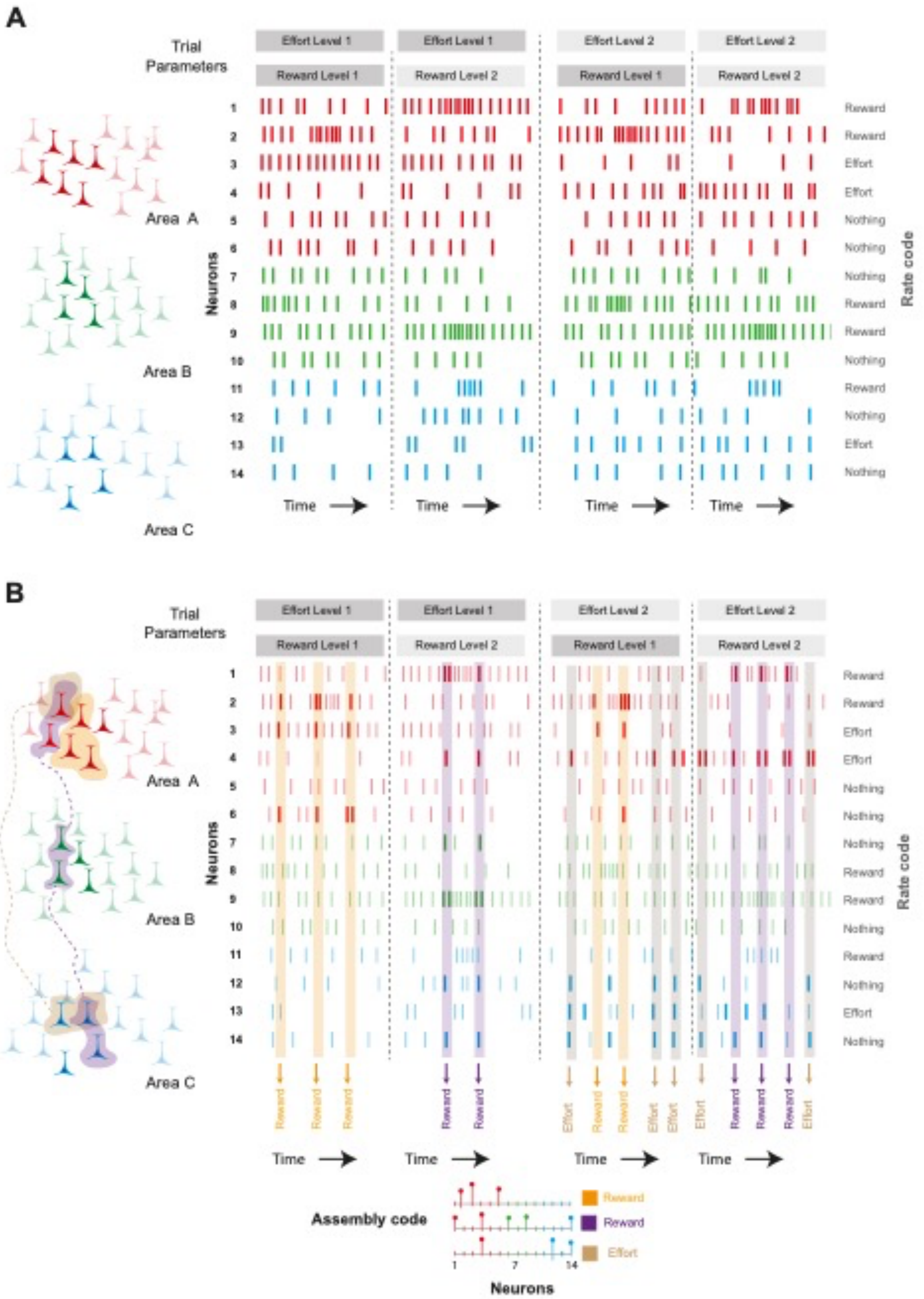

Supplemental Figure S13. Schematic adapted[1] representation of relationships between detected rate and assembly codes and their possible relationships to the wider network. a, Schematic representation of rate responses in 3 populations of neurons (denoted by colour) from different brain areas during trials with different combinations of prospective reward and effort. Neurons can encode the decision parameters and/or transient rate changes that occur consistently in part of the trial (e.g. neurons 1 and 2) and/or global changes in firing rate during specific conditions (e.g. neurons 3 and 4). The recorded neurons (in bold) are assumed to reflect the rate-responses

of the wider population (faded) within that structure. b, Assembly-coding can occur simultaneously during the rate coding shown in A. Neurons with all types of rate coding (reward, effort, nothing) can contribute to assemblies that encode specific combinations of effort and reward. Spikes that contribute/or a consequence of assemblies can occur inside or outside or rate-coding periods. Assemblies can be formed within and/or across different areas and a given neuron can participate in assemblies encoding different parameters (e.g. neuron 14). The recorded neurons (bold) represent a small part of the "real" assembly which likely comprises many neurons in the wider of population of neurons within and outside the recorded structures.

References

1 El-Gaby, M. *et al.* An emergent neural coactivity code for dynamic memory. *Nat Neurosci* **24**, 694-704 (2021). https://doi.org:10.1038/s41593-021-00820-w
